# A versatile way for the synthesis of monomethylamines by reduction of *N*-substituted carbonylimidazoles with the NaBH_4_/I_2_ system

**DOI:** 10.3762/bjoc.18.104

**Published:** 2022-08-17

**Authors:** Lin Chen, Xuan Zhou, Zhiyong Chen, Changxu Wang, Shunjie Wang, Hanbing Teng

**Affiliations:** 1 School of Chemistry, Chemical Engineering and Life Sciences, Wuhan University of Technology, 122 Luoshi Road, Wuhan, 430070, China

**Keywords:** amines, carboxylic acids, isocyanates, monomethylamines, *N*-substituted carbonylimidazoles, reduction

## Abstract

An economical and versatile protocol for the one-pot synthesis of monomethylamines by reduction of *N*-substituted carbonylimidazoles with NaBH_4_/I_2_ in THF at reflux temperature is described. This method used no special catalyst and various monomethylamines can be easily obtained in moderate to good yields from a wide range of raw materials including amines (primary amines and secondary amines), carboxylic acids and isocyanates. Besides, an interesting reduction selectivity was observed. Exploration of the reaction process shows that it undergoes a two-step pathway via a formamide intermediate and the reduction of the formamide intermediate to monomethylamine as the rate-determining step. This work can contribute significantly expanding the applications of *N*-substituted carbonylimidazoles.

## Introduction

*N*-Methylamines are widely found in natural products, fine chemicals, agrochemicals, pharmaceuticals and dyes [1–4]. Traditional methods for the preparation of *N*-methylamines involve the direct methylation of amines by using methyl halides [5–7], dimethyl sulfate [8], diazomethane [9], methyl triflate [10–11] or dimethyl carbonate [12–15] as the methylation reagents and the reductive amination reactions by using formaldehyde or paraformaldehyde as the “indirect” alkylation reagents [16–19]. Recently, a variety of promising methylating agents or C1 sources such as formic acid [20–21], methanol [22–31] and carbon dioxide (CO_2_) [32–39] have been developed for the *N*-methylation of amines. However, these *N*-alkylation methods often require the employment of expensive catalysts, and the *N*-alkylation of primary amines generally does not stop with monomethylation as expected and inevitably provides a mixture of multiple methylated products because of the competing overalkylation reactions [14,16–43].

In order to obtain pure monomethylation product, the conventional method is to introduce alkyl formate, formacyl, methylene or their equivalents to amines, followed by reduction to give monomethylamines [44–51]. Protection/methylation/deprotection strategies have also been developed for the preparation of monomethylation objects, which are particularly suitable for peptide chemistry since protecting groups are often required in peptide synthesis [52–53]. These multistep reaction methods are conducive to avoid overmethylation products.

**Scheme 1:**
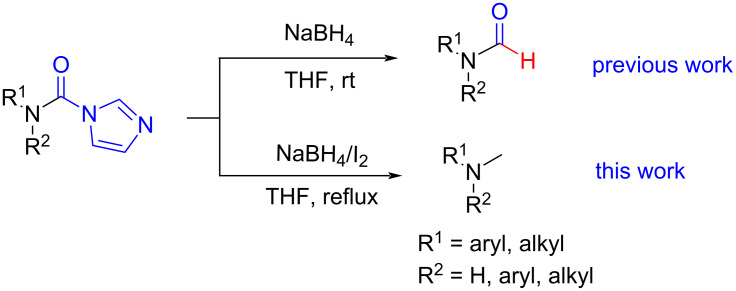
The synthesis of formamides and monomethylamines.

Although procedures for the synthesis of monomethylamines have been developed over the past years, the starting materials are mainly restricted regarding amines, in addition, expensive reagents or catalysts are usually required, which limit their applications to some extent. *N*-Substituted carbonylimidazoles, as important members of the carbonylimidazole family [54–55], are highly attractive intermediates with suitable stability for isolation or storage, and various good methods for their perparation have been developed by employing different starting materials such as amines [56–58], isocyanates [59–61] and carboxylic acids [62]. Since the carbonyl carbon atom of the carbonylimidazole moiety is easily attacked by a nucleophile the imidazole group readily dissociates. The *N*-substituted carbonylimidazoles have favorable reactivity and can be widely used in the synthesis of various valuable products such as ureas [63–70], carbamates [66,71–74], thiocarbamates [66], and cyanoformamides [75]. However, all of these works are primarily focused on the substitution reaction of *N*-substituted carbonylimidazoles. In our previous work, we conveniently prepared formamides by reducing *N*-substituted carbonylimidazoles with NaBH_4_ [62] (Scheme 1). The reaction mechanism shows that the H^−^ ion acted as a nucleophile to attack the carbonyl carbon to cause the imidazolium ion to leave without reducing the carbonyl group. Although this work expands the application of *N*-substituted carbonylimidazoles, the reaction can still be regarded as a substitution reaction, which is attributed to the weak reducibility of NaBH_4_.

In this work, our goal is to reduce the carbonyl group in *N*-substituted carbonylimidazoles. The inexpensive NaBH_4_/I_2_ system has great attraction because it is more reductive due to the generation of highly reactive BH_3_–THF by adding iodine to NaBH_4_ in THF [76–78] and the reaction conditions are not significantly changed compared to our previous preparation of formamide. With this reduction system, we achieved a one-step conversion from *N*-substituted carbonylimidazoles to methylamines. This interesting work will help to synthesize pure monomethylamines from a wide range of raw materials including amines, carboxylic acids and isocyanates under mild and safe reaction conditions.

## Results and Discussion

Initially, *N*-phenethyl-1*H*-imidazole-1-carboxamide (**1b**) was chosen as a model substrate to react with 3.0 equiv of NaBH_4_ and 1.0 equiv of I_2_ in THF at reflux temperature, as expected, the carbonylimidazole moiety was successfully converted into a methyl group and the target monomethylamine (**1c**) was obtained in 70% yield after 6 h (Table 1, entry 1). When the amount of NaBH_4_ was increased from 3.0 equiv to 4.0 equiv and 5.0 equiv, the reaction time was shortened from 6 h to 4 h and 1 h, respectively. When further increasing the amount of NaBH_4_ to 6.0 equiv, only a slight decrease of the reaction time was observed. In addition, the yield of **1c** showed few changes with the increase of the amount of NaBH_4_ from 3.0 equiv to 6.0 equiv. Obviously, 5.0 equiv of NaBH_4_ was optimal to perform the reaction. Since I_2_ was used to improve the reducibility of NaBH_4_, we next investigated the effect of the amount of I_2_ on the reaction. The use of 0.5 equiv of I_2_ in the presence of 5.0 equiv of NaBH_4_ afforded after 6 h only traces of methylamine but 62% of *N*-(phenethyl)formamide (Table 1, entry 5). Increasing the amount of I_2_ from 1.0 equiv to 1.5 equiv (Table 1, entry 6) did not significantly accelerated the reaction. All the above described results might indicate that an assembly of 1.0 equiv of I_2_ and 5.0 equiv of NaBH_4_ was sufficient to complete the reaction in one hour.

**Table 1:** Optimization of the reaction conditions.^a^

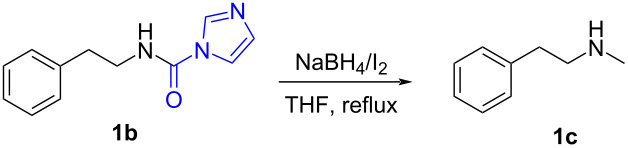				

Entry	NaBH_4_ (equiv)	I_2_ (equiv)	Time (h)^b^	Yield (%)^c^

1	3.0	1.0	6	70
2	4.0	1.0	4	72
3	5.0	1.0	1	74
4	6.0	1.0	0.9	75
5	5.0	0.5	6	trace^d^
6	5.0	1.5	1	73

^a^The reactions were carried out with **1b** (1.0 equiv, 2 mmol), NaBH_4_, I_2_, THF (25 mL) under reflux temperature. ^b^The reaction was monitored by TLC. ^c^Isolated yield was based on **1b**. ^d^The isolated yield of **1c** and *N*-(phenethyl)formamide was 1% (trace) and 62%, respectively.

With the optimized reaction conditions in hand, we investigated the synthesis of other *N*-methylamines from various *N*-substituted carbonylimidazoles (Table 2). As a proof of the versatility and applicability of the proposed method, *N*-substituted carbonylimidazoles were prepared from amines (**1b–14b**) [56–58,62,64], isocyanates (**15b–17b**) [59–61], and carboxylic acids (**18b–20b**) [62], respectively (for detailed experimental procedures, see Supporting Information File 1). All types of these *N*-substituted carbonylimidazoles reacted smoothly with NaBH_4_/I_2_ to provide the corresponding *N*-methylamines in satisfactory yields.

**Table 2:** Synthesis of monomethylamines from amines, carboxylic acids and isocyanates.^a^

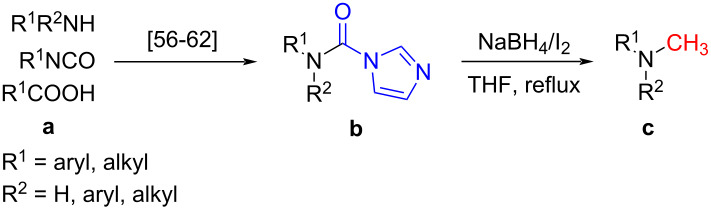					

Entry	Substrate	Carbamoylimidazole	Time (h)^b^	Product	Yield (%)^c^

1	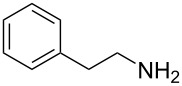 **1a**	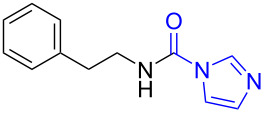 **1b**, 87%	1	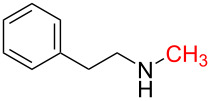 **1c**	74
2	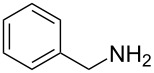 **2a**	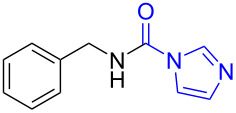 **2b**, 85%	1	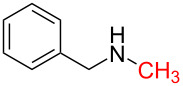 **2c**	67
3	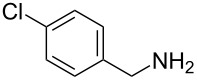 **3a**	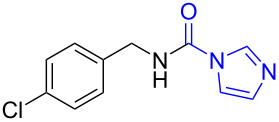 **3b**, 89%	1	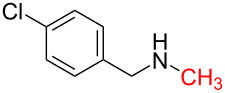 **3c**	67
4	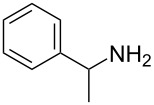 **4a**	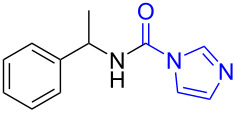 **4b**, 77%	1	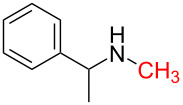 **4c**	65
5	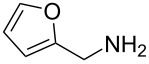 **5a**	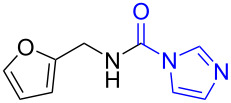 **5b**, 87%	1	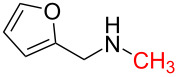 **5c**	70
6	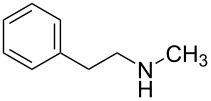 **1c**	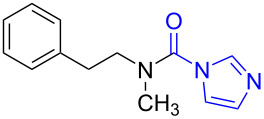 **6b**, 88%	1	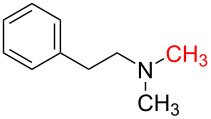 **6c**	83
7	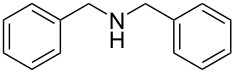 **7a**	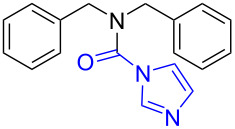 **7b**, 85%	2	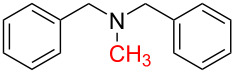 **7c**	60
8	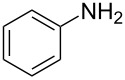 **8a**	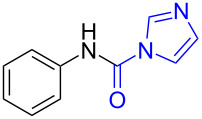 **8b**, 70%	4	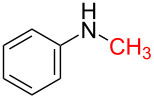 **8c**	72
9	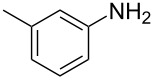 **9a**	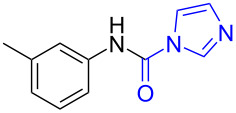 **9b**, 78%	4	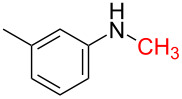 **9c**	85
10	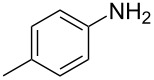 **10a**	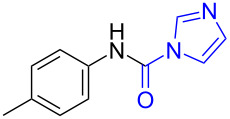 **10b**, 75%	4	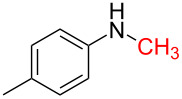 **10c**	80
11	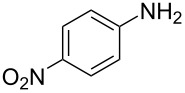 **11a**	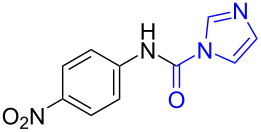 **11b**, 55%	4	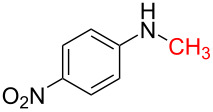 **11c**	70
12	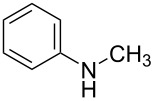 **8c**	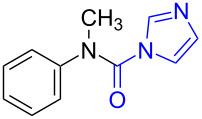 **12b**, 89%	4	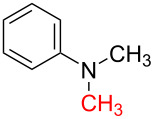 **12c**	71
13	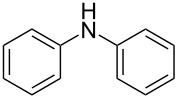 **13a**	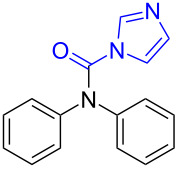 **13b**, 57%	6	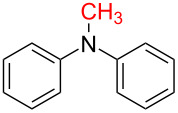 **13c**	67
14^d^	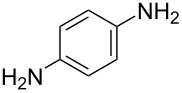 **14a**	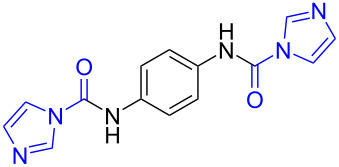 **14b**, 83%	10	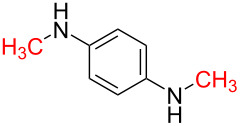 **14c**	70
15	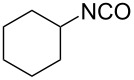 **15a**	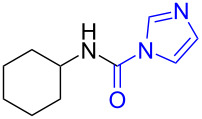 **15b**, 95%	1	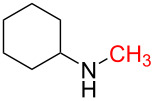 **15c**	74
16	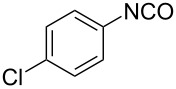 **16a**	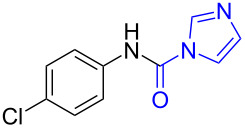 **16b**, 97%	4	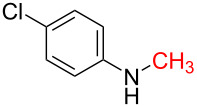 **16c**	74
17	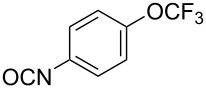 **17a**	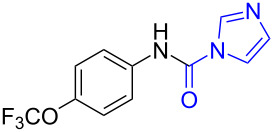 **17b**, 94%	4	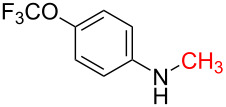 **17c**	72
18	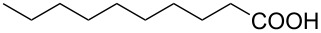 **18a**	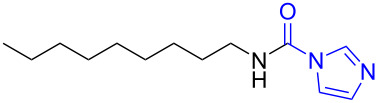 **18b**, 72%	1	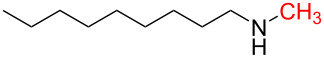 **18c**	77
19	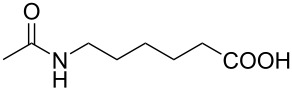 **19a**	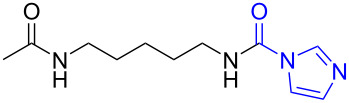 **19b**, 91%	1	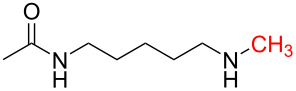 **19c**	65
20	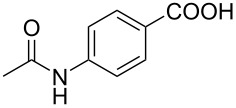 **20a**	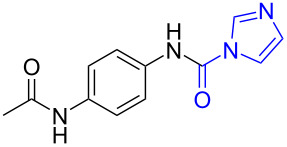 **20b**, 83%	4	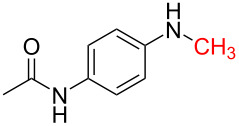 **20c**	68

^a^The reactions were carried out with carbamoylimidazole (1.0 equiv, 2 mmol), NaBH_4_ (5.0 equiv, 10.0 mmol), I_2_ (1.0 equiv, 2 mmol) and THF (25 mL) under reflux. ^b^Monitored by TLC. ^c^Isolated yield was based on carbamoylimidazole. ^d^10.0 equiv of NaBH_4_ and 2 equiv of I_2_ was used.

The impact of different substituents on the reaction were well investigated. As shown in Table 2, the alkyl substituents (R) in the *N*-alkyl carbonylimidazoles had a weak influence on the reaction (Table 2, entries 1–7, 18, and 19). Whether the *N*-alkyl carbonylimidazoles bear one substituent (e.g., **1b–5b**, **15b**, **18b**, **19b**) or two substituents (e.g., **6b** and **7b**), the reaction proceeded well, affording the desired product in 60–83% yields. Note that the reaction time of **7b** (2 h) was obviously longer than that of **1b–6b** (1 h), **18b** (1 h) and **19b** (1 h), possibly because the steric hindrance of the two benzyl groups on **7b** slowed the reaction. Encouraged by the above mentioned results, we then tested *N*-aryl carbonylimidazoles in the reaction. To our delight, *N*-aryl carbonylimidazoles with either electron-donating (**9b** and **10b**) or electron-withdrawing groups (**11b**, **16b** and **17b**) on the aryl rings were all transformed, affording the expected products in 70–85% yields. *N*-Aryl carbonylimidazoles with two substituents, such as **12b** (R^1^ = methyl, R^2^ = phenyl) and **13b** (R^1^ = phenyl, R^2^ = phenyl), were also amenable to this protocol, giving the corresponding products **12c** and **13c** in 71% and 67% yield, respectively. Furthermore, by using 2.0 equiv of I_2_ and 10.0 equiv of NaBH_4_, the substrate **14b** with two *N*-substituted carbonylimidazole moieties could also undergo this reaction and provided the desired product **14c** in moderate yield (70%). Additionally, our protocol was applicable to prepare *N,N*-dimethylamines by step-by-step methylation. Employing the mono-methylated products **1c** and **8c**, *N,N*-dimethylamine **6c** and **12c** can be obtained, respectively, via repeating our synthesis procedure.

In order to understand the reduction selectivity of the method, the substrates bearing acetamide groups (**19b** and **20b**) were tested in the reaction. To our pleasure, both aliphatic and aromatic amides reacted smoothly and provided the expected products in satisfactory yields, with the acetyl groups being unaffected. This suggested that the *N*-acetyl groups in *N*-substituted carbonylimidazoles were well tolerated during the reduction, and our method showed interesting reduction selectivity.

**Scheme 2:**
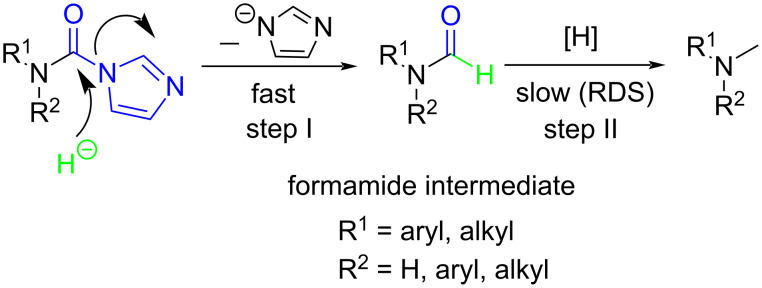
The possible reaction mechanism. RDS = rate determining step.

To gain some preliminary insight into the reaction process, two representative intermediates for the synthesis of aliphatic methylamine **1c** and aromatic methylamine **8c** had been isolated and identified as corresponding formamides (see Supporting Information File 1). Furthermore, by follwoing the reaction with TLC, we found that the reaction time (hours) from the formamide intermediate to the corresponding methylamine product was much longer than the time (minutes) from the *N*-substituted carbonylimidazole to the formamide. This indicated that the reaction might undergo a two-step pathway via the formamide intermediate (Scheme 2).

In the first step (step I), *N*-substituted carbonylimidazole was rapidly converted into the formamide intermediate by the attack of a hydrogen anion as we had reported before [62]. Subsequently, the carbonyl group of the formamide intermediate was reduced to furnish the desired *N*-methylamine in the second step (step II) [79–81]. Step II proceeded much slower than step I, so it could be treated as rate-determining step (RDS). The required longer reaction time for *N*-aryl carbonylimidazoles (over 4 h) than that for *N*-alkyl carbonylimidazoles (about 1 h) can be well explained by the two-step mechanism. In step I, the *N*-aryl carbonylimidazoles might react much faster than *N*-alkyl carbonylimidazoles, because the stronger conjugation system of the resulting *N*-aromatic formamides made them more stable and easier to generate. However, these more stable *N*-aryl formamide intermediates were less reactive and directly slowed the reaction in step II, which resulted in a longer reaction time of the *N*-aryl carbonylimidazoles in the whole reaction.

The substrate **13b**, bearing two phenyl rings, which not only had a large steric hindrance like **7b**, but also had a strong conjugation system, took much longer time (6 h) to complete the reaction.

As shown by the reaction mechanism, the methyl group was converted from the carbonylimidazole moiety by full reduction and therefore no competing overalkylation reactions occurred.

Although the *N*-methylamine could be prepared from carboxylic acid or amine by our method, the methyl source was remarkably different (Scheme S1, Supporting Information File 1). For amines, the carbon source of the methyl group is from the carbonyl group of the carbonyldiimidazole; while for carboxylic acids, the carbon source is the carboxyl group. When carboxylic acids were used, the carboxyl moiety was first converted to an isocyanate via Curtius rearrangement [82–85], then reacted with imidazole to form the carbonylimidazole, and eventually reduced to the methyl moiety. In the whole process, no extra carbon was introduced.

## Conclusion

In conclusion, we have developed an economical and versatile protocol for the one-pot synthesis of monomethylamines by reduction of *N*-substituted carbonylimidazoles with the NaBH_4_/I_2_ system. This work further extends the application of *N*-substituted carbonylimidazoles. By employing inexpensive and commercially available reagents, a variety of aliphatic and aromatic monomethylamines were obtained in moderate to good yields from a broad substrate scope including not only amines (both primary amines and secondary amines) but also carboxylic acids or isocyanates. The acetamide group was well tolerated in our reduction, implying our method showed interesting reduction selectivity.

## Supporting Information

File 1 Experimental part.
