# Supplementary material for: A versatile way for the synthesis of monomethylamines by reduction of N-substituted carbonylimidazoles with the NaBH4/I2 system
Source: Beilstein J Org Chem. 2022 Aug 17;18:1032–9. doi: 10.3762/bjoc.18.104 (PMC9443423; doi:10.3762/bjoc.18.104)

BEILSTEIN JOURNAL OF ORGANIC CHEMISTRY

Supporting Information

for

A versatile way for the synthesis of monomethylamines by reduction of *N*-substituted carbonylimidazoles with the $NaBH_4/I_2$ system

Lin Chen, Xuan Zhou, Zhiyong Chen, Changxu Wang, Shunjie Wang and Hanbing Teng

Beilstein J. Org. Chem. **2022,** *18,* 1032–1039. doi:10.3762/bjoc.18.104

Experimental part

License and Terms: This is a supporting information file under the terms of the Creative Commons Attribution License (https://creativecommons.org/licenses/by/4.0). Please note that the reuse, redistribution and reproduction in particular requires that the author(s) and source are credited and that individual graphics may be subject to special legal provisions.
The license is subject to the *Beilstein Journal of Organic Chemistry* terms and conditions: (https://www.beilstein-journals.org/bjoc/terms)

Table of Contents

1. General information..S2

2. Synthesis of *N*-substituted carbonylimidazoles…...S2

3. General procedure for the preparation of *N*-methylamines...................................S6

4. Data of the two intermediates…………………..……………………………..….S15

5. Scheme S1..S18

6. References..S19

7. ^{1}H and ^{13}C NMR spectra of b and c..S21

8. ESI–MS..S45

1. General information

All starting materials and solvents were obtained from commercial suppliers and were used without further purification unless otherwise stated. Reagents were used as purchased. NMR spectra were recorded on Bruker spectrometers operating at 400 MHz (^{1}H resonance). Proton chemical shifts (δ) were given in parts per million (ppm) relative to tetramethylsilane (TMS) with the solvent resonance as internal standard: $CDCl_3$, δ = 7.26 ppm. Carbon chemical shifts were reported in ppm (δ) relative to TMS with the solvent resonance as internal standard: $CDCl_3$, δ = 77.16 ppm. Electron spray ionization mass spectra (ESI-MS) were recorded with a Shimadzu LC/MS–IT–TOF hybrid mass spectrometer (Kyoto, Japan) (**1c**–**3c**, **5c**–**7c**, **10c**, **14c**, **18c**–**20c**) and a Bruker MicroTOF-Q ESI time-of-flight system (**4c**, **8c**, **9c 11c**–**13c**, **15c**–**17c**). Solutions were evaporated under reduced pressure with a rotary evaporator. Thin-layer chromatography (TLC) was performed on precoated plates silica gel GF 254; the spots were visualized under UV light (λ = 254 nm) and/or Ninhydrin (aq.) was used as revealing system.

2. Synthesis of *N*-substituted carbonylimidazoles

2.1 General procedure for the preparation of *N*-substituted carbonylimidazoles (1b–12b, 14b) starting from amines [1-4].

To a solution of amine (4.0 mmol) in THF (30 mL) was slowly added carbonyldiimidazole (CDI) (4.8 mmol) and the solution was heated to refluxed for 1–5 h. The reaction was monitored by TLC. After completion of the reaction, the mixture was concentrated under reduced pressure. The residue was extracted with CH_2Cl_2 (100 mL), washed with water and dried over anhydrous Na_2SO_4. The solvent

was evaporated under reduced pressure and the crude product was purified by column chromatography.

Typical compound data are as follows：

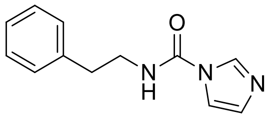

1b

***N*-Phenethyl-1*H*-imidazole-1-carboxamide** (**1b**) Yield: 87% (748 mg); white solid; R_f = 0.6 (EtOAc/PE, 2.5/1, v/v). ^{1}H NMR (400 MHz, $CDCl_3$, ppm): δ = 8.00 (s, 1H), 7.73 (t, *J* = 5.6 Hz, 1H), 7.41-7.42 (m, 1H), 7.33 – 7.20 (m, 5H), 6.89 (s, 1H), 3.65 (dd, *J* = 12.0 Hz, 8.0 Hz, 2H), 2.95 (t, *J* = 8.0 Hz, 2H).

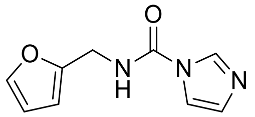

5b

***N*-(Furan-2-ylmethyl)-1*H*-imidazole-1-carboxamide** (**5b**) [5] Yield: 87% (666 mg); white solid; R_f = 0.6 (EtOAc/PE, 2.5/1, v/v). ^{1}H NMR (400 MHz, $CDCl_3$,ppm): δ = 8.24 (s, 1H), 7.42 (s, 1H), 7.37 (d, *J* = 0.8 Hz, 1H), 7.17 (br s, 1H), 7.03 (s, 1H), 6.35 – 6.32 (m, 2H), 4.59 (d, *J* = 4.0 Hz, 2H).

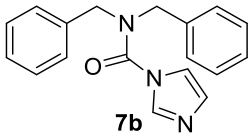

7b

***N,N*-Dibenzyl-1*H*-imidazole-1-carboxamide** (**7b**) [6] Yield: 85% (990 mg); white solid; R_f = 0.6 (EtOAc/PE, 2.5/1, v/v). ^{1}H NMR (400 MHz, $CDCl_3$, ppm): δ = 7.98 (s, 1H), 7.44 – 7.35 (m, 6H), 7.28 – 7.26 (m, 5H), 7.06 (s, 1H), 4.61 (s, 4H).

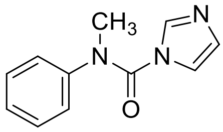

12b

***N*-Methyl-*N*-phenyl-1*H*-imidazole-1-carboxamide** (**12b**) [2] Yield: 89% (716 mg); white solid; R_f = 0.6 (EtOAc/PE, 2.5/1, v/v). ^{1}H NMR (400 MHz, $CDCl_3$, ppm): δ = 7.55 (s, 1H), 7.35 (t, *J* = 7.2 Hz, 2H), 7.28 (t, *J* = 7.2 Hz, 1H), 7.09 (d, *J* = 7.2 Hz, 2H), 6.81 (s, 1H), 6.77 (s, 1H), 3.46 (s, 3H).

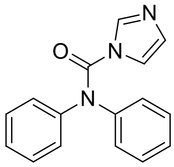

13b

***N*, *N*-Diphenylimidazole-1-carboxamide (13b)** [1].

To a solution of diphenylamine (1.69 g, 10.0 mmol), DBU (1.83 g, 12 mmol) in acetonitrile (60 mL) was added carbonyldiimidazole (CDI) (1.95 g, 12.0 mmol) and refluxed for 48 h. The reaction did not go to completion. Volatiles were distilled out under reduced pressure. The residue was extracted with CH_2Cl_2 (200 mL), washed with water and dried over anhydrous Na_2SO_4. The solvent was evaporated under reduced pressure and crude product was purified by column chromatography (R_f = 0.5, EtOAc/ PE, 2/1, v/v) to get white solid; Yield: 57% (1.5 g). ^{1}H NMR (400 MHz, $CDCl_3$, ppm): δ = 7.74 (s, 1H), 7.38 (t, *J* = 7.6 Hz, 4H), 7.29 (t, *J* = 7.6 Hz, 2H), 7.18 (d, *J* = 7.2 Hz, 4H), 7.00 (s, 1H), 6.88 (s, 1H).

2.2 General procedure for the preparation of *N*-substituted carbonylimidazoles (15b–20b) from isocyanates or carboxylic acids [7,8].

Carboxylic acid (10 mmol) was dissolved in dry THF (30 mL) and cooled to −10 °C to −15 °C. After addition of ethyl chloroformate (11 mmol) and Et_3N (12 mmol), the mixture was stirred for 20 min. A solution of NaN_3 (25 mmol) in H_2O (5 mL) was added and stirred for 1 h at −10 °C to −15 °C. The solution was then diluted with H_2O and extracted with EtOAc (150 mL). The organic layers were washed with brine (2 × 10 mL), dried over Na_2SO_4 and concentrated under reduced pressure to give crude acyl azide. The crude acyl azide was dissolved in toluene (35 mL) and heated to 80 °C under stirring for 2–3 h. The toluene was removed under reduced pressure to afford isocyanate.

Isocyanate (5 mmol) synthesized according to the above method or obtained from commercial suppliers was dissolved in CH_2Cl_2 (15 mL) at room temperature. The imidazole (5.5 mmol) dissolved in CH_2Cl_2 (5 mL) was added to this solution, which was stirred at room temperature for 1 h. The resulting mixture was concentrated in vacuo. The residue was purified by column chromatography.

Typical compound data are as follows：

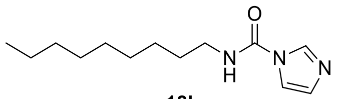

18b

***N*-Nonyl-1*H*-imidazole-1-carboxamide** (**18b**) [9] Yield: 72% (854 mg); white solid; R_f = 0.6 (EtOAc/PE, 2.5/1, v/v). ^1H NMR (400 MHz, $CDCl_3$, ppm):δ = 8.22 (s, 1H), 7.83 (t, *J* =

4.0 Hz, 1H), 7.51 (s, 1H), 6.98 (s, 1H), 3.35 (dd, J = 12.0Hz, 8.0Hz, 2H), 1.62 – 1.55 (m, 2H), 1.30 – 1.23 (m, 12H), 0.85 (t, J = 8.0 Hz, 3H).

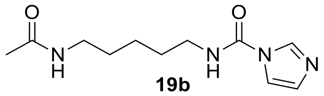

19b

***N*-(5-Acetamidopentyl)-1*H*-imidazole-1-carboxamide** (**19b**) Yield: 91% (1.08 g), white solid; Rf = 0.6 (EtOAc/PE, 2.5/1, v/v); ^{1}H NMR (400 MHz, $CDCl_3$, ppm): δ = 8.33 (s, 1H), 8.00 (br s, 1H), 7.65 (s, 1H), 7.01 (s, 1H), 6.21 (br s, 1H), 3.38 (dd, J = 12.0 Hz, 8.0 Hz, 2H), 3.24 (dd, J = 12.0 Hz, 4.0 Hz, 2H), 1.96 (s, 3H), 1.69 – 1.62 (m, 2H), 1.55 – 1.48 (m, 2H), 1.41 – 1.33 (m, 2H).

3. General procedure for the preparation of *N*-methylamines

To a stirred solution of *N*-substituted carbonylimidazoles (**b**) (2 mmol) and THF (20 mL) cooled to 0 °C in an ice bath, $NaBH_4$ (378 mg, 10.0 mmol) was added in one portion. Then a solution of I_2 (508 mg, 2 mmol) in THF (5 mL) was added slowly and drop-wise over 5 min resulting in vigorous evolution of hydrogen. Once gas evolution had ceased, the flask was heated to reflux for 1–10 h. The reaction was monitored by TLC. After completion of the reaction, the mixture was cooled to 0 °C with ice water, 6 M HCl was added carefully till pH = 1–2. After stirring for 1 h, NaOH (10% aq.) was added till pH = 12–13. The resulting solution was extracted with CH_2Cl_2 (3 × 50 mL). The organic extract was washed with brine, dried over anhydrous Na_2SO_4, filtered and concentrated under reduced pressure. The crude product was purified by column chromatography.

data of *N*-methylamines

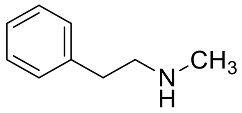

1c

***N*-Methyl-2-phenylethanamine (1c)** [10]

yellow liquid (200 mg, 74%); R_f: 0.4 ($MeOH/CH_2Cl_2$, 1/9, v/v). ^{1}H NMR (400 MHz, $CDCl_3$, ppm): δ = 7.31 – 7.26 (m, 2H), 7.21 – 7.20 (m, 3H), 2.86 – 2.78 (m, 4H), 2.42 (s, 3H). ^{13}C NMR (100 MHz, $CDCl_3$, ppm): δ = 139.94, 128.76, 128.51, 126.20, 53.07, 36.19, 36.03. ESI-MS: *m/z* calcd. for $C_9H_{14}N$ $[M+H]^+$ 136.1126; found 136.084.

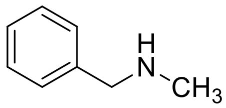

2c

***N*-Methylbenzylamine (2c)** [11]

yellow liquid (162 mg, 67%); R_f: 0.4 ($MeOH/CH_2Cl_2$, 1/9, v/v). ^{1}H NMR (400 MHz, $CDCl_3$, ppm): δ = 7.37 – 7.26 (m, 5H), 3.77 (s, 2H), 2.46 (s, 3H). ^{13}C NMR (100 MHz, $CDCl_3$, ppm): δ = 139.60, 128.45, 128.31, 127.12, 55.84, 35.74. ESI-MS: *m/z* calcd. for $C_8H_{12}N$ $[M+H]^+$ 122.0970; found 122.067.

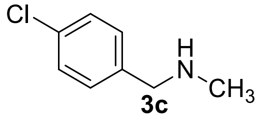

3c

***N*-Methyl-4-chlorobenzylamine (3c)** [12]

yellow liquid (208 mg, 67%); R_f: 0.4 ($MeOH/CH_2Cl_2$, 1/9, v/v). ^{1}H NMR (400 MHz, $CDCl_3$) δ = 7.27 (d, *J* = 8.0 Hz, 2H), 7.23 (d, *J* = 8.0 Hz, 2H), 3.69 (s, 2H), 2.41 (s, 3H). ^{13}C NMR

(100 MHz, $CDCl_3$, ppm): δ = 138.26, 132.74, 129.59, 128.52, 55.16, 35.79. ESI-MS: *m/z* calcd. for $C_8H_{11}ClN$ $[M+H]^+$ 156.0580 (Cl 35); found 156.028.

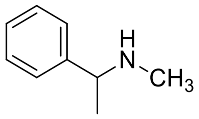

4c

***N*-Methyl-α-phenylethylamine (4c)** [13,14]

yellow liquid (176 mg, 65%); R_f: 0.4 ($MeOH/CH_2Cl_2$, 1/9, v/v). ^1H NMR (400 MHz, $CDCl_3$) δ = 7.38 – 7.35 (m, 4H), 7.32 – 7.27 (m, 1H), 3.99 (br s, 1H), 3.78 (q, *J* = 6.7 Hz, 1H), 2.35 (s, 3H), 1.49 (d, *J* = 6.7 Hz, 3H). ^{13}C NMR (100 MHz, $CDCl_3$, ppm) δ = 142.77, 128.70, 127.59, 126.92, 60.08, 33.44, 22.83. ESI-MS: *m/z* calcd. for $C_9H_{14}N$ $[M+H]^+$ 136.1126; found 136.1091.

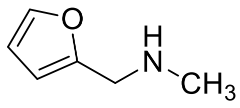

5c

***N*-Methylfurfurylamine (5c)** [15]

yellow liquid (155 mg, 70%); R_f: 0.4 ($MeOH/CH_2Cl_2$, 1/9, v/v). ^1H NMR (400 MHz, $CDCl_3$, ppm): δ = 7.34 (d, *J* = 1.2 Hz, 1H), 6.29 (dd, *J* = 2.8 Hz, 2.0, 1H), 6.16 (d, *J* = 2.8 Hz, 1H), 3.71 (s, 2H), 2.39 (s, 3H). ^{13}C NMR (100 MHz, $CDCl_3$, ppm): δ = 153.50, 141.86, 110.10, 107.12, 47.87, 35.51. ESI-MS: *m/z* calcd. for $C_6H_{10}NO$ $[M+H]^+$ 112.0757; found 112.052.

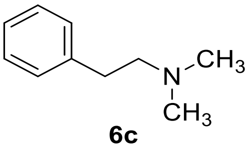

6c

***N*,*N*-Dimethyl-2-phenylethanamine (6c)** [16]

yellow liquid (247 mg, 83%); R*f*: 0.4 (MeOH/CH_2Cl_2, 1/9, v/v). ^{1}H NMR (400 MHz, $CDCl_3$, ppm): δ = 7.32 – 7.26 (m, 2H), 7.23 – 7.18 (m, 3H), 2.80 (t, *J* = 8.0 Hz, 2H), 2.54 (t, *J* = 8.0 Hz, 2H), 2.31 (s, 6H). ^{13}C NMR (100 MHz, $CDCl_3$, ppm): δ = 140.40, 128.68, 128.42, 126.04, 61.65, 45.52, 34.45. ESI-MS: *m/z* calcd. for $C_{10}H_{16}N$ $[M+H]^+$ 150.1283; found 150.099.

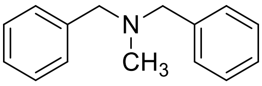

7c

***N*-Methyldibenzylamine (7c)** [17]

yellow liquid (253 mg, 60%); R*f*: 0.4 (MeOH/CH_2Cl_2, 1/9, v/v). ^{1}H NMR (400 MHz, $CDCl_3$, ppm): δ = 7.44 – 7.36 (m, 8H), 7.33 – 7.29 (m, 2H), 3.59 (s, 4H), 2.25 (s, 3H). ^{13}C NMR (100 MHz, $CDCl_3$, ppm): δ = 132.90, 131.42, 128.99, 128.14, 66.41, 45.97. ESI-MS: *m/z* calcd. for $C_{15}H_{18}N$ $[M+H]^+$ 212.1439; found 212.121.

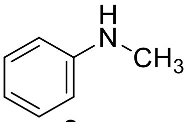

8c

***N*-Methylaniline (8c)** [18]

yellow liquid (154 mg, 72%); R*f*: 0.6 (EA/PE, 1/4, v/v). ^{1}H NMR (400 MHz, $CDCl_3$) δ = 7.26 (dd, *J* = 8.0 Hz, 7.2 Hz, 2H), 6.78 (t, *J* = 7.2 Hz, 1H), 6.67 (d, *J* = 7.6 Hz, 2H), 3.56 (br s, 1H), 2.87 (s, 3H). ^{13}C NMR (100 MHz, $CDCl_3$, ppm): δ = 149.38, 129.29, 117.33, 112.52, 30.81. ESI-MS: *m/z* calcd. for $C_7H_{10}N$ $[M+H]^+$ 108.0813; found 108.0789.

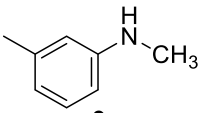

9c

***N,3*-Dimethylbenzenamine (9c)** [18,19]

yellow liquid (206 mg, 85%); R*f*: 0.6 (EA/PE, 1/4, v/v). ^{1}H NMR (400 MHz, $CDCl_3$) δ = 7.18 (t, *J* = 8.0 Hz, 1H), 6.64 (d, *J* = 8.0 Hz, 1H), 6.52 – 6.51 (m, 2H), 3.56 (br s, 1H), 2.89 (s, 3H), 2.39 (s, 3H). ^{13}C NMR (100 MHz, $CDCl_3$, ppm): δ = 149.51, 139.05, 129.18, 118.28, 113.28, 109.73, 30.86, 21.76. ESI-MS: *m/z* calcd. for $C_8H_{12}N$ $[M+H]^+$ 122.0970; found 122.0951.

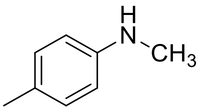

10c

***N*,4-Dimethylbenzenamine (10c)** [18]

yellow liquid (194 mg, 80%); R*f*: 0.6 (EA/PE, 1/4, v/v). ^{1}H NMR (400 MHz, $CDCl_3$, ppm) δ = 7.04 (d, *J* = 8.0 Hz, 2H), 6.58 (d, *J* = 8.0 Hz, 2H), 3.23 (br s, 1H), 2.84 (s, 3H), 2.28 (s, 3H).^{13}C NMR (100 MHz, $CDCl_3$, ppm): δ = 147.25, 129.79, 126.51, 112.71, 31.17, 20.49. ESI-MS: *m/z* calcd. for $C_8H_{12}N$ $[M+H]^+$ 122.0970; found 122.069.

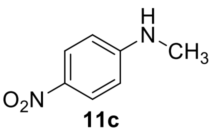

11c

***N*-Methyl-4-nitrobenzenamine (11c)** [18]

yellow liquid (213 mg, 70%); R_f: 0.6 (EA/PE, 1/4, v/v). ^1H NMR (400 MHz, $CDCl_3$, ppm): δ = 8.08 (d, J = 8.0 Hz, 2H), 6.52 (d, J = 8.0 Hz, 2H), 4.67 (br s, 1H), 2.93 (d, J = 4.7 Hz, 3H). ^{13}C NMR (100 MHz, $CDCl_3$, ppm): δ = 154.25, 137.90, 126.42, 110.71, 30.15. ESI-MS: m/z calcd. for $C_7H_9N_2O_2$ $[M+H]^+$ 153.0664; found 153.0631.

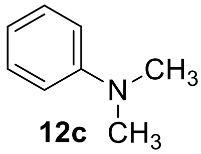

12c

***N*,*N*-Dimethylaniline (12c)** [17]

yellow liquid (172 mg, 71%); R_f: 0.6 (EA/PE, 1/4, v/v). ^1H NMR (400 MHz, $CDCl_3$, ppm): δ = 7.29 – 7.25 (m, 2H), 6.79-6.74 (m, 3H), 2.97 (s, 6H). ^{13}C NMR (100 MHz, $CDCl_3$, ppm): δ = 150.61, 129.11, 116.76, 112.75, 40.71. ESI-MS: m/z calcd. for $C_8H_{12}N$ $[M+H]^+$ 122.0970; found 122.0961.

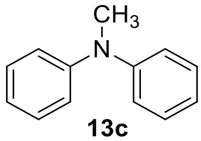

13c

***N*-Methyldiphenylamine (13c)** [17]

yellow liquid (245 mg, 67%); R_f: 0.6 (EA/PE, 1/4, v/v). ^1H NMR (400 MHz, $CDCl_3$) δ = 7.30 – 7.26 (m, 4H), 7.04 (d, J = 8.0 Hz, 4H), 6.97 (t, J = 8.0 Hz, 2H), 3.33 (s, 3H). ^{13}C NMR (100 MHz, $CDCl_3$, ppm): δ = 148.92, 129.23, 121.41, 120.48, 40.36. ESI-MS: m/z calcd. for $C_{13}H_{14}N$ $[M+H]^+$ 184.1126; found 184.1098.

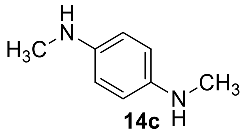

14c

***N,N'*-Dimethyl-1,4-benzenediamine (14c)** [20]

green solid (191 mg, 70%); R$_f$: 0.6 (EA/PE, 2.5/1, v/v). ^{1}H NMR (400 MHz, $CDCl_3$, ppm): δ = 6.60 (s, 4H), 3.36 (br s, 2H), 2.80 (s, 6H). ^{13}C NMR (100 MHz, $CDCl_3$, ppm): δ = 141.89, 114.31, 31.95. ESI-MS: *m/z* calcd. for $C_8H_{13}N_2$ $[M+H]^+$ 137.1079; found 137.078.

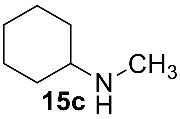

15c

***N*-Methylcyclohexanamine (15c)** [13,14,21-23]

colorless liquid (167 mg, 74%); R$_f$: 0.4 (MeOH/CH_2Cl_2, 1/9, v/v). ^{1}H NMR (400 MHz, $CDCl_3$, ppm) δ = 3.68 (br s, 1H), 2.76 – 2.69 (m, 1H), 2.48 (d, *J* = 8.0 Hz, 3H), 2.06 (d, *J* = 12.0 Hz, 1H), 1.91 (d, *J* = 12.0 Hz, 1H), 1.81 (d, *J* = 12.9 Hz, 2H), 1.68 – 1.62 (m, 1H), 1.48 – 1.44 (m, 1H), 1.33 – 1.23 (m, 3H), 1.16 – 1.09 (m, 1H). ^{13}C NMR (100 MHz, $CDCl_3$, ppm): δ = 63.12, 38.05, 29.46, 27.90, 25.49, 25.19, 25.17. ESI-MS: *m/z* calcd. for $C_7H_{16}N$ $[M+H]^+$ 114.1283; found 114.1254.

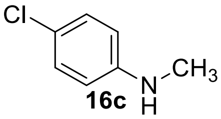

16c

4-Chloro-*N*-methylbenzenamine (16c) [18,24]

yellow liquid (209 mg, 74%); R$_f$: 0.6 (EA/PE, 1/4, v/v). ^{1}H NMR (400 MHz, $CDCl_3$, ppm): δ = 7.14 (d, *J* = 8.0 Hz, 2H), 6.53 (d, *J* = 12.0 Hz, 2H), 3.68 (br s, 1H), 2.81 (s, 3H). ^{13}C NMR (100 MHz, $CDCl_3$, ppm): δ = 152.77, 129.02, 121.73, 113.45, 30.82. ESI-MS: *m/z* calcd. for C_7H_8ClN $[M+H]^+$ 142.0424 (Cl 35); found 142.0410.

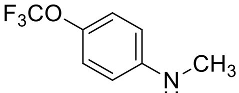

17c

***N*-Methyl-4-(trifluoromethoxy)benzenamine (17c)** [24]

yellow liquid (275 mg, 72%); R*f*: 0.6 (EA/PE, 1/4, v/v). ^{1}H NMR (400 MHz, $CDCl_3$, ppm): δ = 7.07 (d, *J* = 8.0 Hz, 2H), 6.56 (d, *J* = 12.0 Hz, 2H), 3.68 (br s, 1H), 2.83 (s, 3H). ^{13}C NMR (100 MHz, $CDCl_3$, ppm): δ = 148.11, 140.34, 122.40, 120.79 (q, *J* = 255.1 Hz, CF_3), 112.54 30.79. ESI-MS: *m/z* calcd. for $C_8H_9F_3NO$ $[M+H]^+$ 192.0636; found 192.0613.

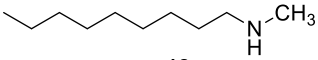

18c

***N*-Methylnonylamine (18c)** [25]

yellow liquid (242 mg, 77%); R*f*: 0.4 (MeOH/CH_2Cl_2, 1/9, v/v). ^{1}H NMR (400 MHz, $CDCl_3$, ppm): δ = 2.58 – 2.54 (m, 3H), 2.42 (s, 3H), 1.50 – 1.47 (m, 2H), 1.26 – 1.24 (m, 12H), 0.86 (t, *J* = 6.8 Hz, 3H). ^{13}C NMR (100 MHz, $CDCl_3$, ppm): δ = 51.91, 36.12, 31.88, 29.55, 29.55, 29.52, 29.28, 27.30, 22.67, 14.11. ESI-MS: *m/z* calcd. for $C_{10}H_{24}N$ $[M+H]^+$ 158.1909; found 158.163.

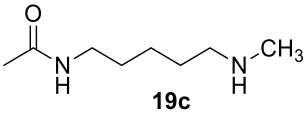

19c

***N*-[5-(Methylamino)pentyl]acetamide (19c)** [26]

yellow liquid (206 mg, 65%); R*f*: 0.4 (MeOH/CH_2Cl_2, 1/9, v/v). ^{1}H NMR (400 MHz, $CDCl_3$, ppm): δ = 6.11 (br s, 1H), 3.18 (dd, *J* = 4.0, 12.0 Hz, 2H), 2.53 (t, *J* = 8.0 Hz, 2H), 2.38 (s, 3H), 2.31 (br s, 1H), 1.92 (s, 3H), 1.51 – 1.43 (m, 4H), 1.36 – 1.28 (m, 2H). ^{13}C NMR (100

MHz, $CDCl_3$, ppm): δ = 170.25, 51.63, 39.47, 36.27, 29.31, 29.20, 24.50, 23.26. ESI-MS: *m/z* calcd. for $C_8H_{19}N_2O$ $[M+H]^+$ 159.1497; found 159.1514.

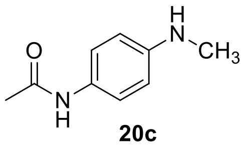

20c

***N*-[4-(Methylamino)phenyl]acetamide (20c)** [27]

green solid (223 mg, 68%); R_f: 0.4 (EA/PE, 2.5/1, v/v). ^1H NMR (400 MHz, $CDCl_3$, ppm): δ = 7.80 (br s, 1H), 7.25 (d, *J* = 8.0 Hz, 2H), 6.52 (d, *J* = 8.0 Hz, 2H), 3.45 (br s, 1H), 2.77 (s, 3H), 2.07 (s, 3H). ^{13}C NMR (100 MHz, $CDCl_3$, ppm): δ = 168.66, 146.50, 128.26, 122.49, 112.58, 31.04, 24.13. ESI-MS: *m/z* calcd. for $C_9H_{13}N_2O$ $[M+H]^+$ 165.1028; found 165.073.

4. Data of the two intermediates

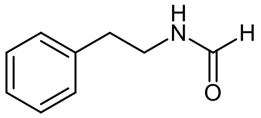

***N*-(2-Phenylethyl)formamide**

^{1}H NMR (400 MHz, $CDCl_3$, ppm): δ = 8.10 (s, 1H), 7.35 – 7.21 (m, 5H), 6.01 (br s, 1H), 3.57 (dd, *J* = 13.6 Hz, 6.8 Hz, 2H), 2.85 (t, *J* = 6.8 Hz, 2H). ^{13}C NMR (100 MHz, $CDCl_3$, ppm): δ = 161.37, 138.54, 128.78, 128.71, 126.66, 39.24, 35.49.

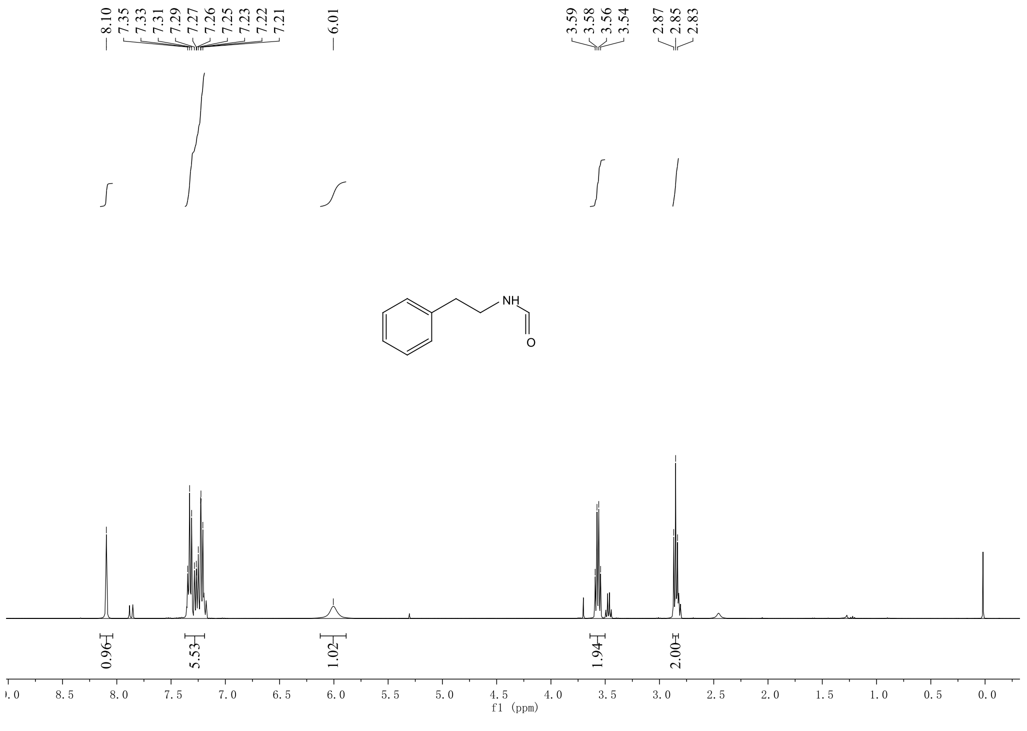

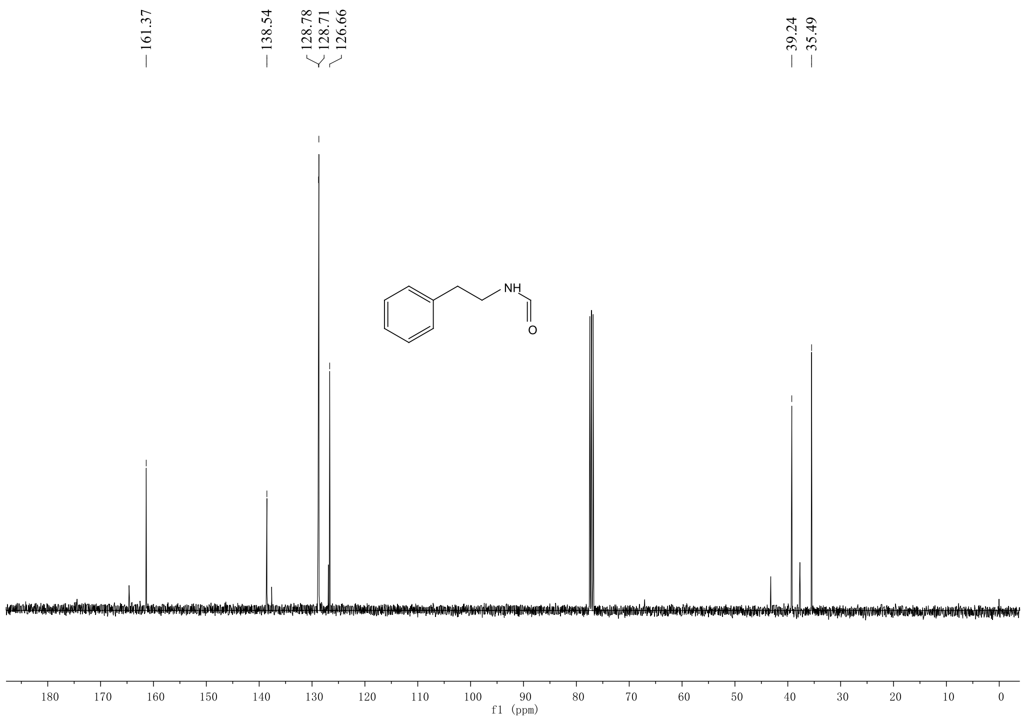

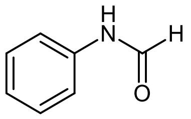

***N*-Phenylformamide** [6,28]

^{1}H NMR (400 MHz, $CDCl_3$, ppm) (*trans/cis*=54/46): δ = 8.87 (br s, 1H, *trans*), 8.70 (d, *J* = 11.4 Hz, 1H, *trans*), 8.35 (d, *J* = 1.8 Hz, 1H, *cis*), 7.96 (br s, 1H, *cis*), 7.55 (d, *J* = 7.7 Hz, 1H), 7.37 – 7.30 (m, 4H), 7.20 – 7.10 (m, 4H). ^{13}C NMR (100 MHz, $CDCl_3$, ppm): δ = 163.04, 159.45, 136.98, 136.80, 129.77, 129.12, 125.31, 124.83, 120.09, 118.81.

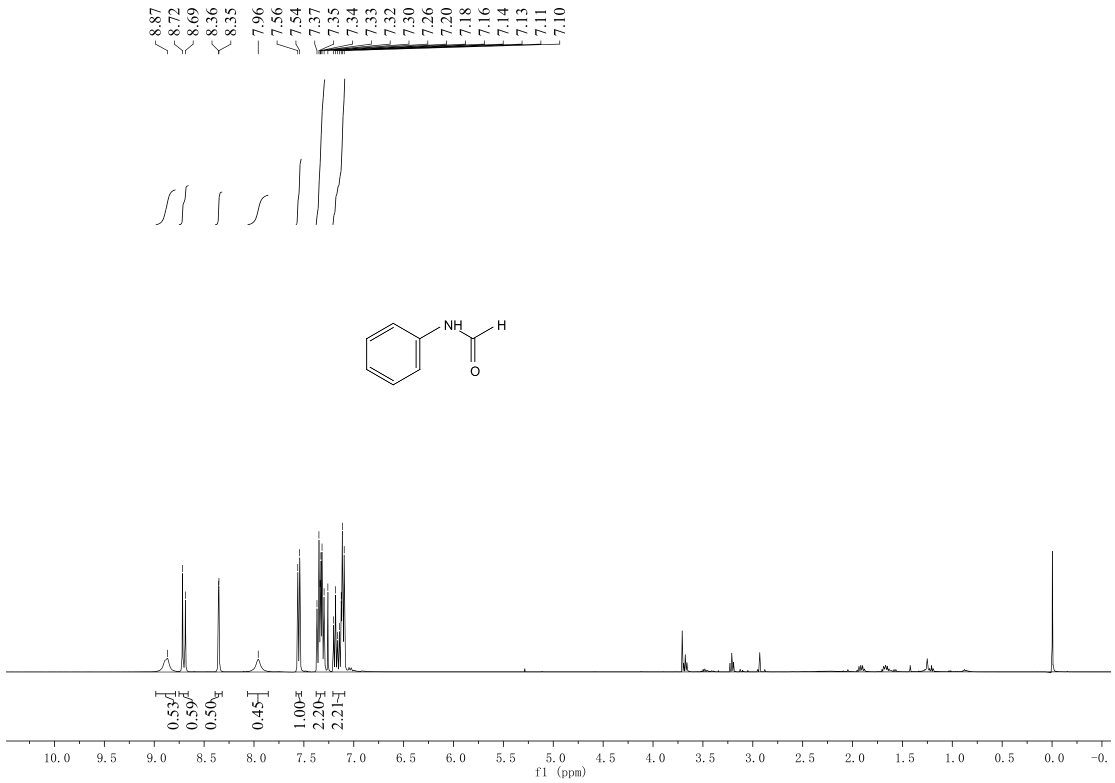
8.87
8.72
8.69
8.36
8.35
7.96
7.56
7.54
7.37
7.35
7.34
7.33
7.32
7.30
7.26
7.20
7.18
7.16
7.14
7.13
7.11
7.10
NH
H
O
0.53
0.59
0.50
0.45
1.00
2.20
2.21
10.0 9.5 9.0 8.5 8.0 7.5 7.0 6.5 6.0 5.5 5.0 4.5 4.0 3.5 3.0 2.5 2.0 1.5 1.0 0.5 0.0 -0.
f1 (ppm)

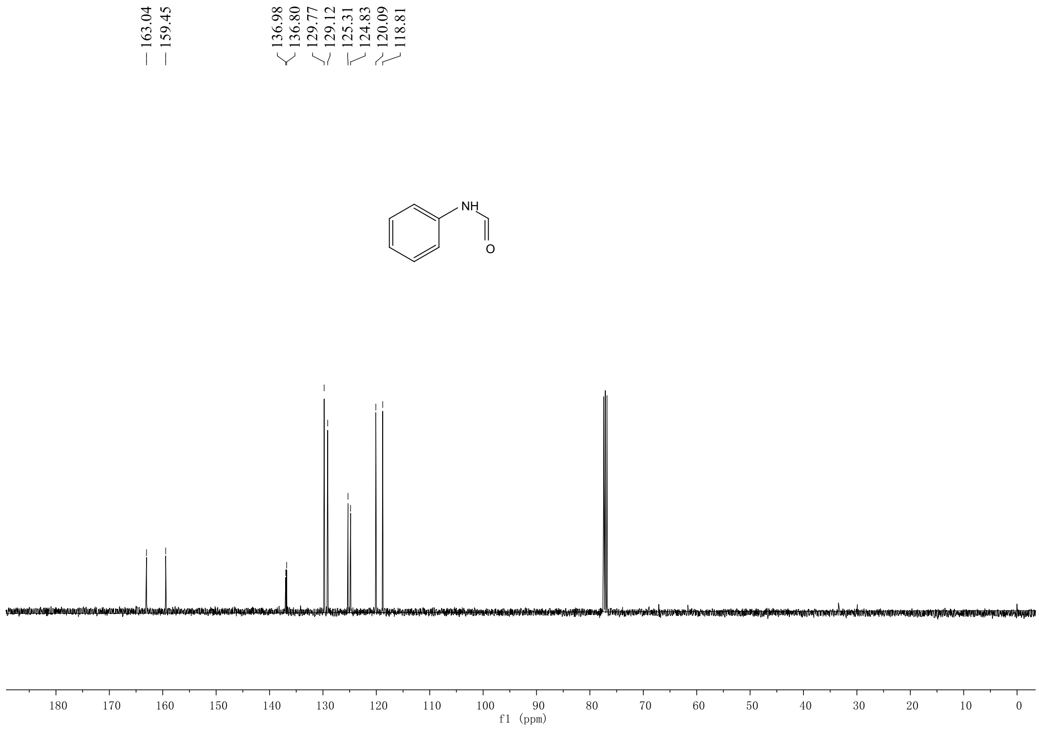
163.04
159.45
136.98
136.80
129.77
129.12
125.31
124.83
120.09
118.81
NH
O
180 170 160 150 140 130 120 110 100 90 80 70 60 50 40 30 20 10 0
f1 (ppm)

5. Scheme S1

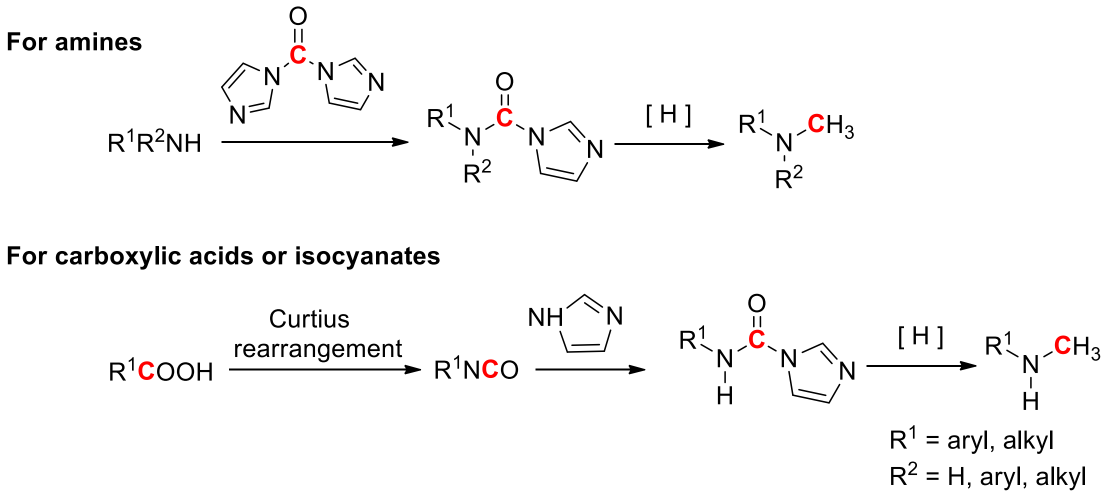

The carbon source was marked in red.

Scheme S1: Schematic of the conversion process for N-CH_3 carbon source.

6. References

1. Velavan, A.; Sumathi, S.; Balasubramanian, K. K. *Org. Biomol. Chem.* **2012,** *10,* 6420–6431.
2. Padiya, K. J.; Gavade, S.; Kardile, B.; Tiwari, M.; Bajare, S.; Mane, M.; Gaware, V.; Varghese, S.; Harel, D.; Kurhade, S. *Org. Lett.* **2012,** *14,* 2814–2817.
3. Rawling, T.; McDonagh, A. M.; Tattam, B.; Murray, M. *Tetrahedron* **2012,** *68,* 6065–6070.
4. McBurney, R. T.; Walton, J. C. *J. Am. Chem. Soc.* **2013,** *135,* 7349–7354.
5. Perboni, A.; Pentassuglia, G.; Andreotti, D.; Winders, John A.; Urea derivatives of tricyclic carbapenems as bactericides. PCT Int. Appl. WO 9513278 A1, May 18, 1995.
6. Chen, Z. Y.; Cao, Y. M.; Tian, Z. Y.; Zhou, X.; Xu, W. J.; Yang, J.; Teng. H. B. *Tetrahedron Lett.* **2017,** *58,* 2166–2170.
7. Teng, H. B.; Zhang, Z. W.; Zhou, Y. F.; Chen, Z. Y.; Chen, Q.; Liu, Y.; Xu, W. J. *RSC Adv.* **2015,** *5,* 71868–71872.
8. Jöst, C.; Nitsche, C.; Scholz, T.; Roux, L.; Klein, C. D. *J. Med. Chem.* **2014,** *57,* 7590–7599.
9. Alsarraf, J.; Petitpoisson, L.; Pichette, A. *Org. Lett.* **2021,** *23 (15),* 6052-6056.
10. Huang, Z.; Lv, J.; Jia, Y. *ChemistrySelect*. **2016,** *1,* 5892–5894.
11. Petricci, E.; Santillo, N.; Castagnolo, D.; Cini, E.; Taddei, M. *Adv. Synth. Catal.* **2018,** *360,* 2560–2565.
12. Zishiri, V. K.; Hunter, R.; Smith, P. J.; Taylor, D.; Summers, R.; Kirk, K.; Martin, R. E.; Egan, T. J. *Eur. J. Med. Chem.* **2011,** *46,* 1729–1742.
13. Jaźwiński, J. *Journal of Molecular Structure* **2005,** *750,* 7–17.
14. Ji, P.; Manna, K.; Lin, Z.; Feng, X.; Urban, A.; Song, Y.; Lin, W. *J. Am. Chem. Soc.* **2017,** *139,* 7004−7011.
15. Jiang, Z. G.; Wang, Y.; Wang, W. Y.; Wang, S. Z.; Xu, B.; Fan, G. R.; Dong, G. Q.; Liu, Y.; Yao, J. Z.; Miao, Z. Y.; Zhang, W. N.; Sheng, C. Q. *Eur. J. Med. Chem.* **2013,** *64,* 16-22.
16. Choi, G.; Hong, S. H. *ACS Sustain. Chem. Eng.* **2019,** *7 (1),* 716-723.
17. Guo, Z.; Zhang, B.; Wei, X.; Xi, C. *ChemSusChem* **2018,** *11,* 2296–2299.
18. Sun, N.; Wang, S.; Mo, W.; Hu, B.; Shen, Z.; Hu, X. *Tetrahedron* **2010,** *66,* 7142–7148.
19. González, I.; Mosquera, J.; Guerrero, C.; Rodríguez, R.; & Cruces, *J. Org. Lett.* **2009,** *18*, 1677–1680.
20. Lewis, F. D.; Kurth, T. L.; Delos Santos, G. B. *J. Phys. Chem. B* **2005,** *109,* 4893–4899.
21. France, S. P.; Howard, R. M.; Steflik, J.; Weise, N. J.; Mangas-Sanchez, J.; Montgomery, S. L.; Crook, R.; Kumar, R.; Turner, N. J. *ChemCatChem* **2018,** *10*, 510–514.
22. Kundu, S. K.; Mitra, K.; Majee, A. *RSC Adv.* **2013,** *3,* 8649–8651.
23. Huang, Z.; Lv, J.; Jia, Y. *ChemistrySelect* **2016,** *1,* 5892–5894.
24. Chen, J.; Wu, J.; Tu, T. *ACS Sustainable Chem. Eng.* **2017,** *5,* 11744−11751.
25. Cohen, T. *J.* Org. *Chem.* **1983,** *48,* 4531-4537.

26. Yamauchi, F.; Hamachi, I.; Sakamoto, T.; Fluorine-containing compound and method for detecting biomolecule using the same. PCT Int. Appl. WO 2009113704 A2, Sep 17, 2009.
27. Ikawa, T.; Barder, T. E.; Biscoe, M. R.; Buchwald, S. L. *J. Am. Chem. Soc.* **2007,** *129,* 13001–13007.
28. Bhanage, B.; Nale, D. *Synlett* **2016,** *27,* 1413–1417.

7. ^{1}H and ^{13}C NMR spectra of b and c

N-Phenethyl-1*H*-imidazole-1-carboxamide (1b)

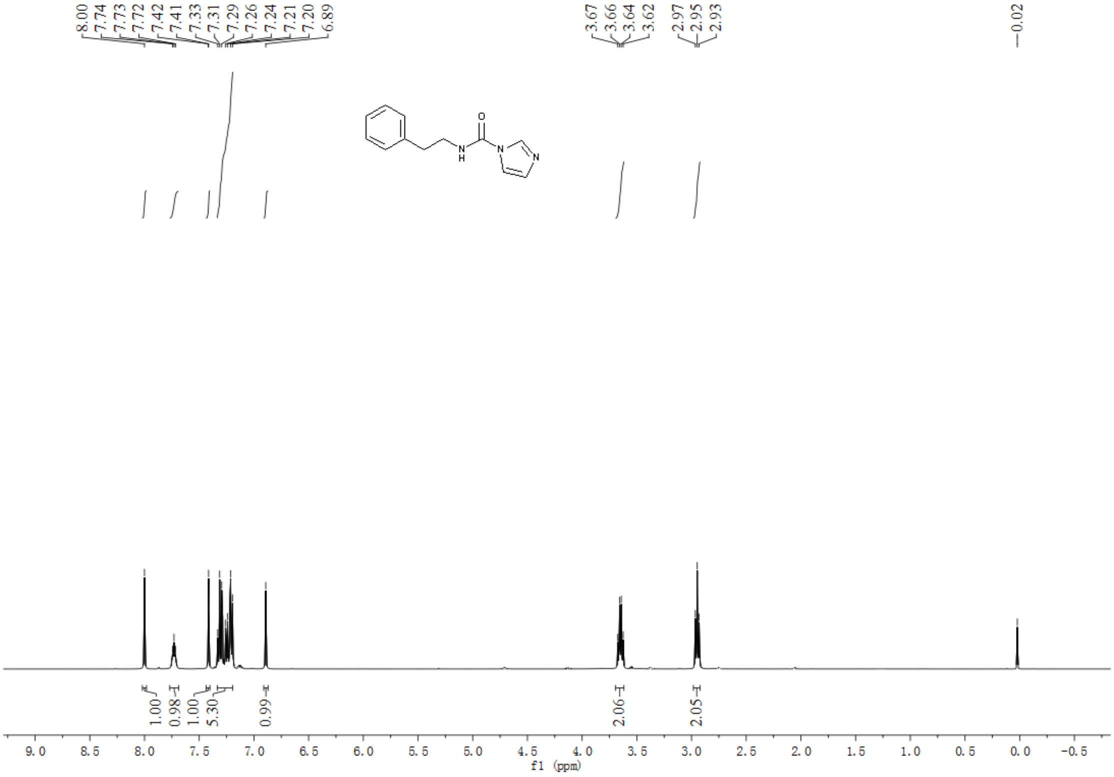

N-(Furan-2-ylmethyl)-1*H*-imidazole-1-carboxamide (5b)

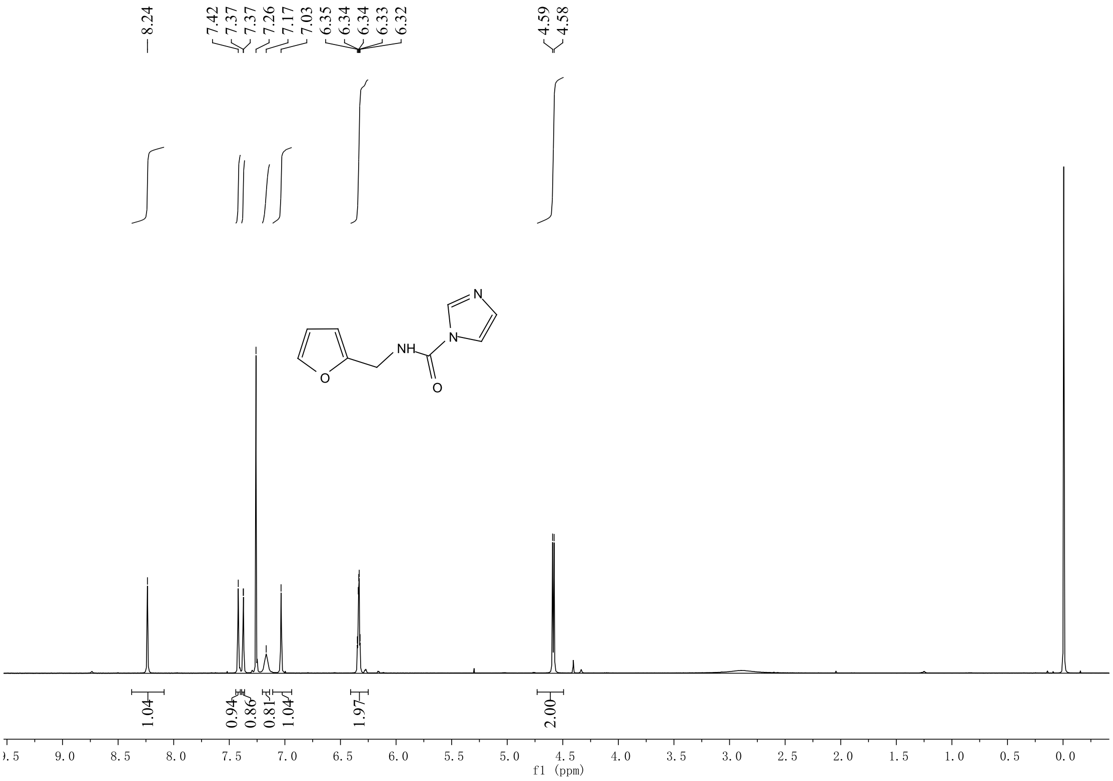

***N*,*N*-Dibenzyl-1*H*-imidazole-1-carboxamide (7b)**

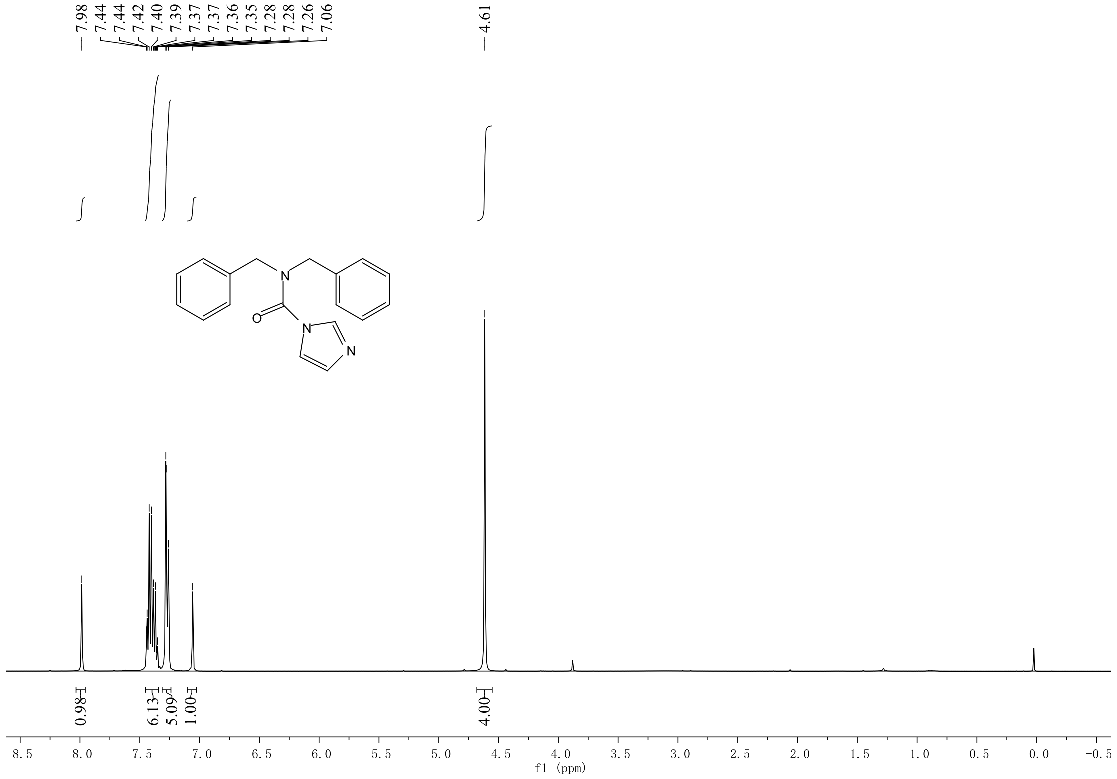

***N*-Methyl-*N*-phenyl-1*H*-imidazole-1-carboxamide (12b)**

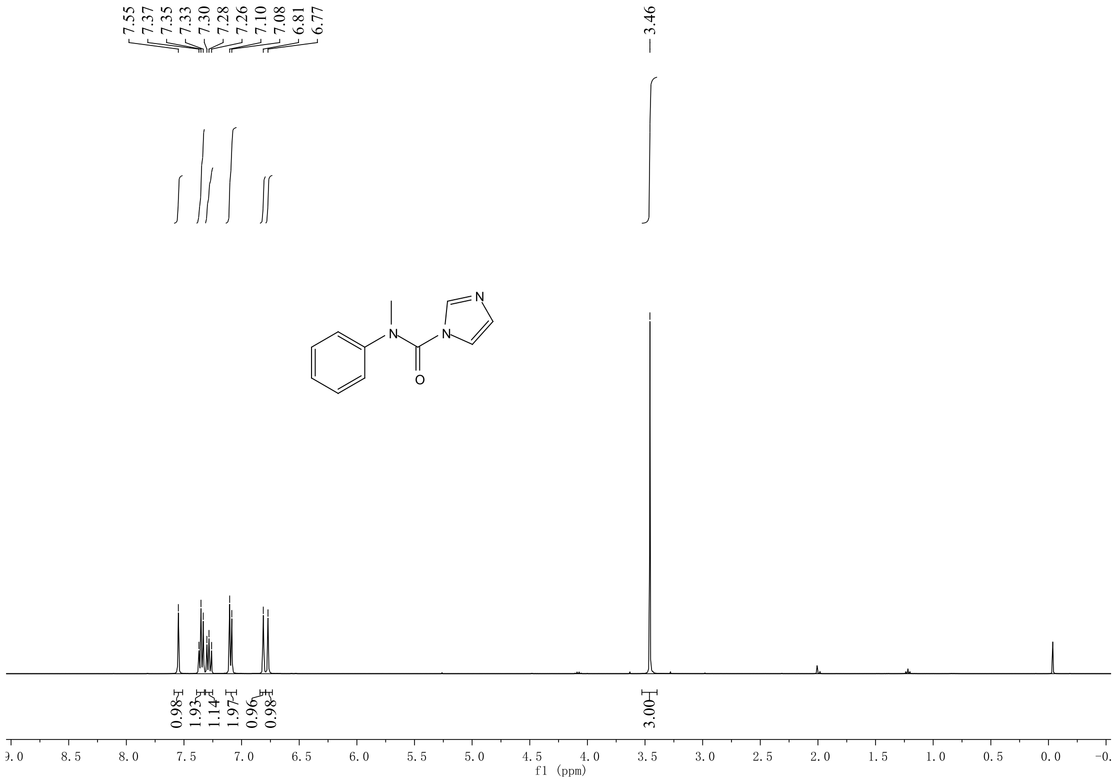

***N*,*N*-Diphenyl-1*H*-imidazole-1-carboxamide (13b)**

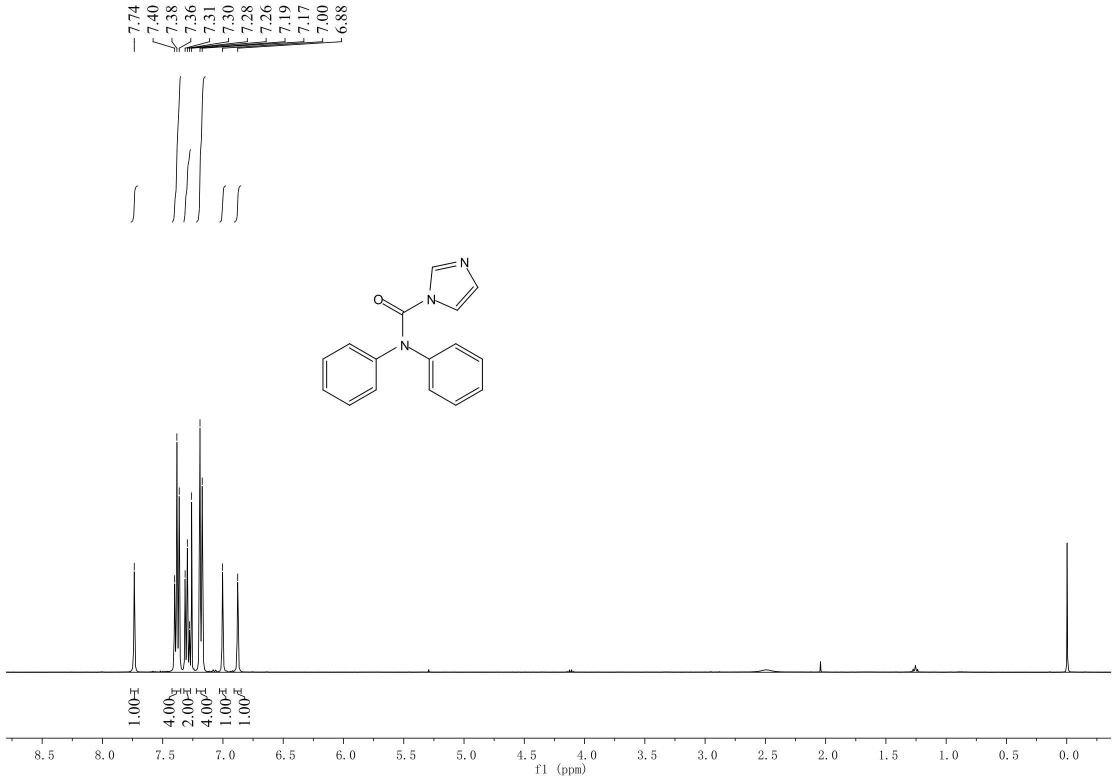

***N*-Nonyl-1*H*-imidazole-1-carboxamide (18b)**

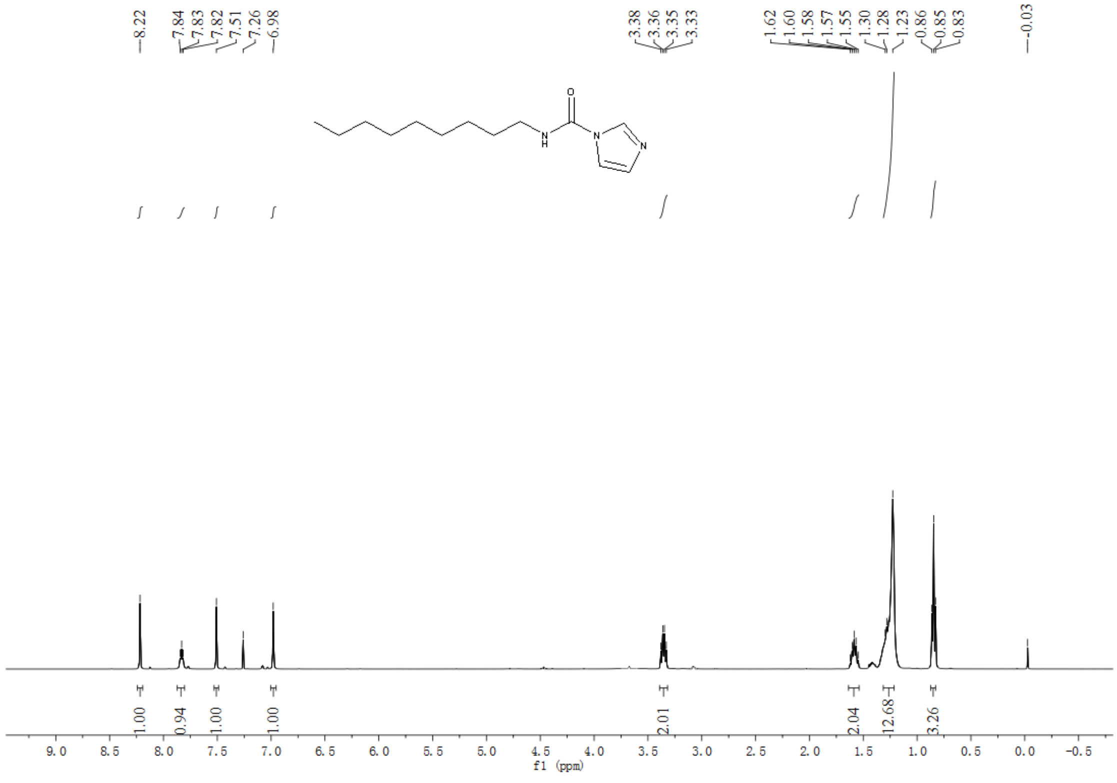

***N*-(5-Acetamidopentyl)-1*H*-imidazole-1-carboxamide (19b)**

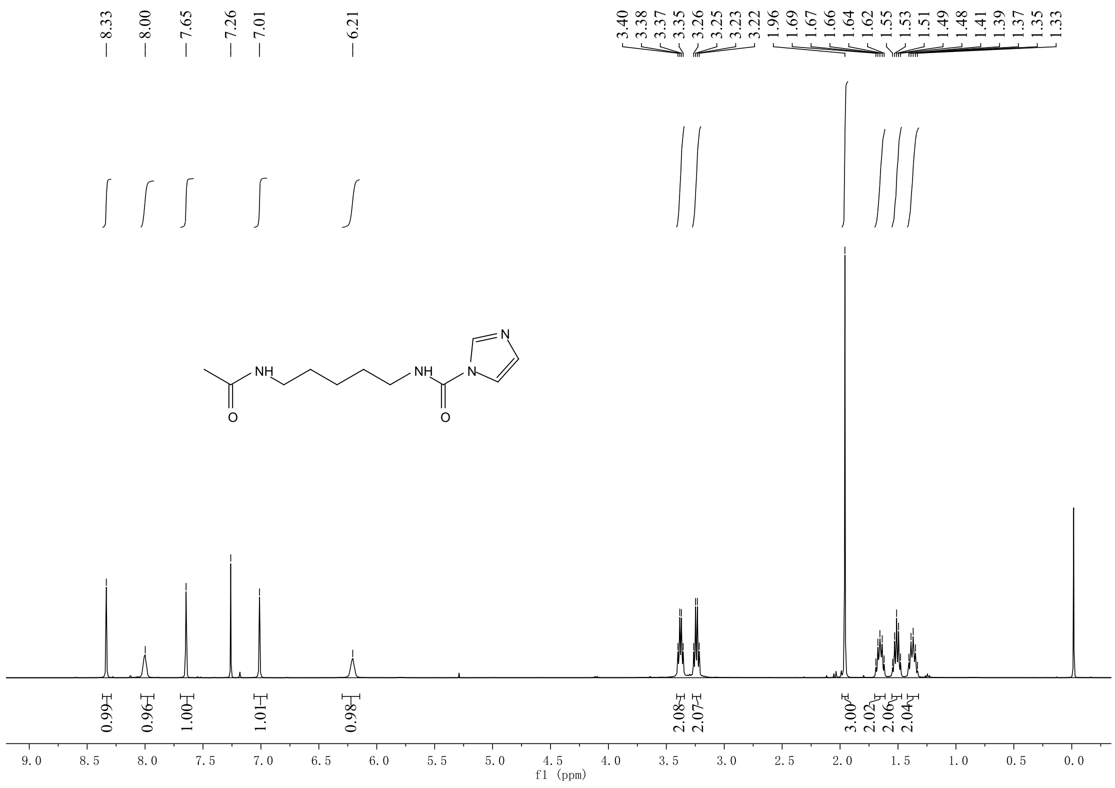

***N*-Methyl-2-phenylethanamine (1c)**

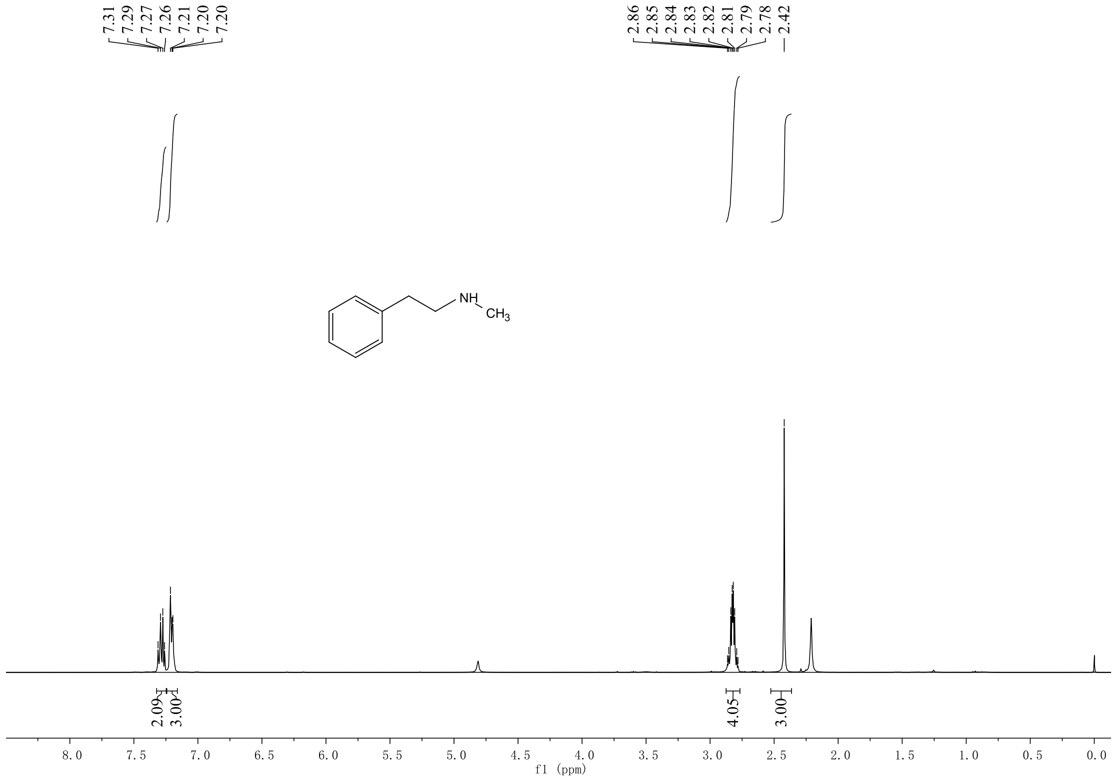

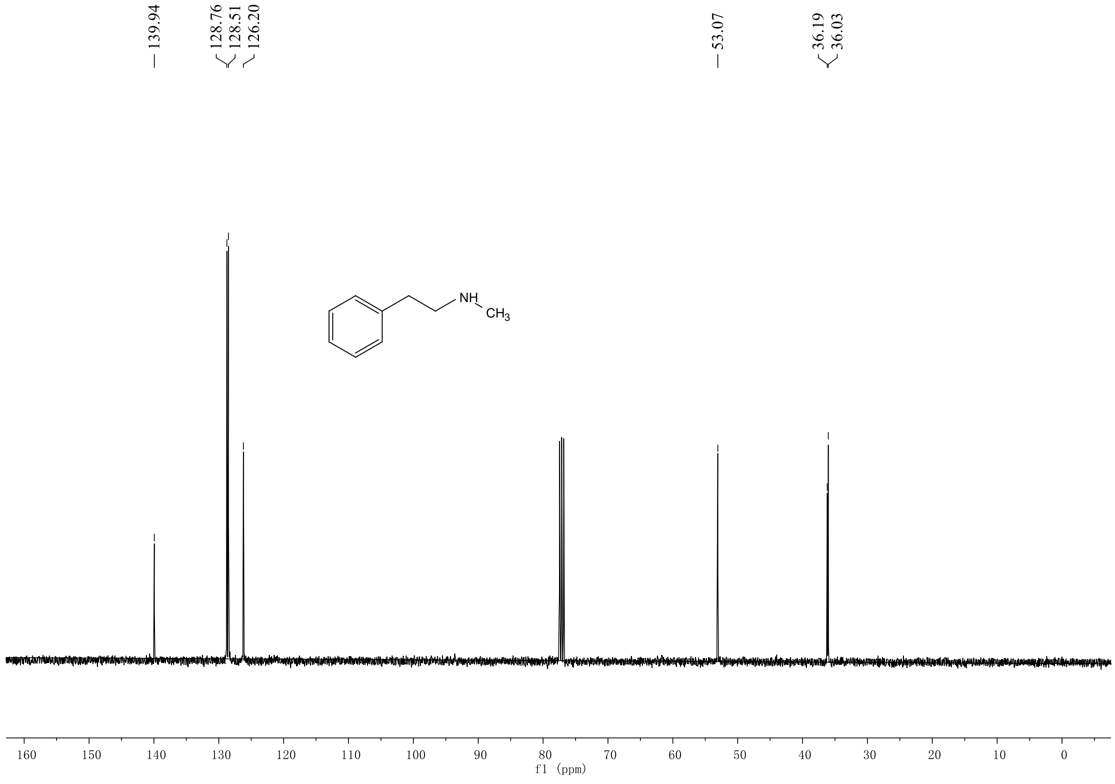

***N*-Methyl-1-phenylmethanamine (2c)**

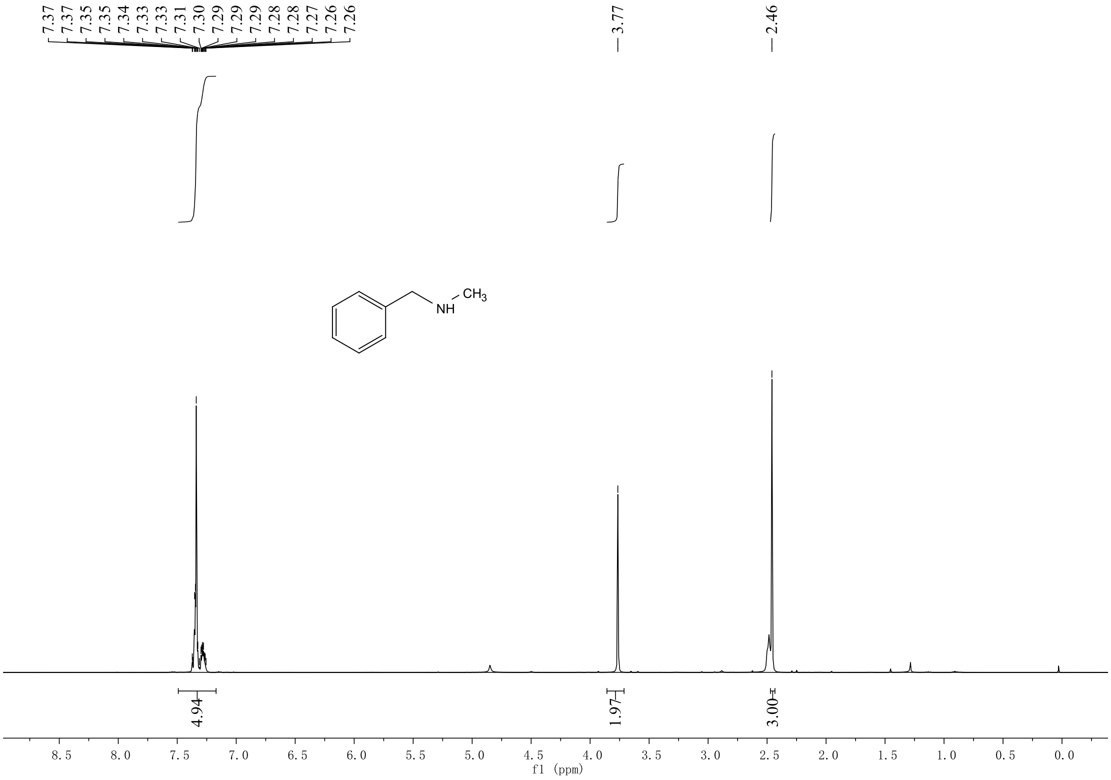

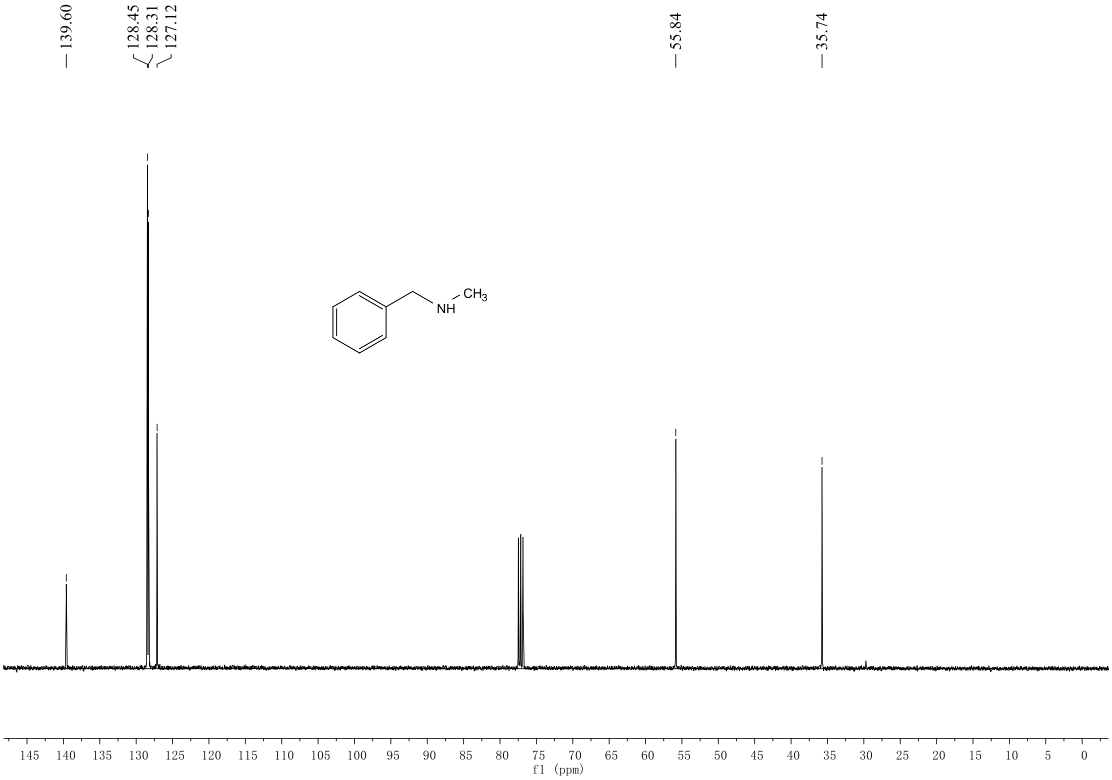

1-(4-Chlorophenyl)-*N*-methylmethanamine (3c)

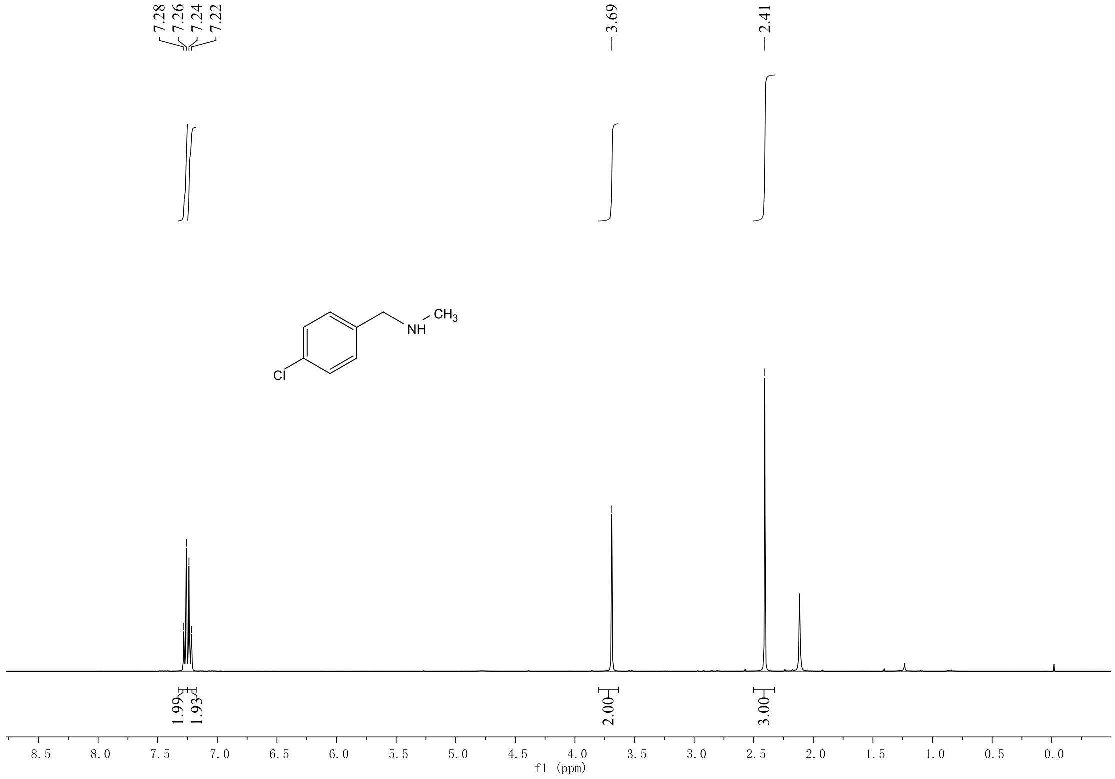

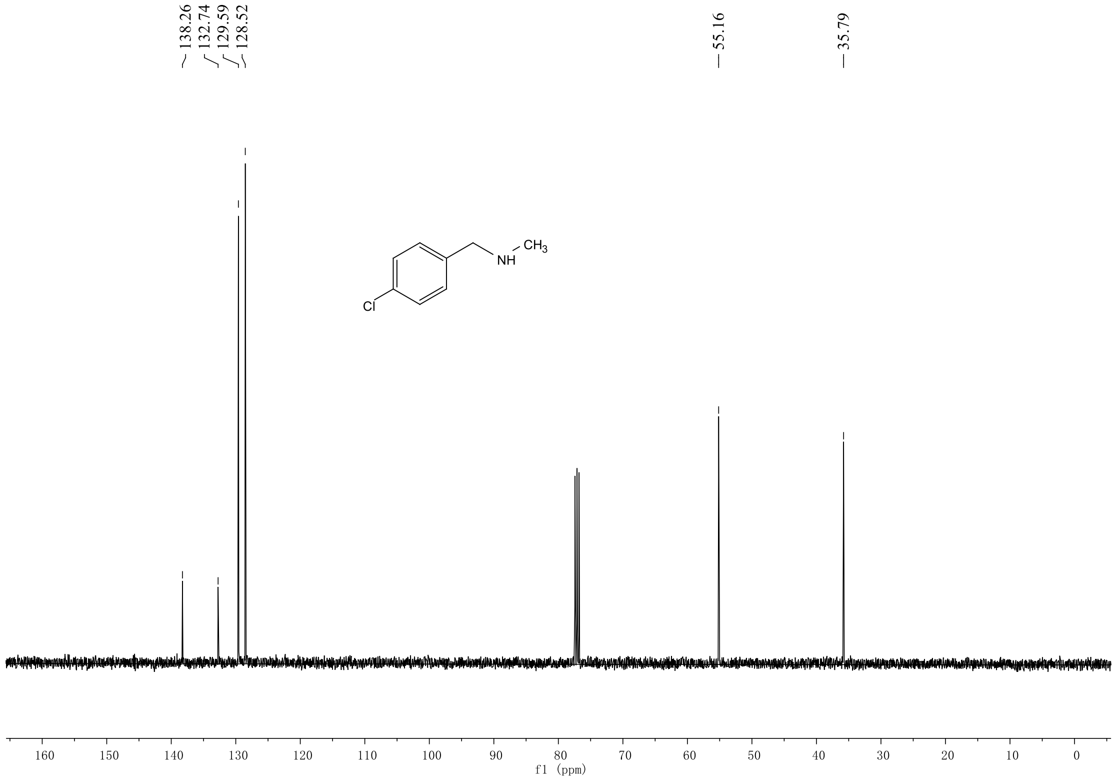

***N*-Methyl-1-phenylethanamine (4c)**

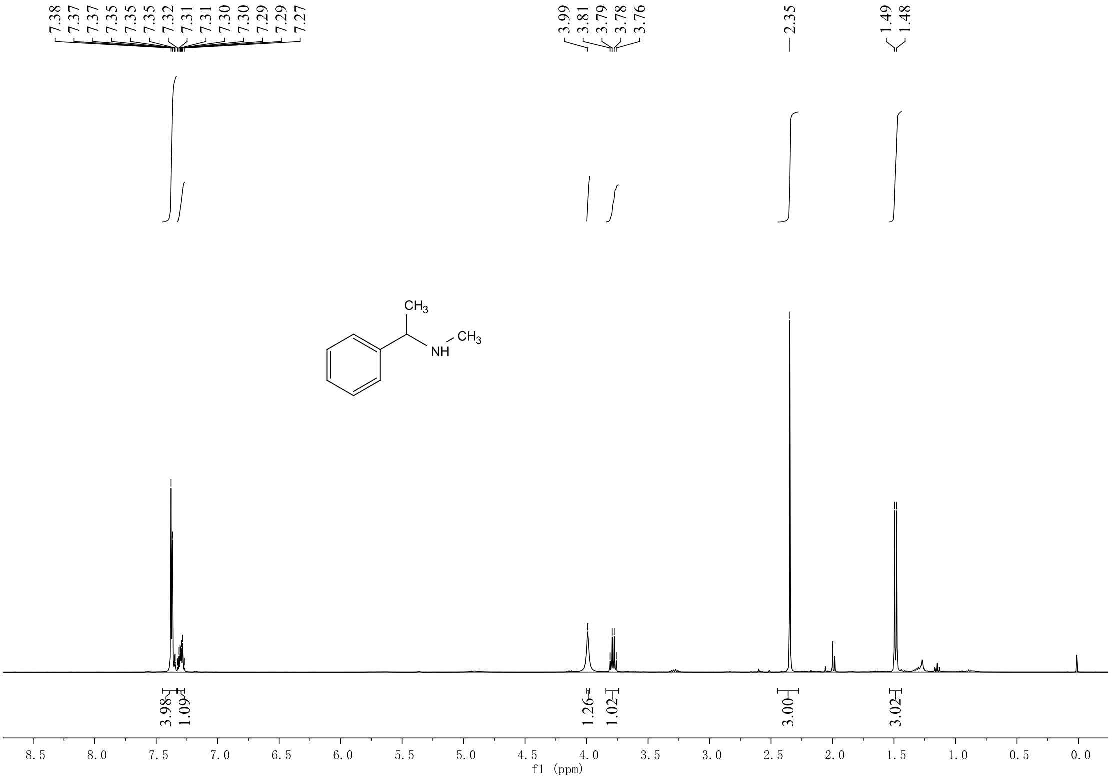

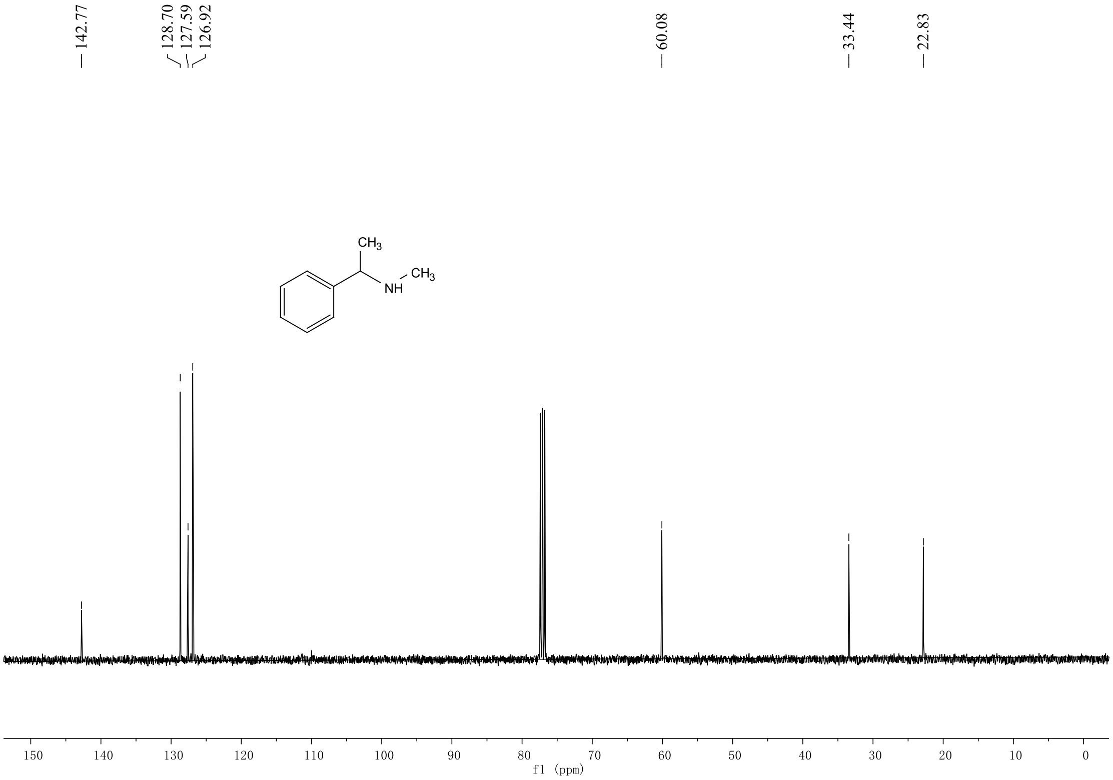

1-(Furan-2-yl)-*N*-methylmethanamine (5c)

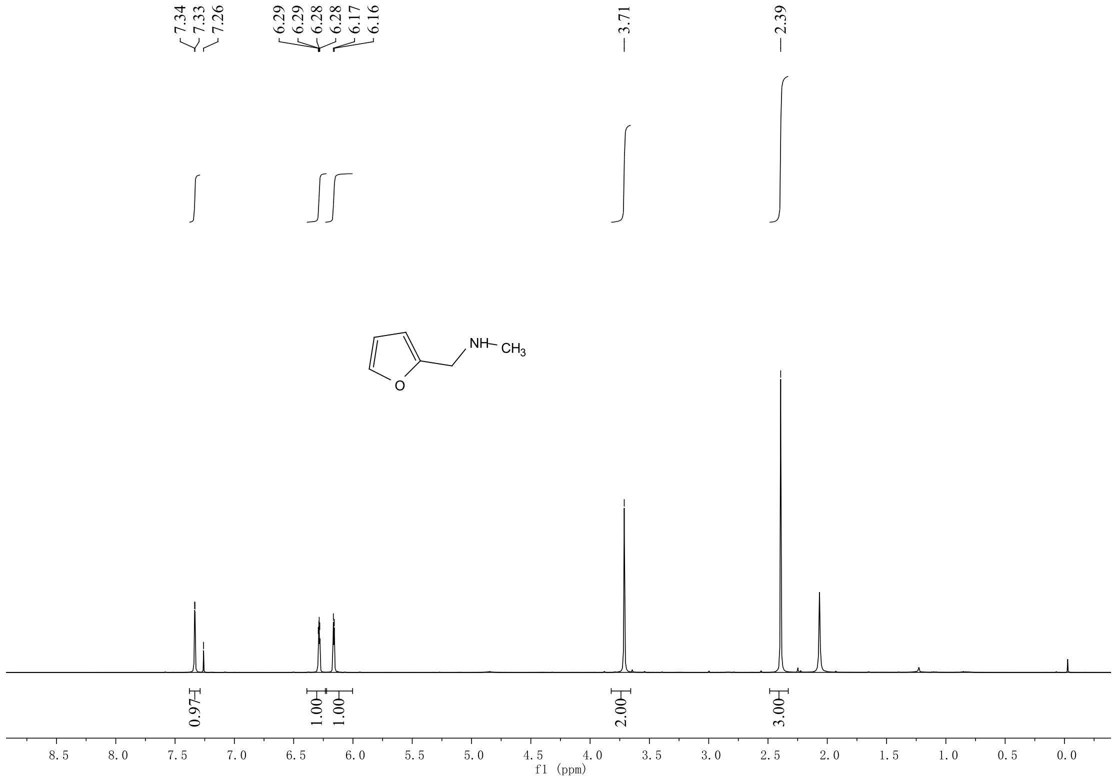

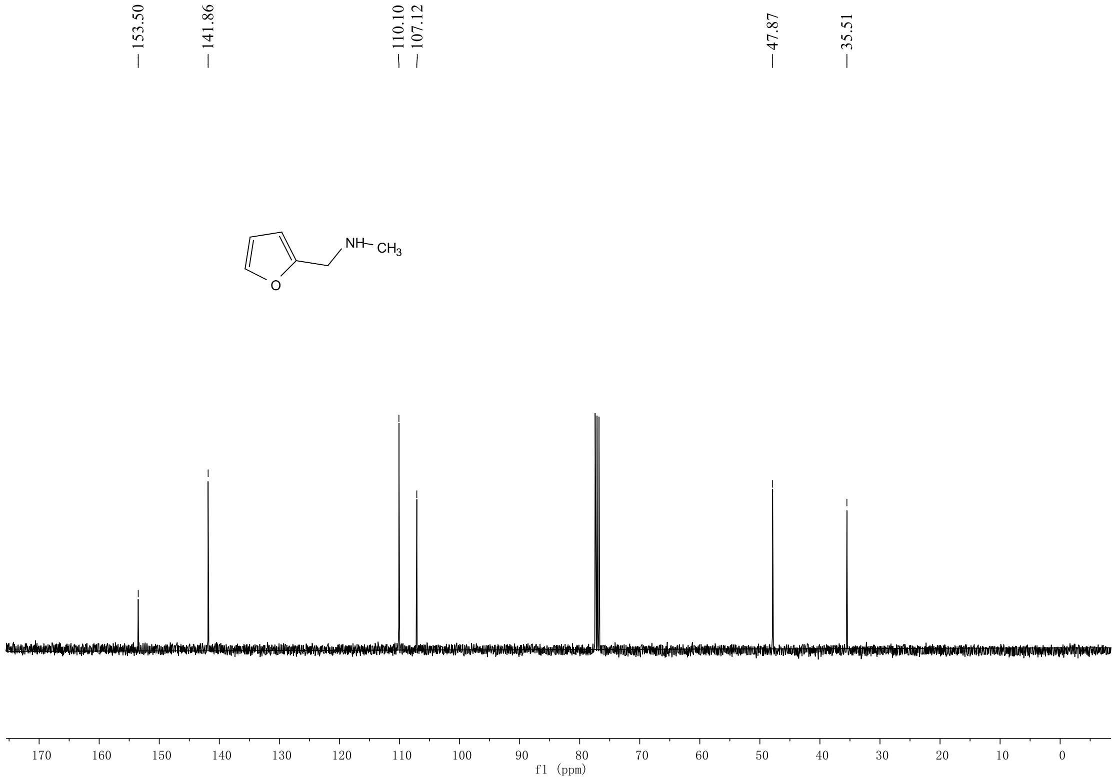

***N*,*N*-Dimethyl-2-phenylethanamine (6c)**

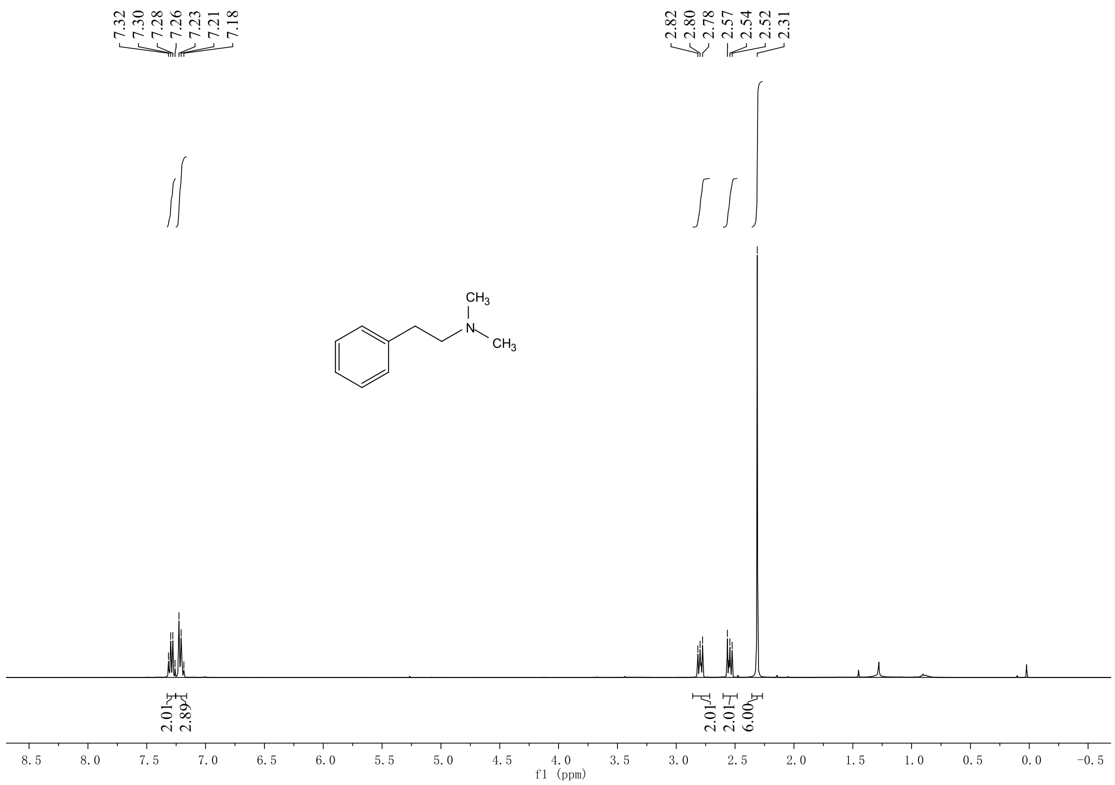

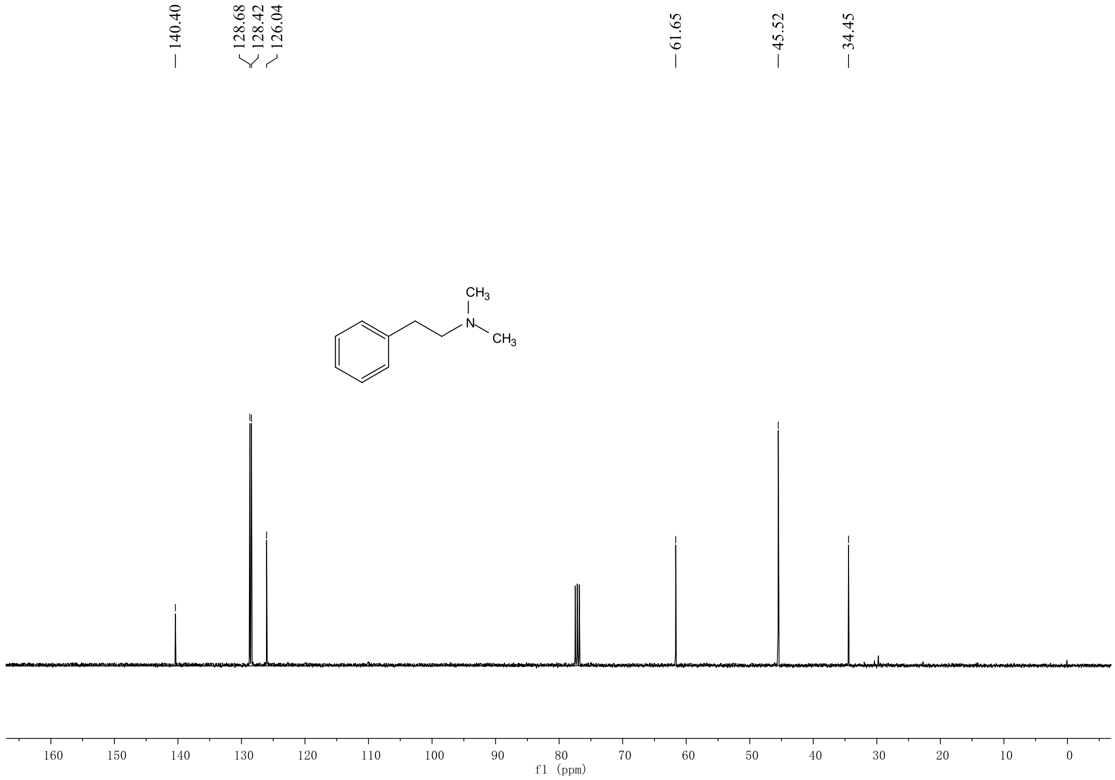

_N_-Benzyl-_N_-methyl-1-phenylmethanamine (7c)

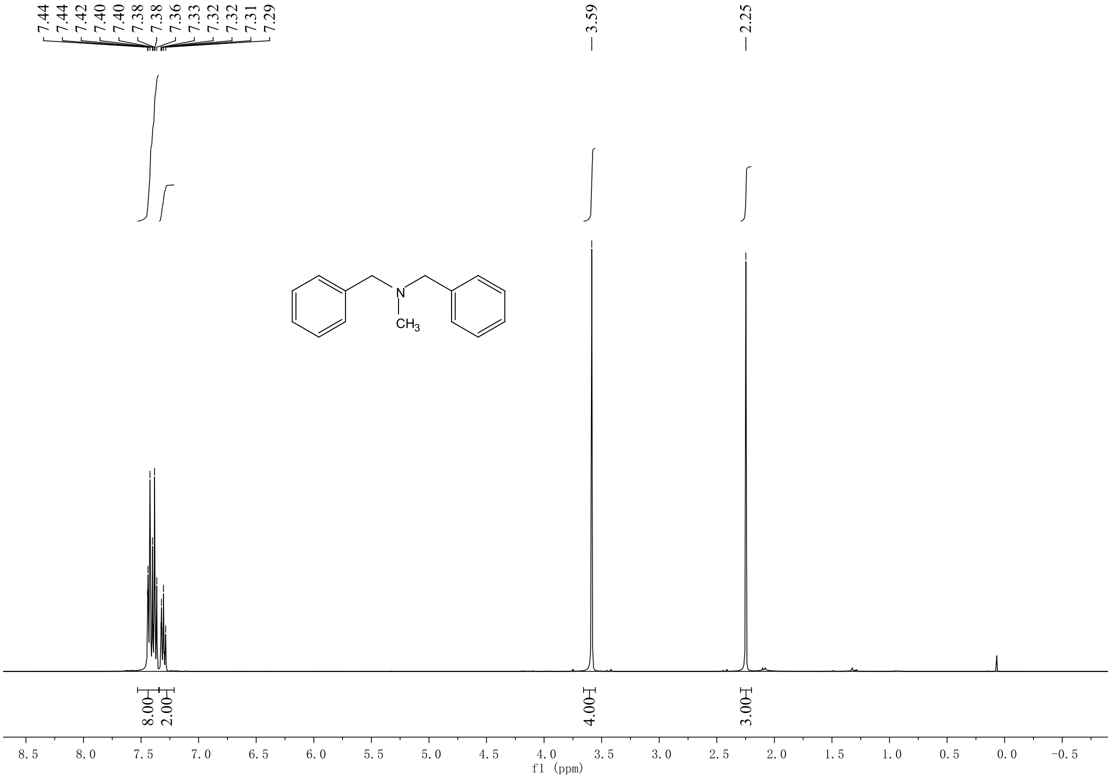

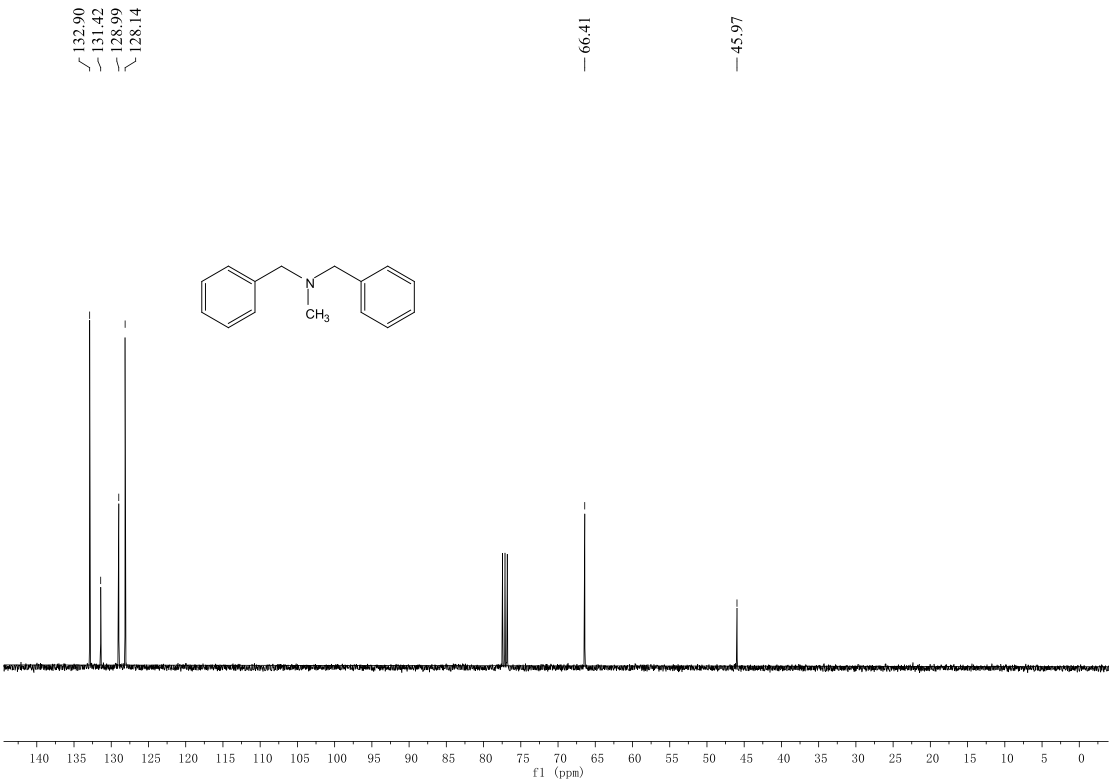

***N*-Methylaniline (8c)**

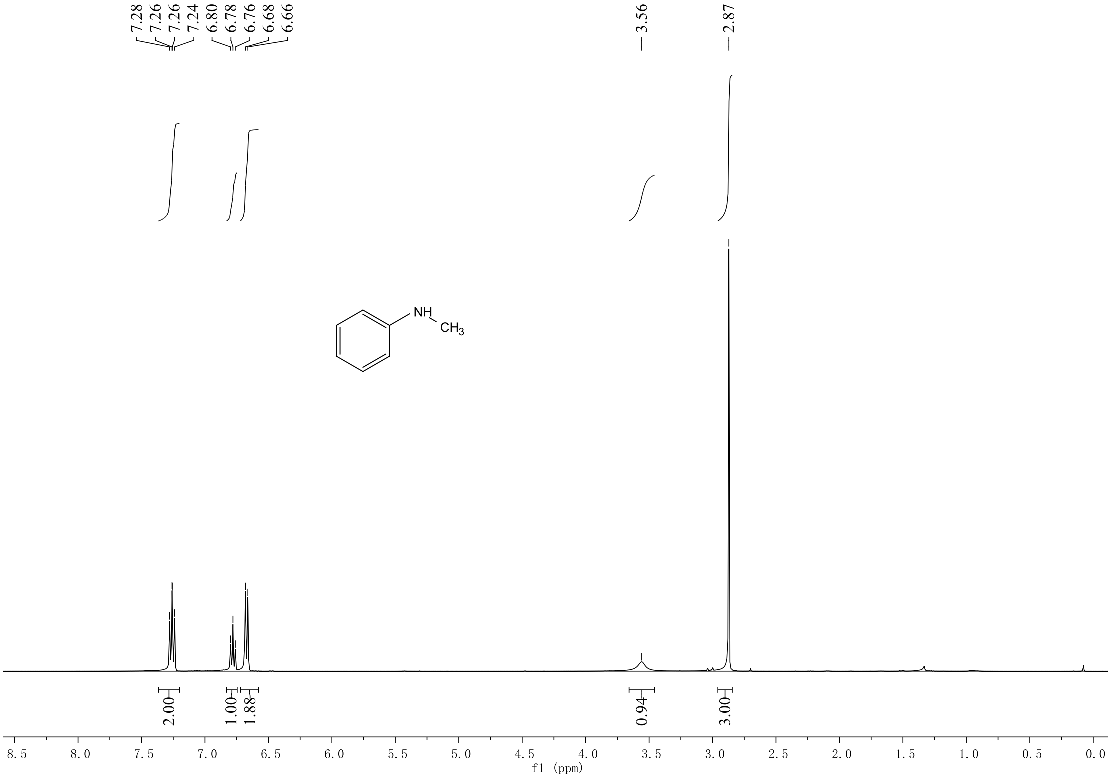

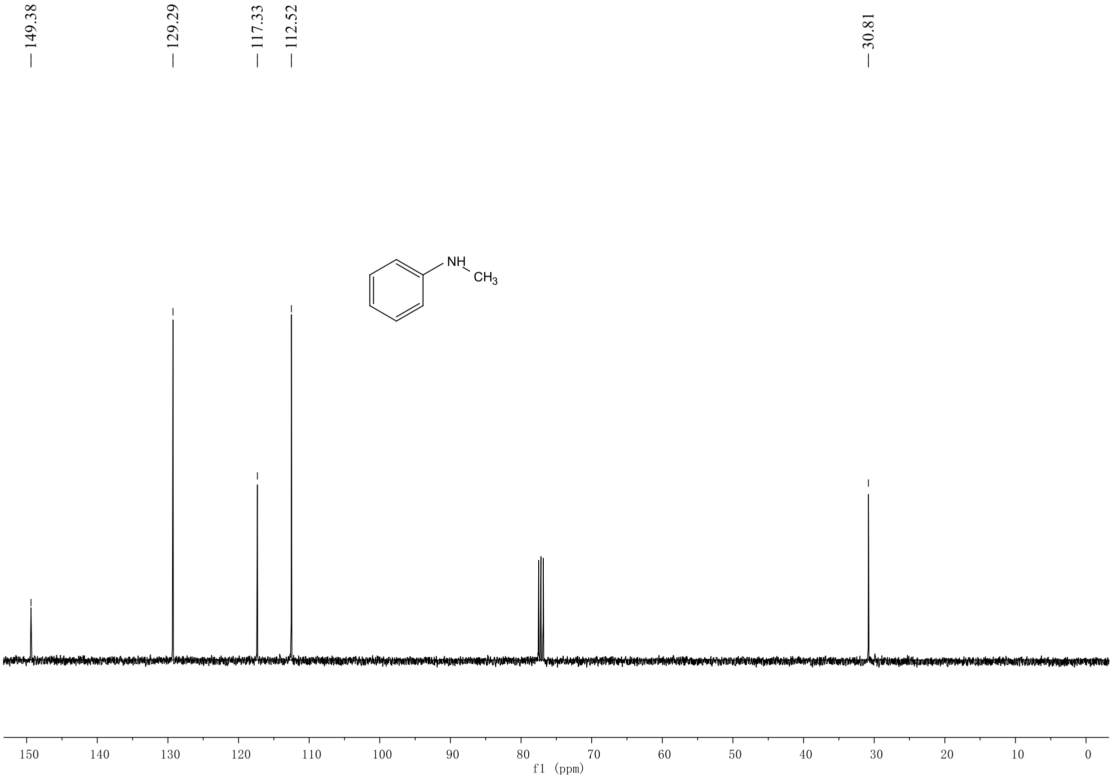

***N*,3-Dimethylaniline (9c)**

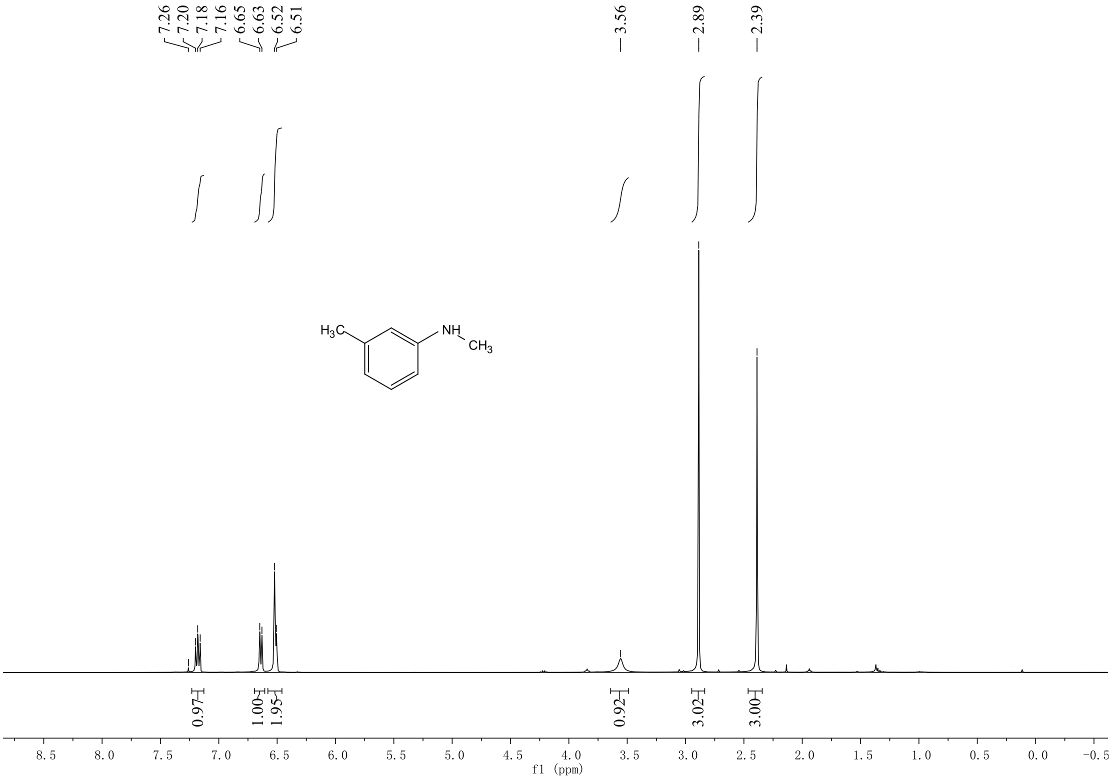

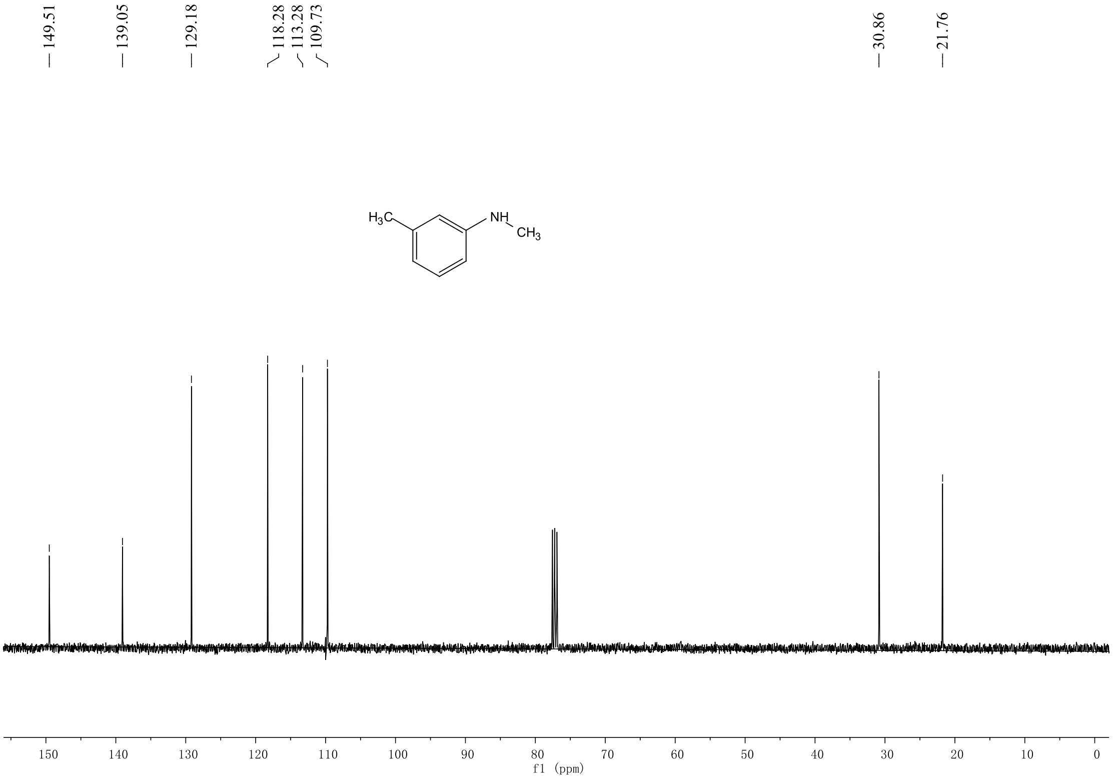

N,4-Dimethylaniline (10c)

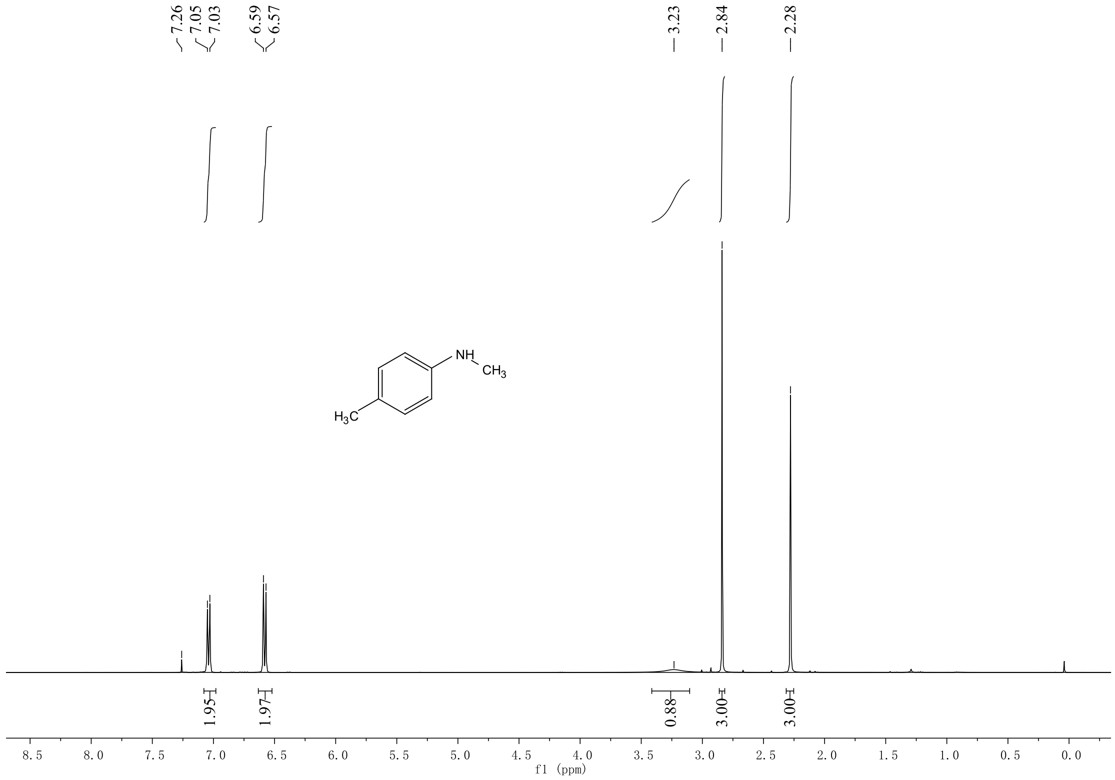

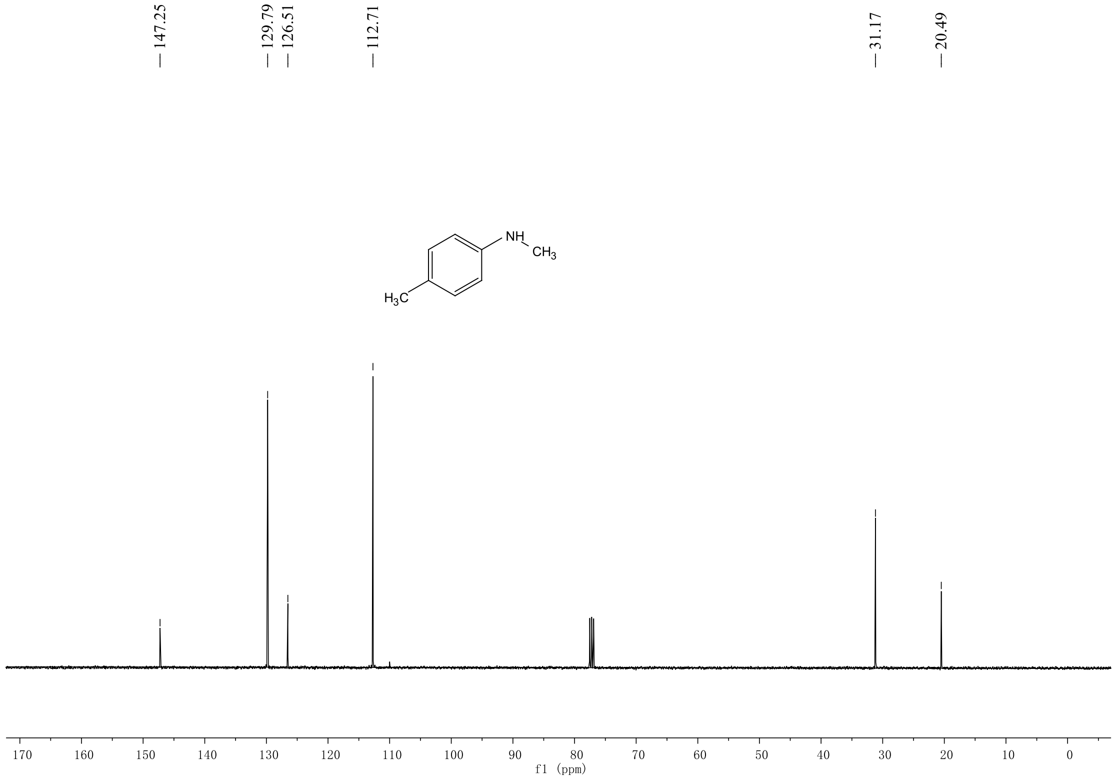

N-Methyl-4-nitroaniline (11c)

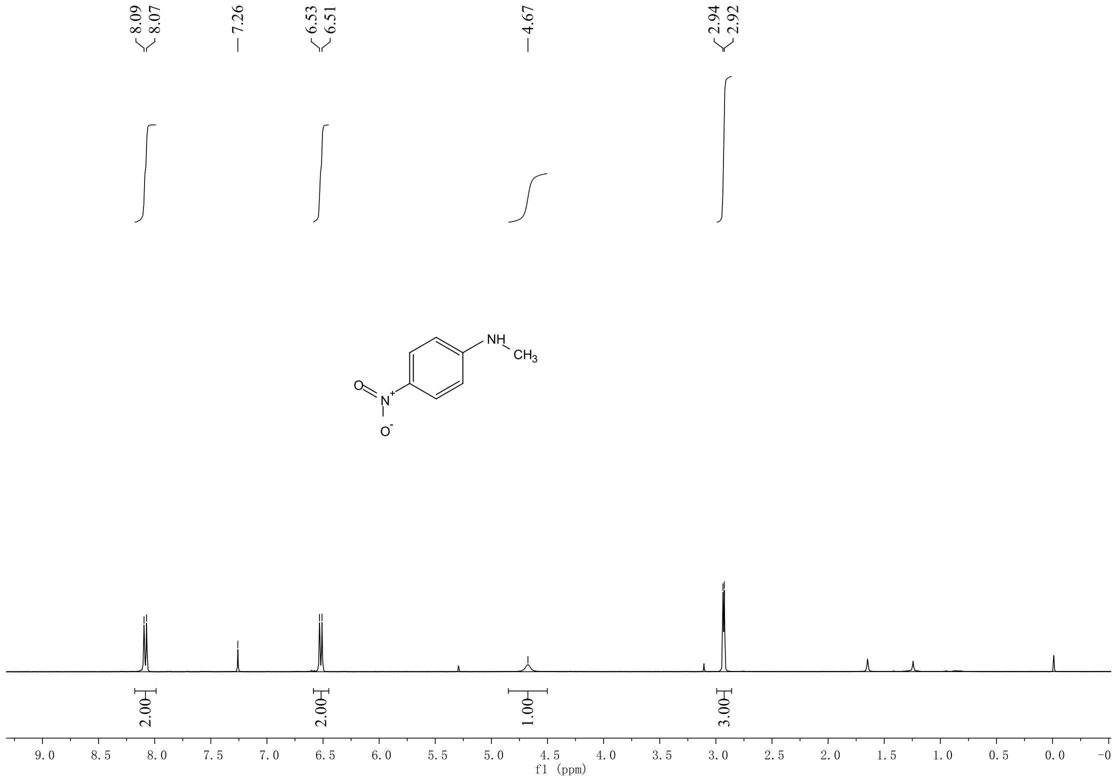

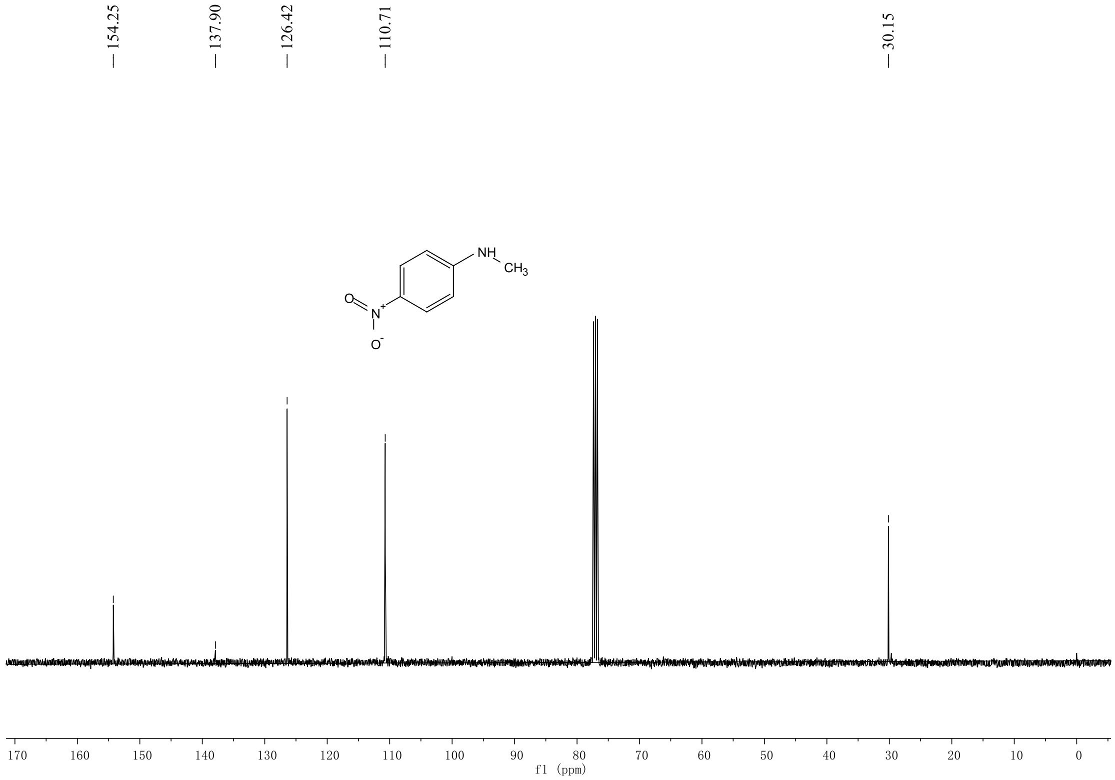

***N*,*N*-Dimethylaniline (12c)**

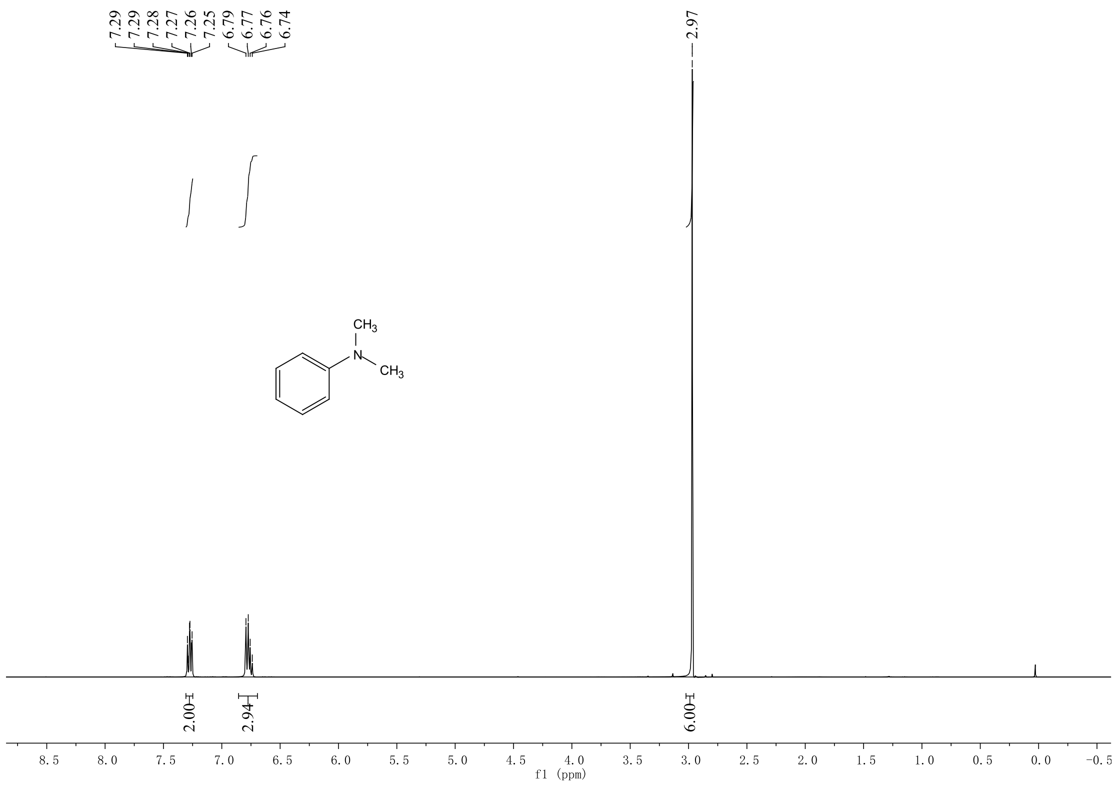

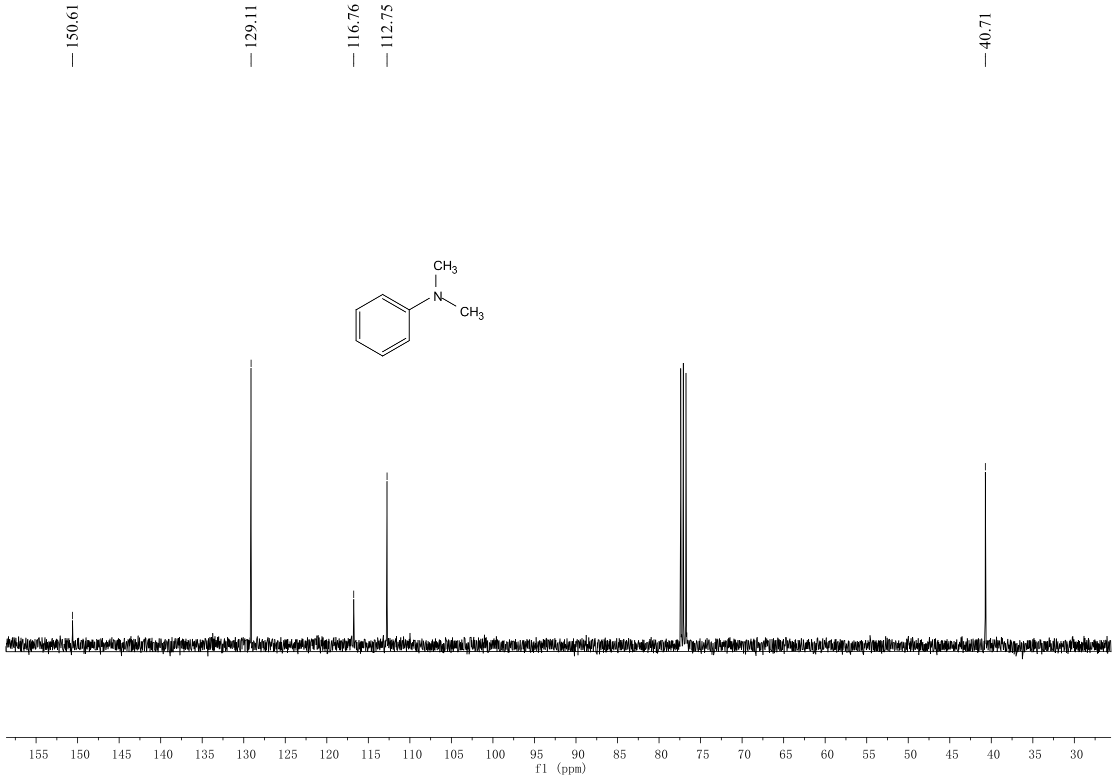

***N*-Methyl-*N*-phenylaniline (13c)**

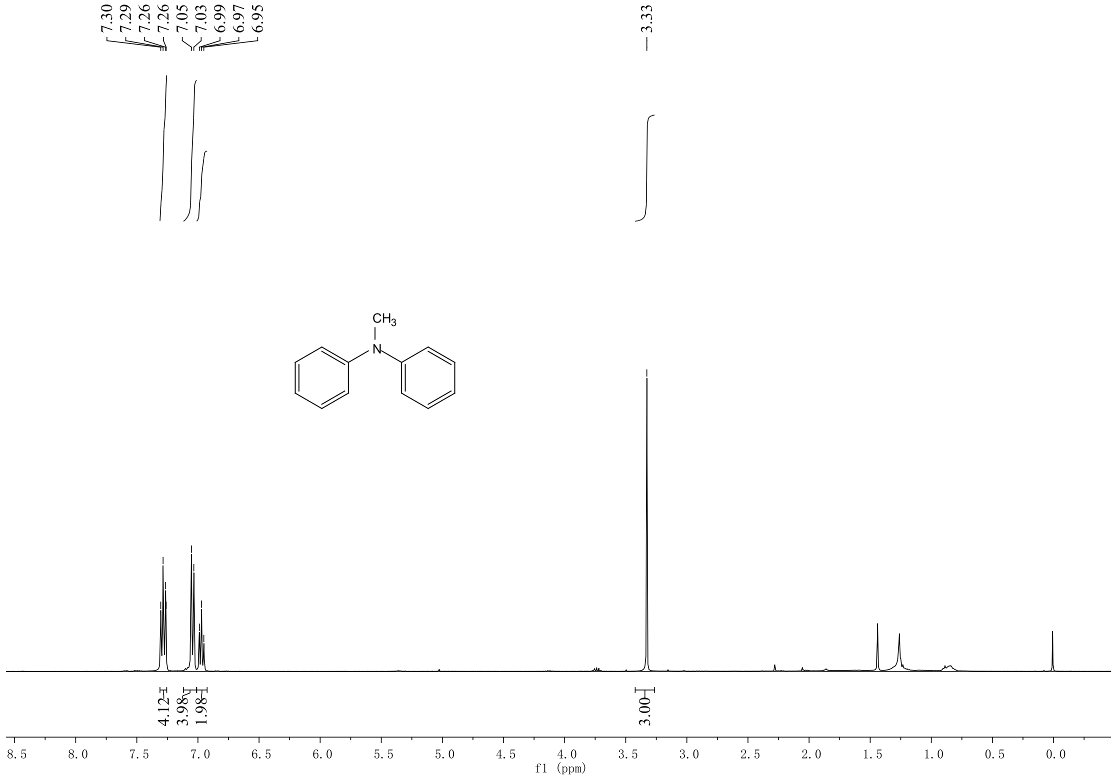

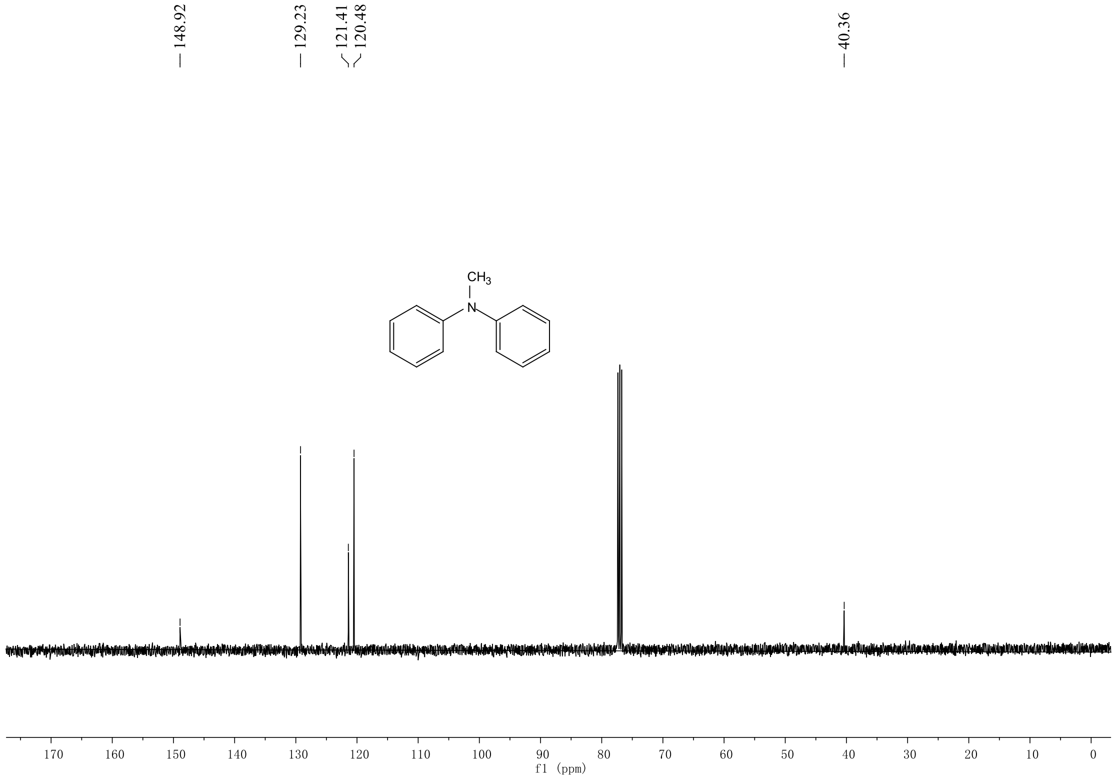

N^1,N^4-Dimethylbenzene-1,4-diamine (14c)

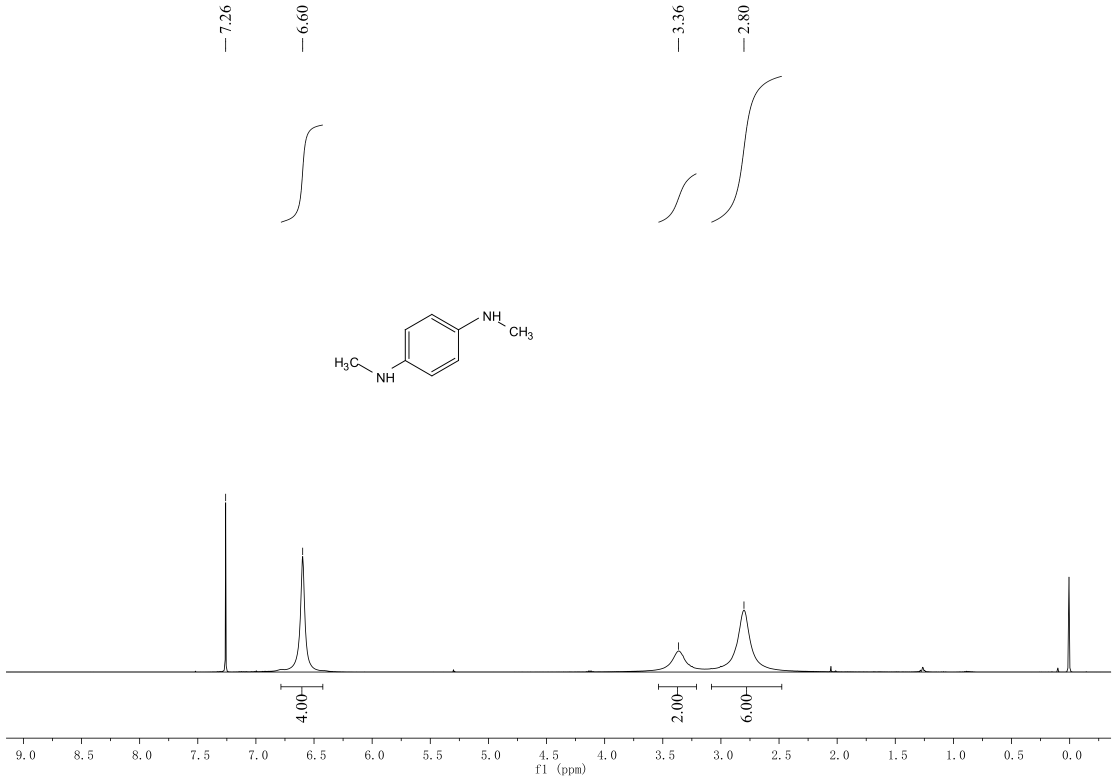

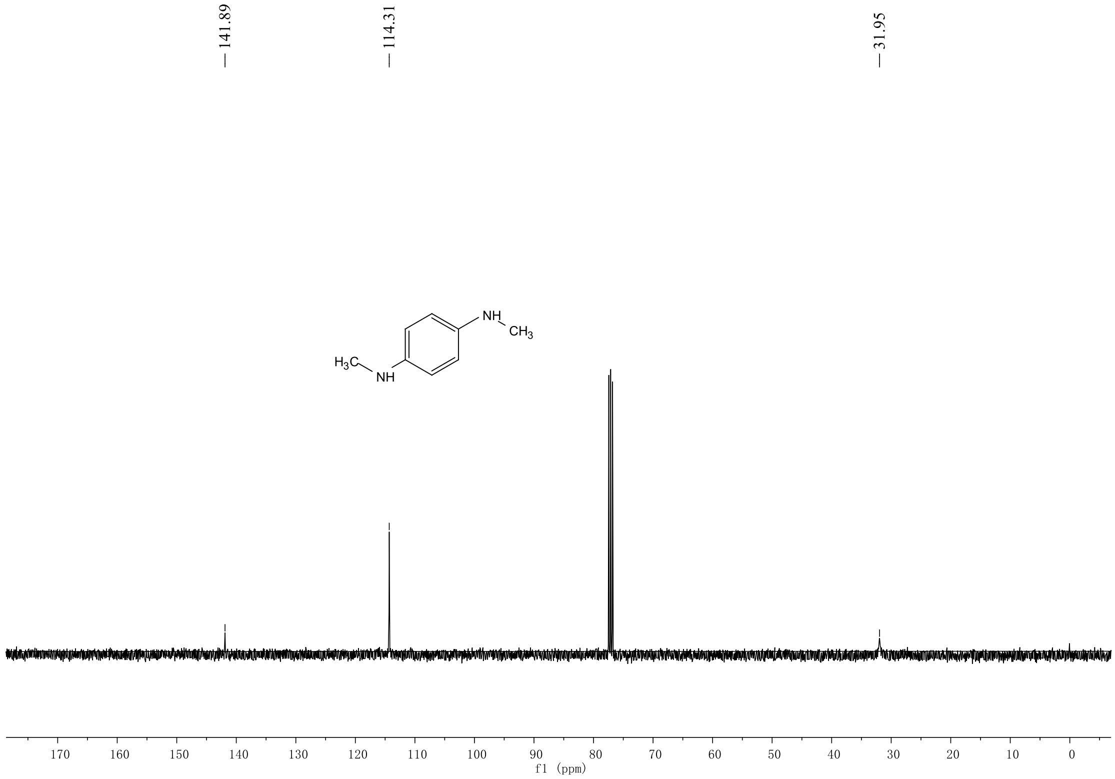

***N*-Methyl-cyclohexylamine (15c)**

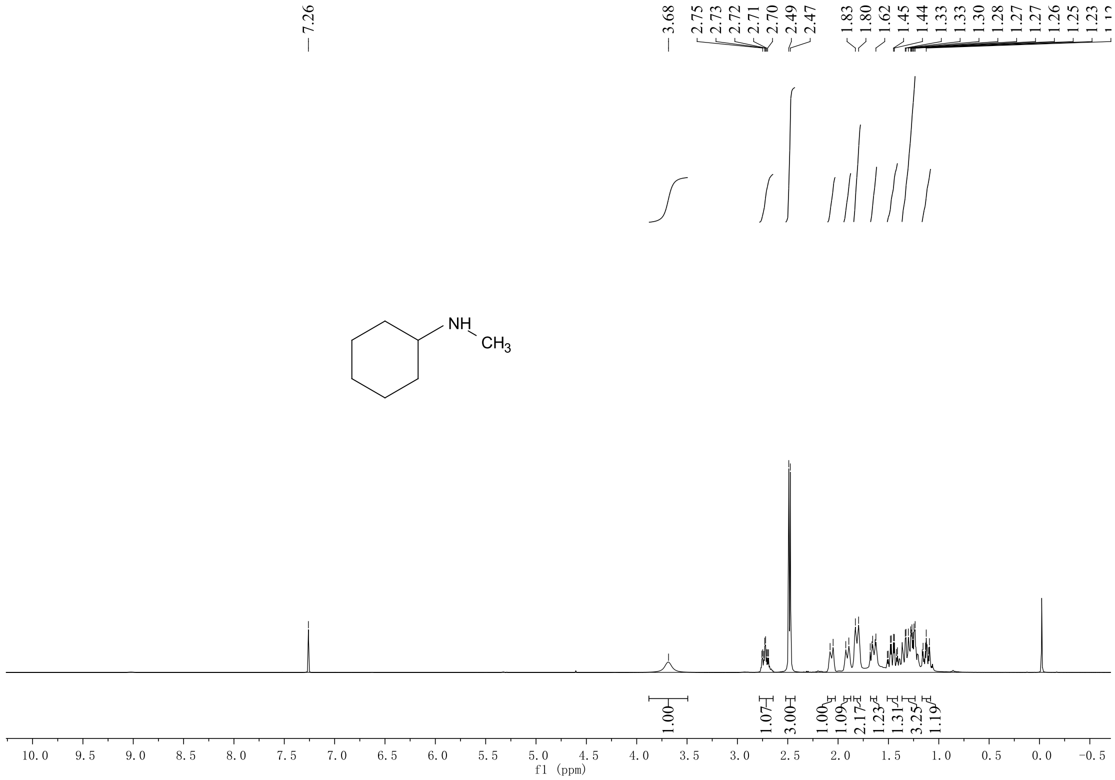

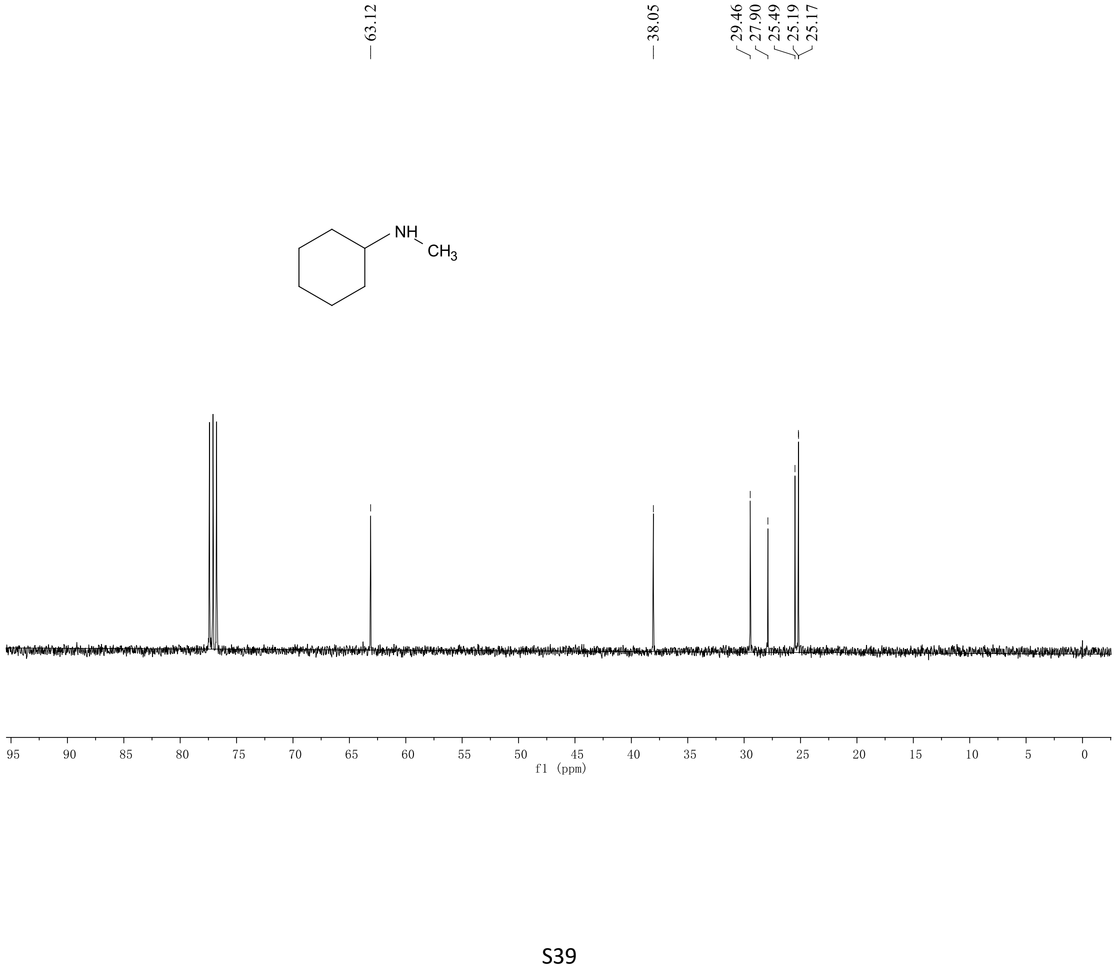

4-Chloro-*N*-methylaniline (16c)

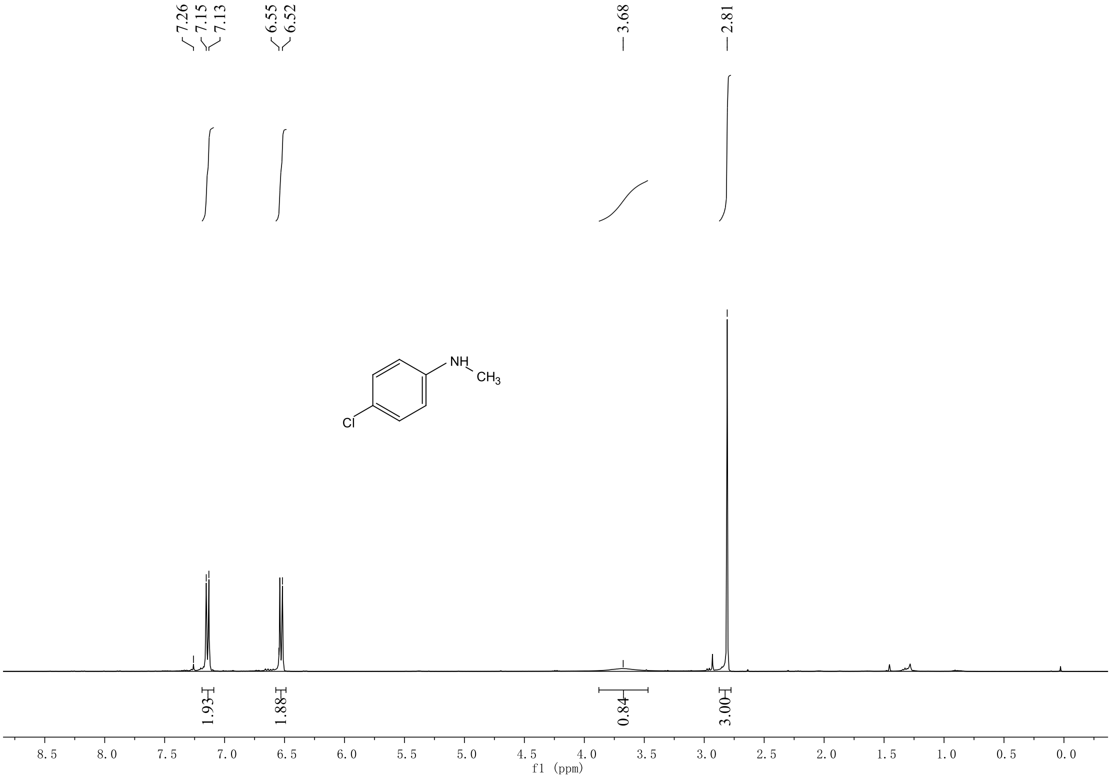

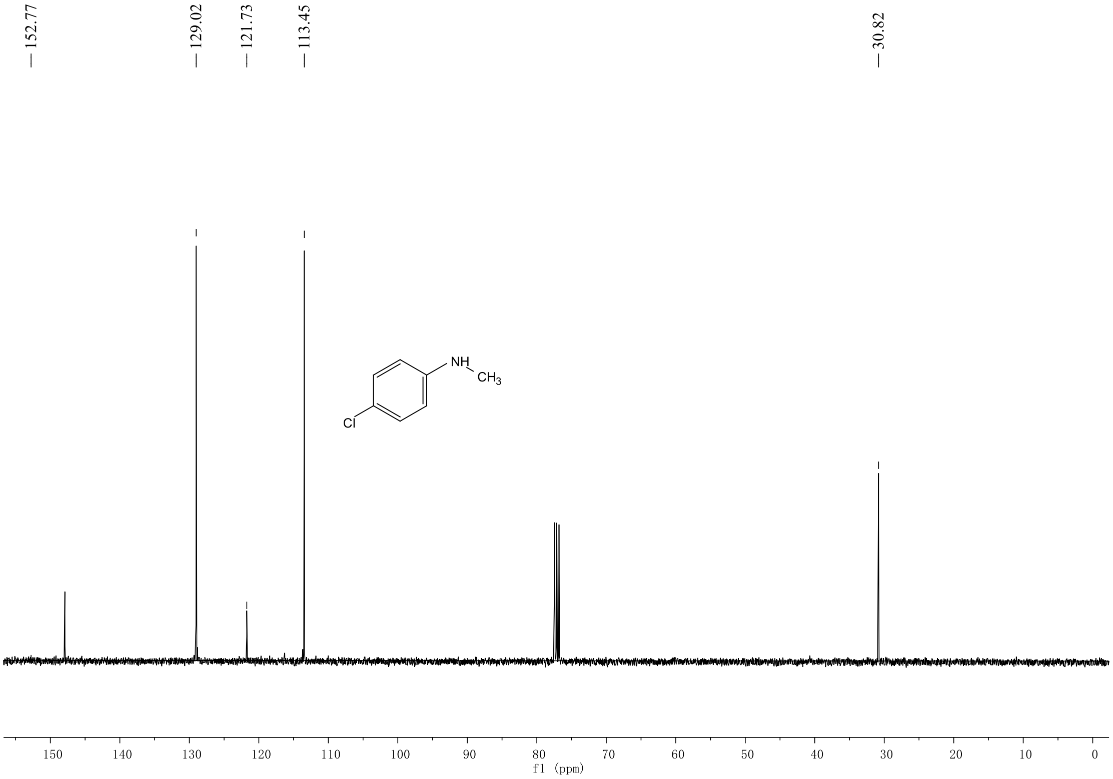

N-Methyl-4-(trifluoromethoxy)aniline (17c)

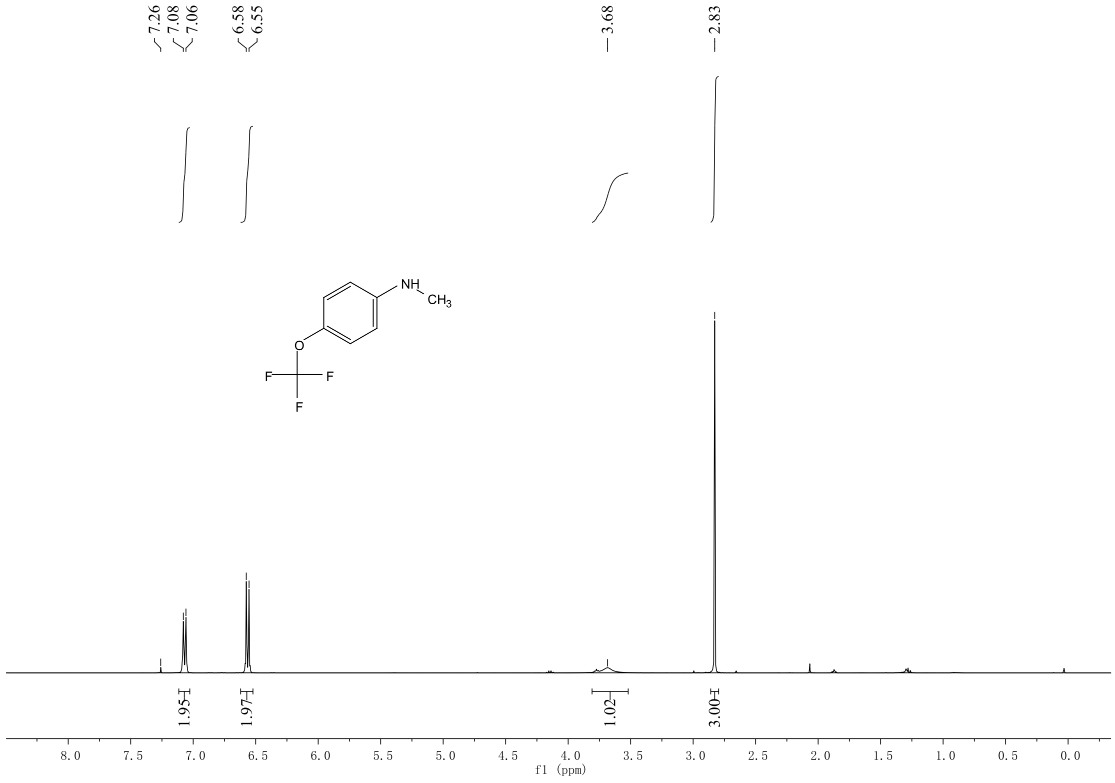

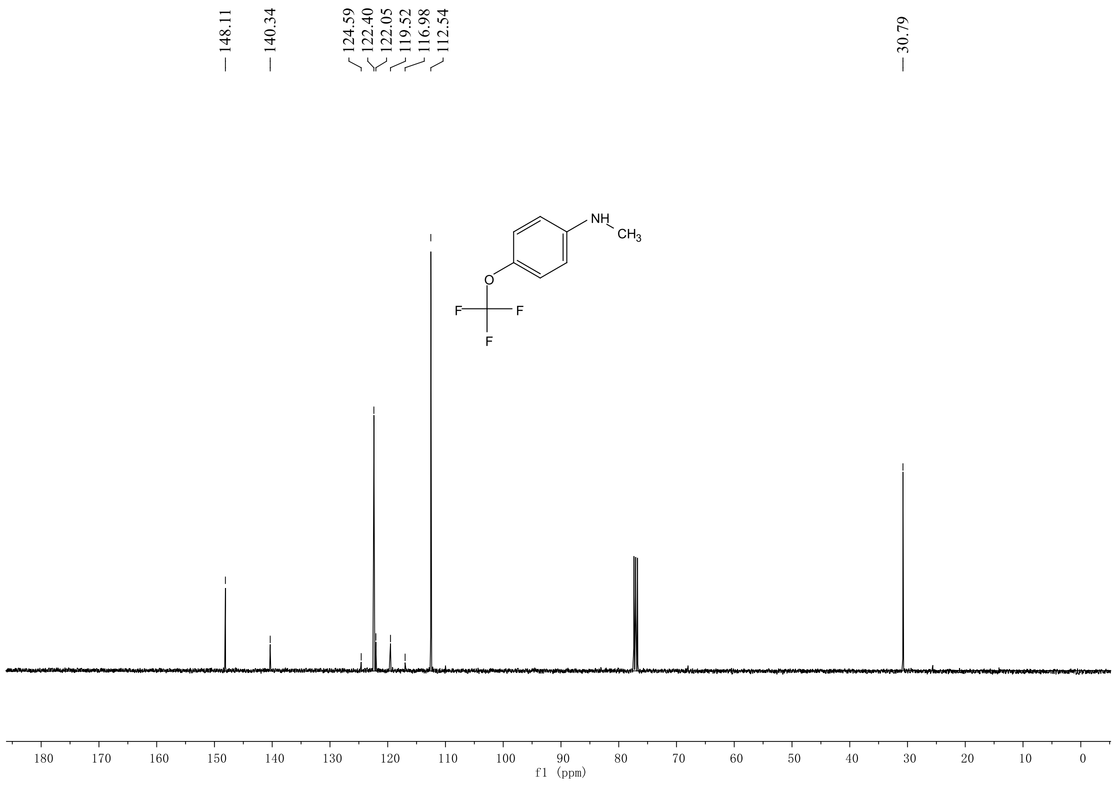

***N*-Methylnonan-1-amine (18c)**

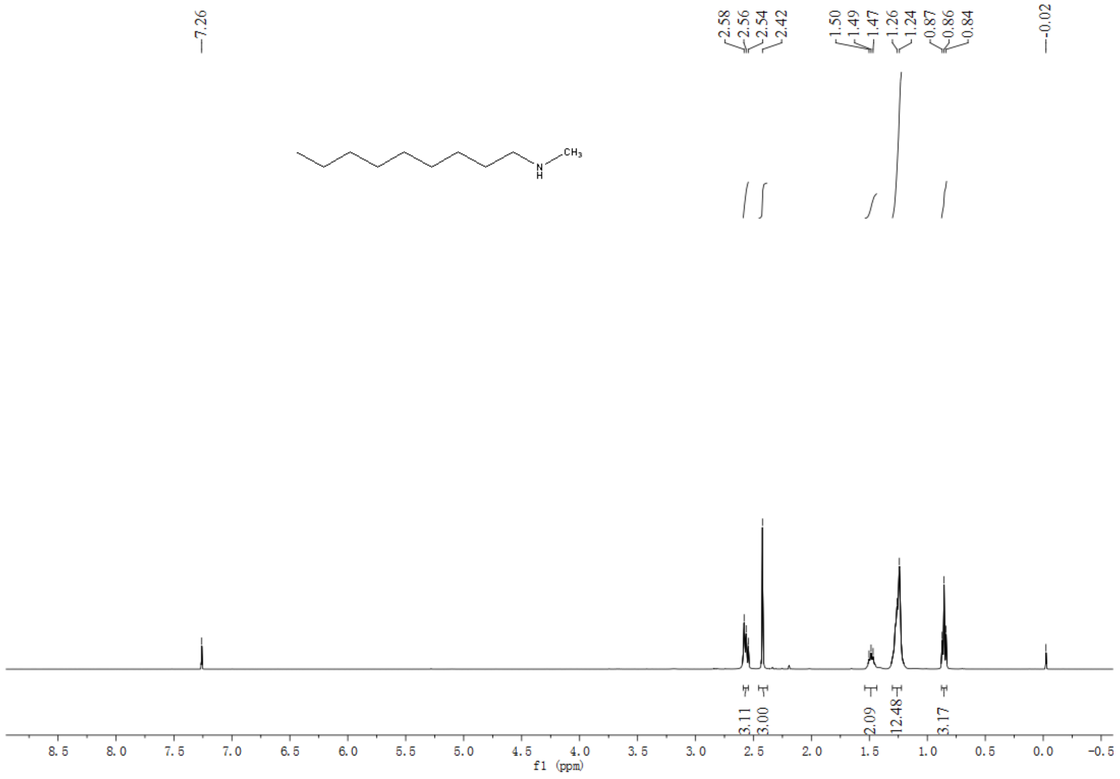

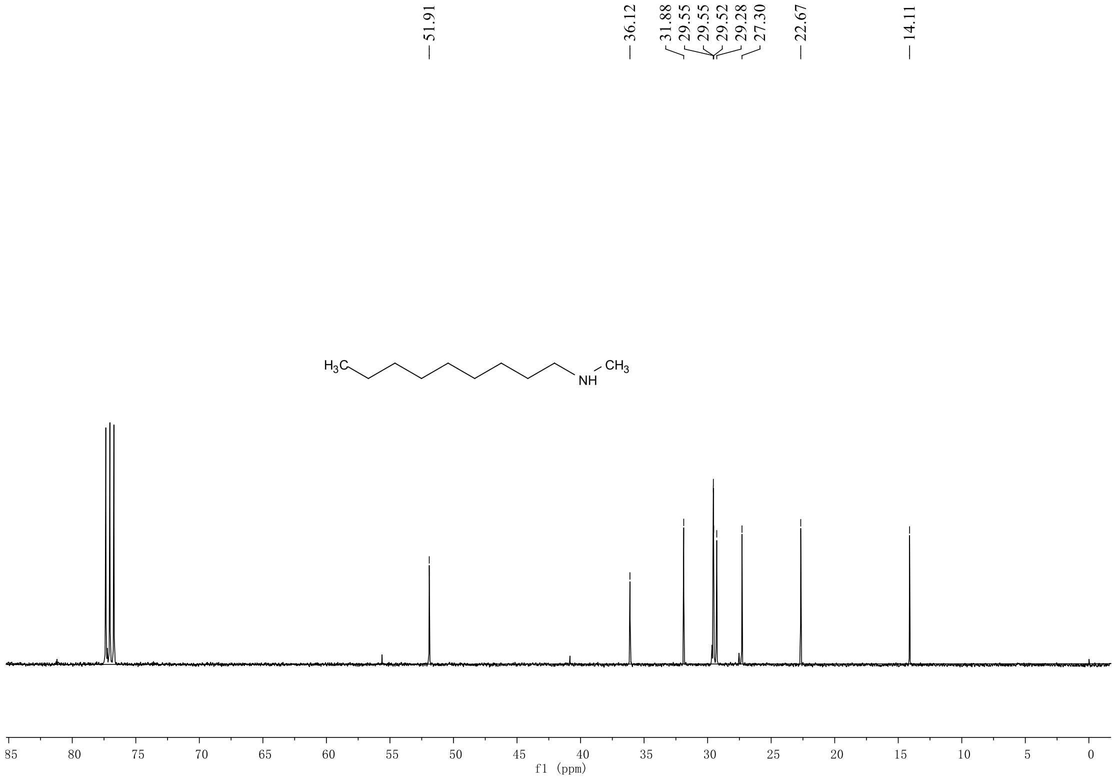

***N*-(5-(Methylamino)pentyl)acetamide (19c)**

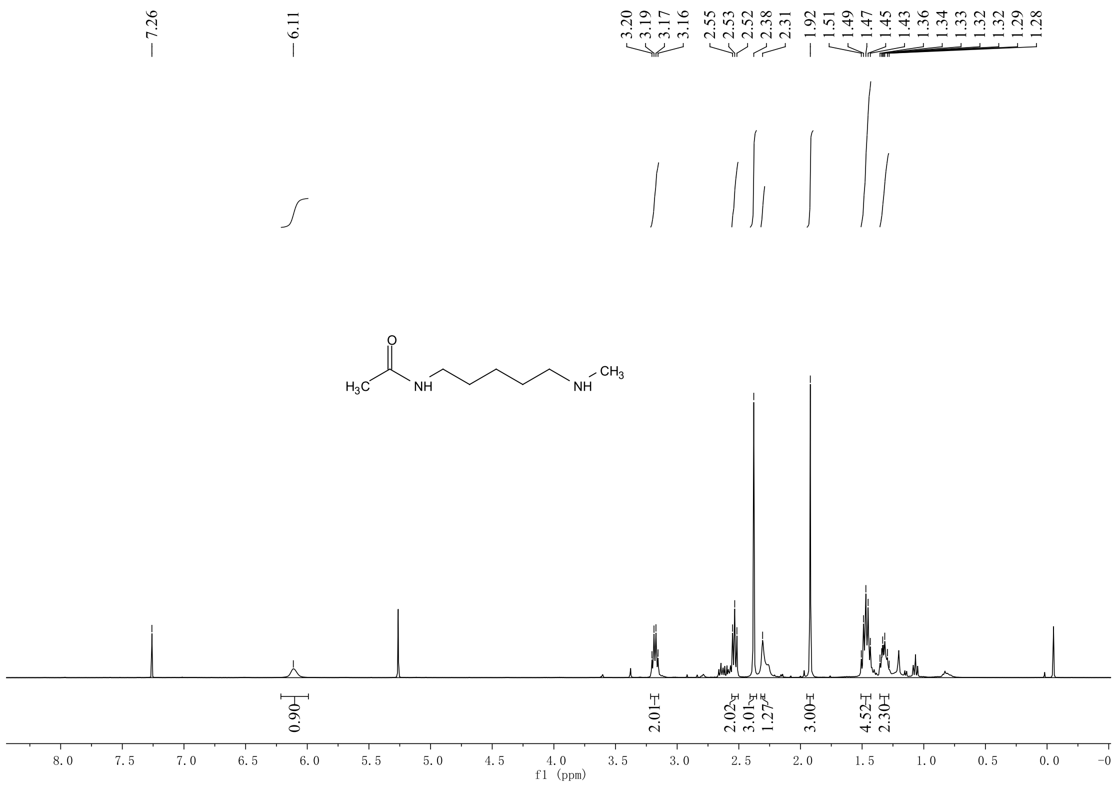

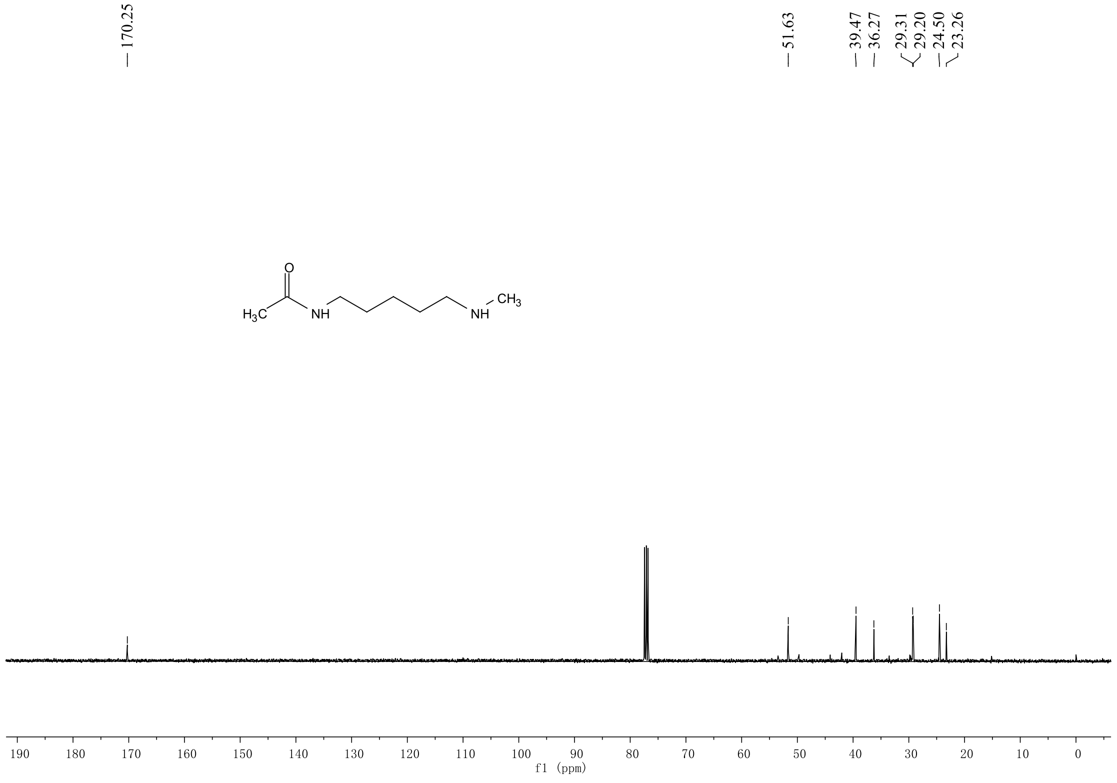

N-(4-(Methylamino)phenyl)acetamide (20c)

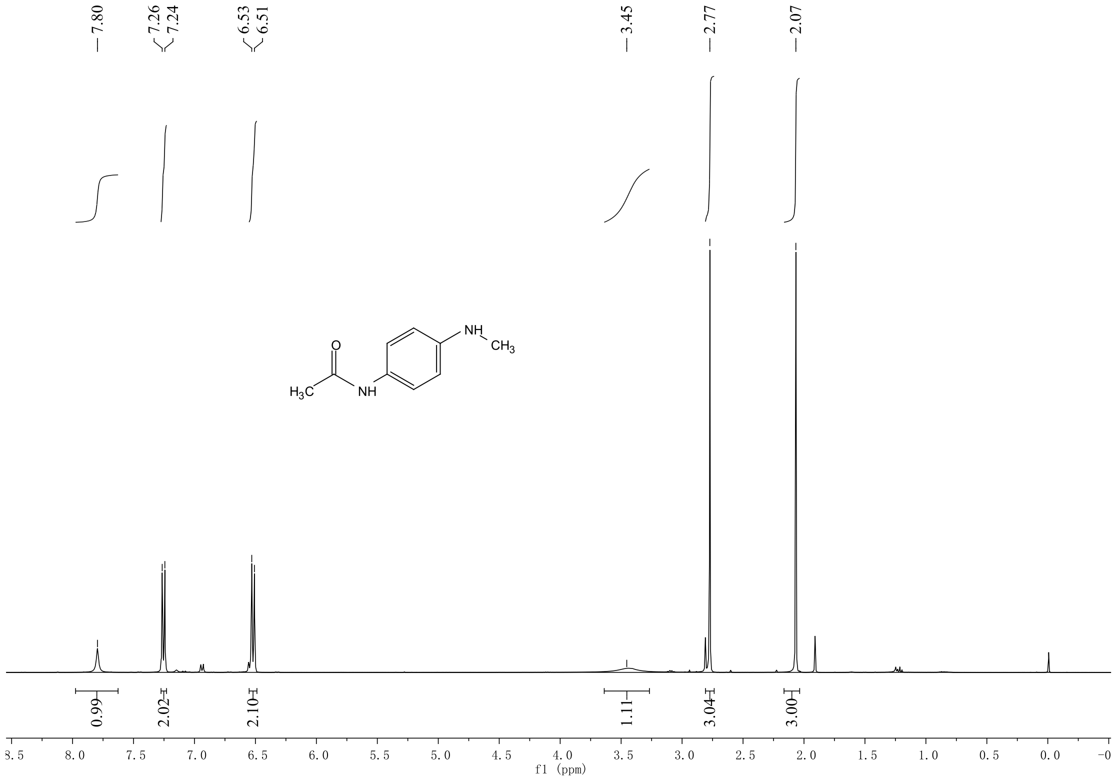

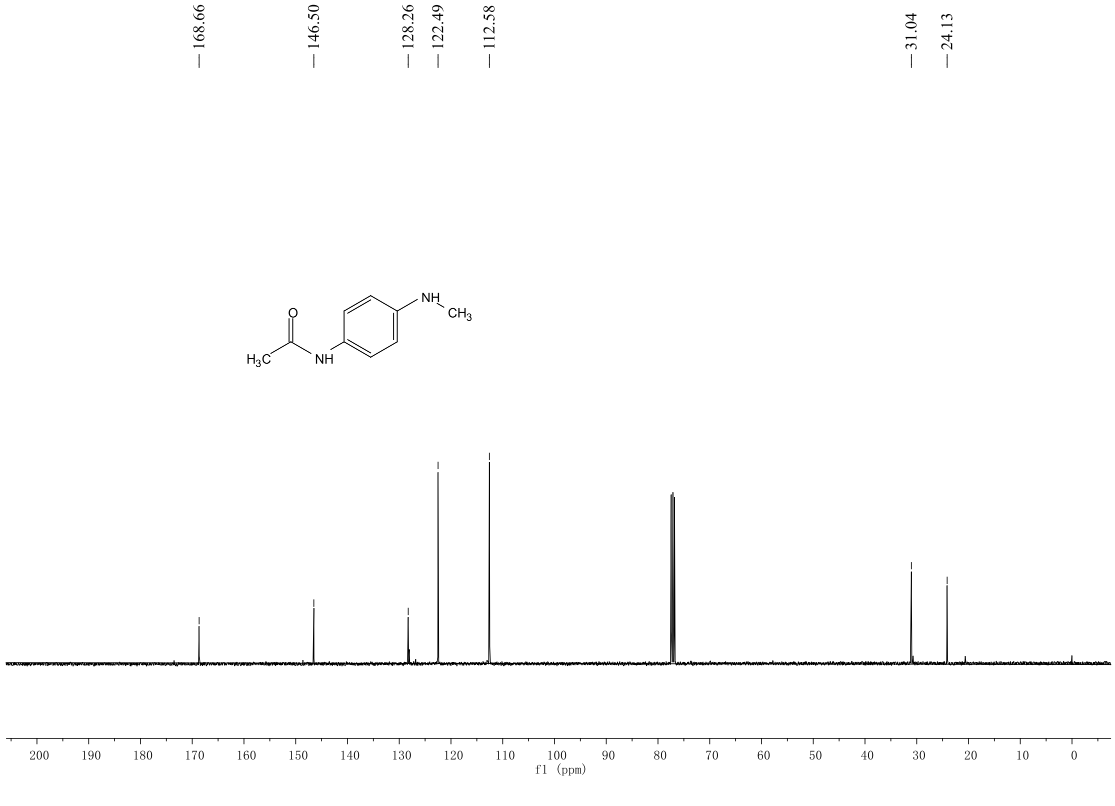

8. ESI–MS

N-Methyl-2-phenylethanamine (1c)

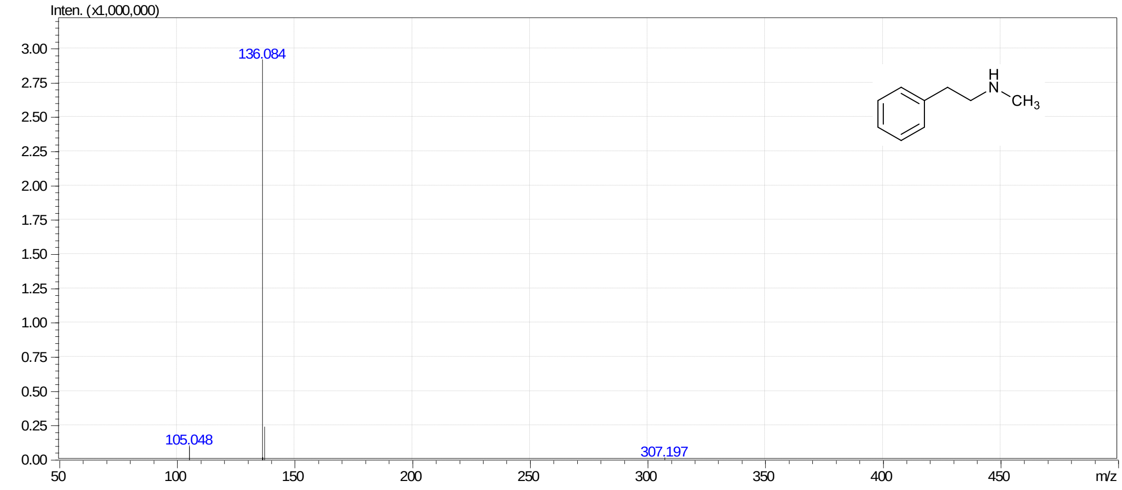

N-Methylbenzylamine (2c)

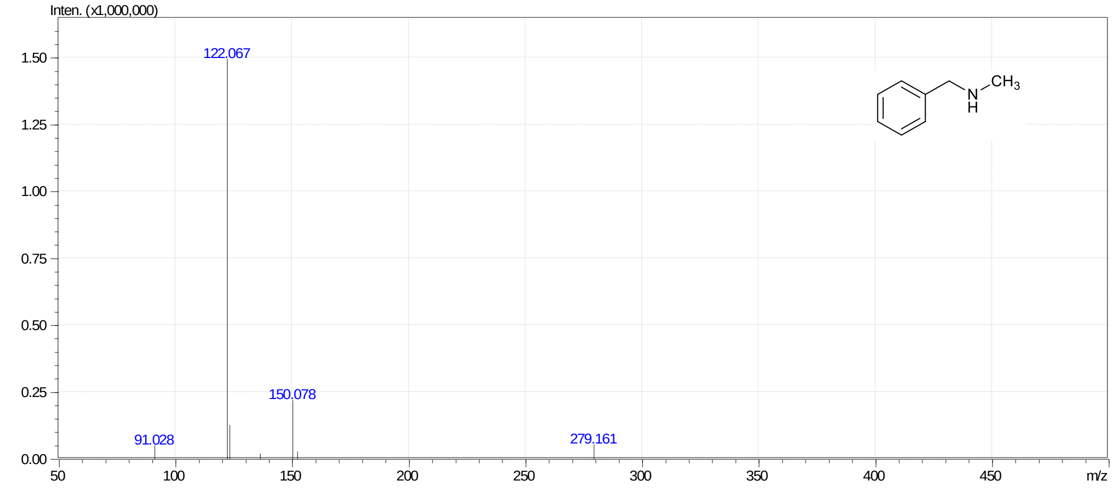

N-Methyl-4-chlorobenzylamine (3c)

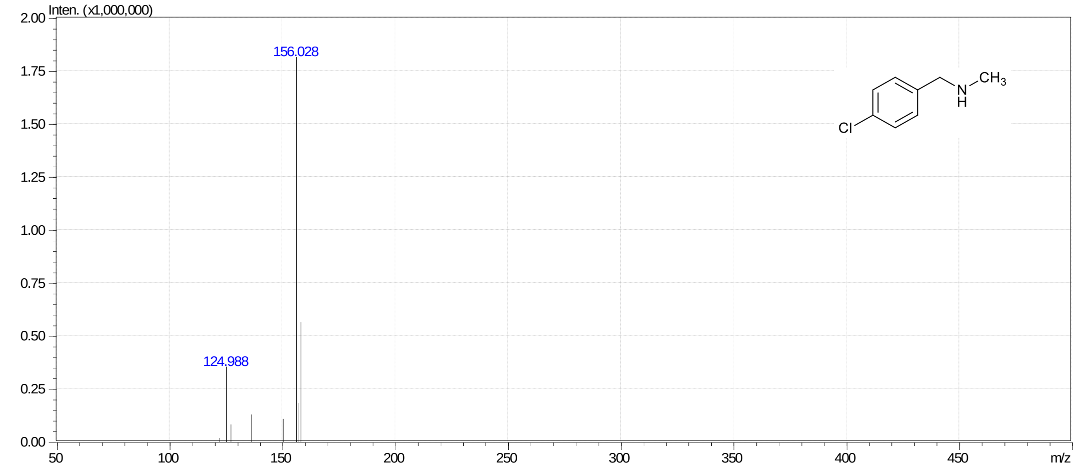

N-Methyl-α-phenylethylamine (4c)

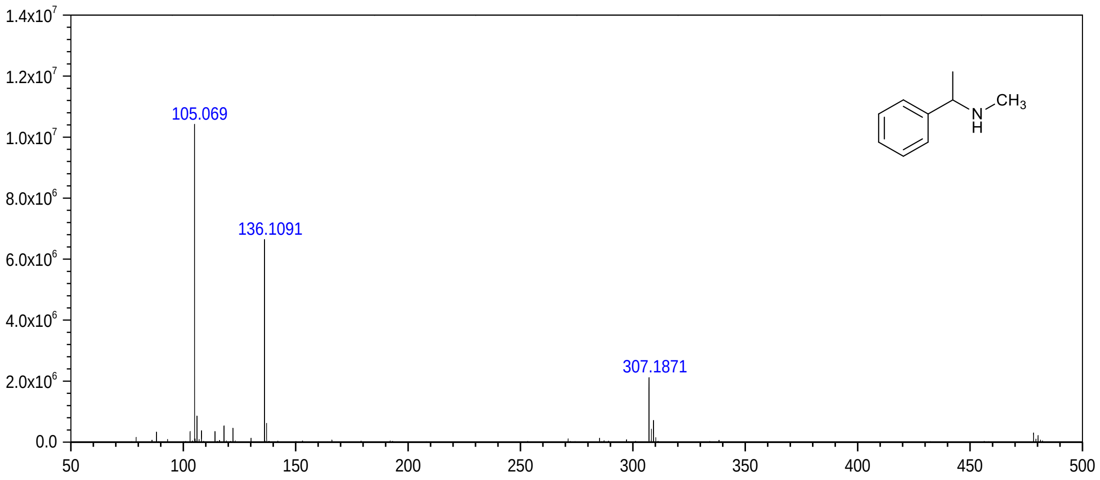

N-Methylfurfurylamine (5c)

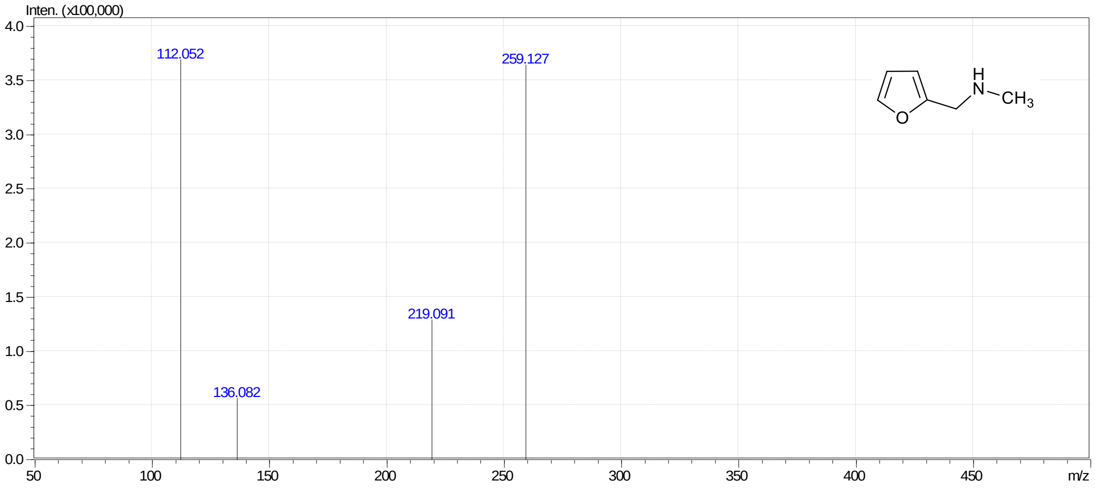

N,N-Dimethyl-2-phenylethanamine (6c)

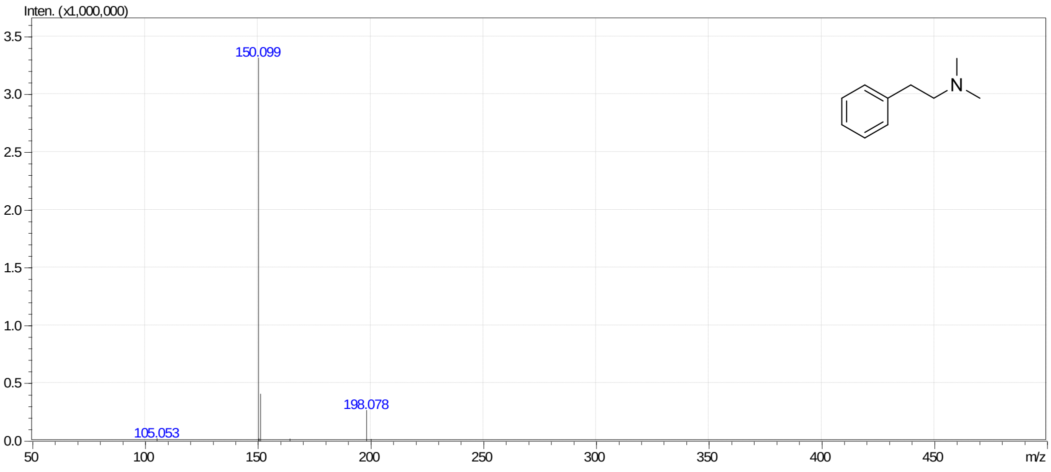

N-Methyldibenzylamine (7c)

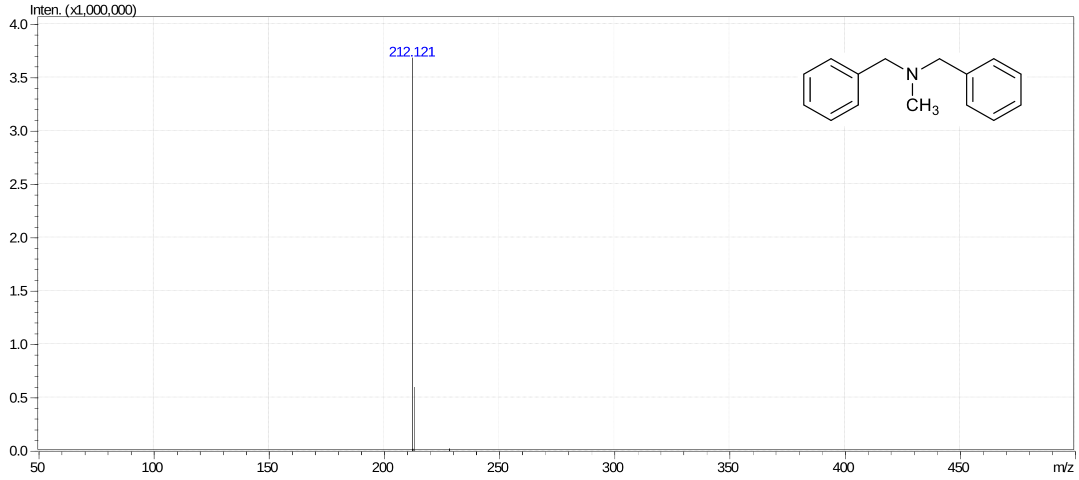

N-Methylaniline (8c)

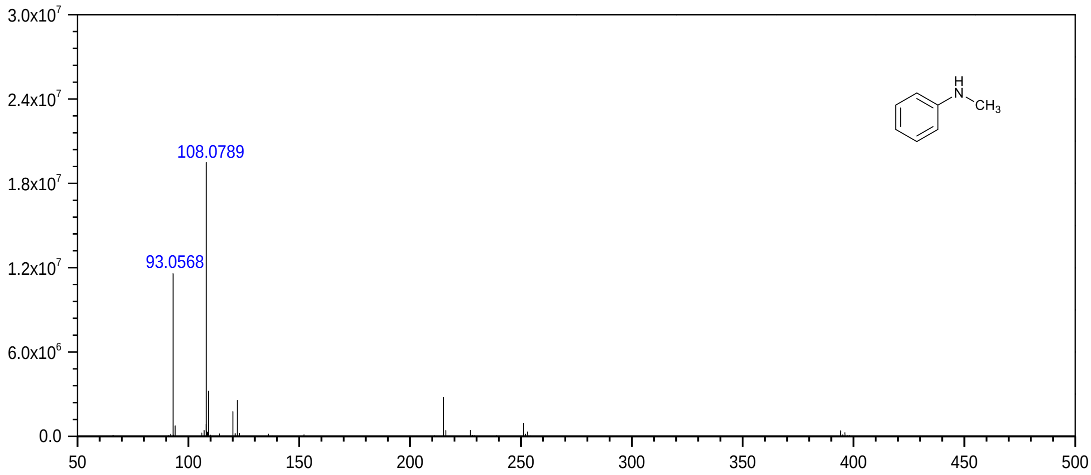

N,3-Dimethylbenzenamine (9c)

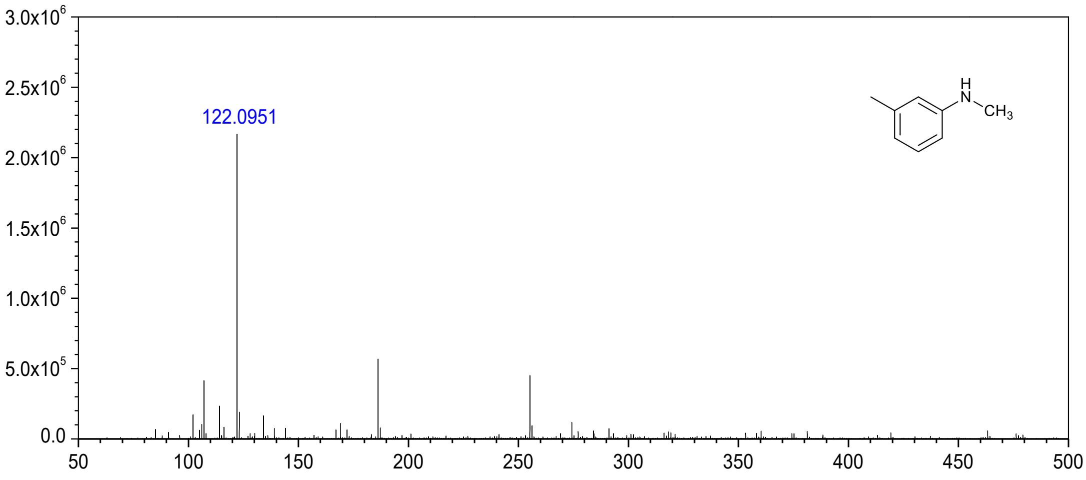

N,4-Dimethylbenzenamine (10c)

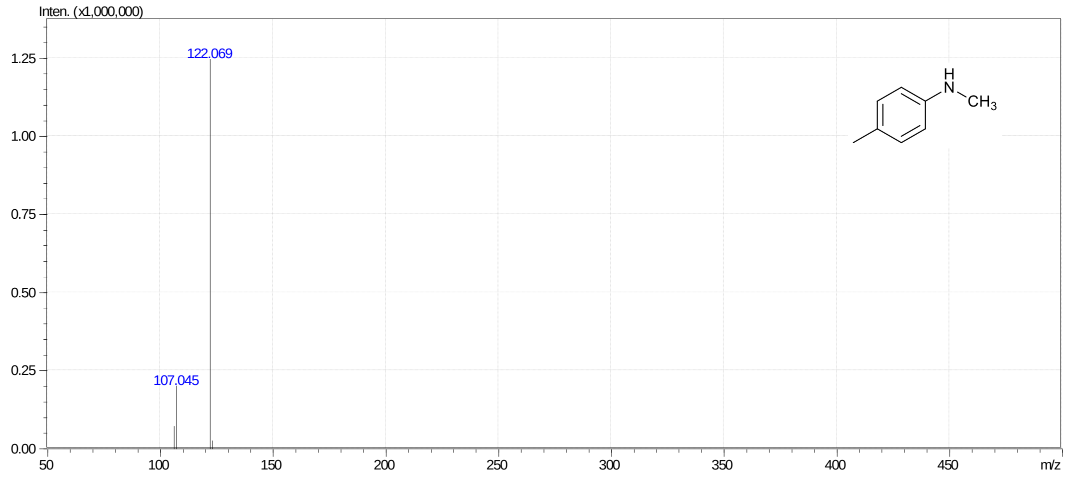

N-Methyl-4-nitrobenzenamine (11c)

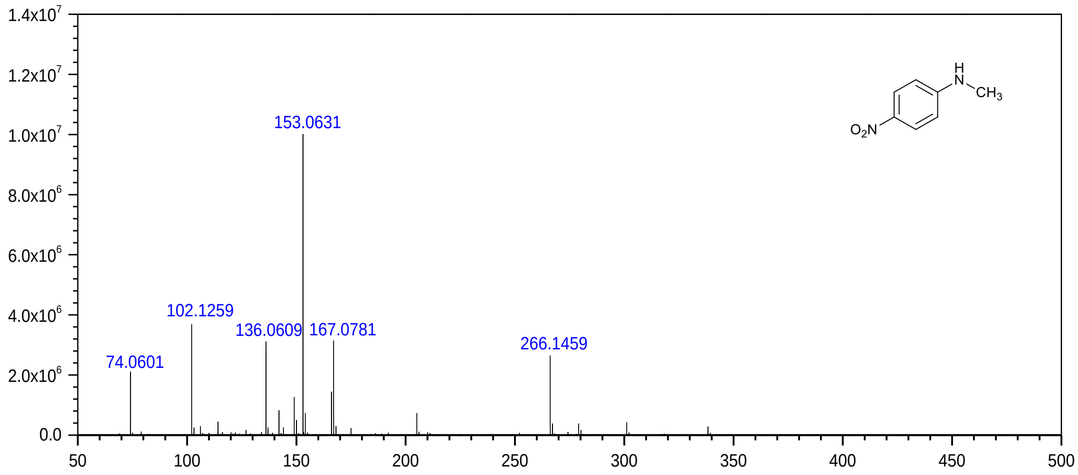

N,N-Dimethylaniline (12c)

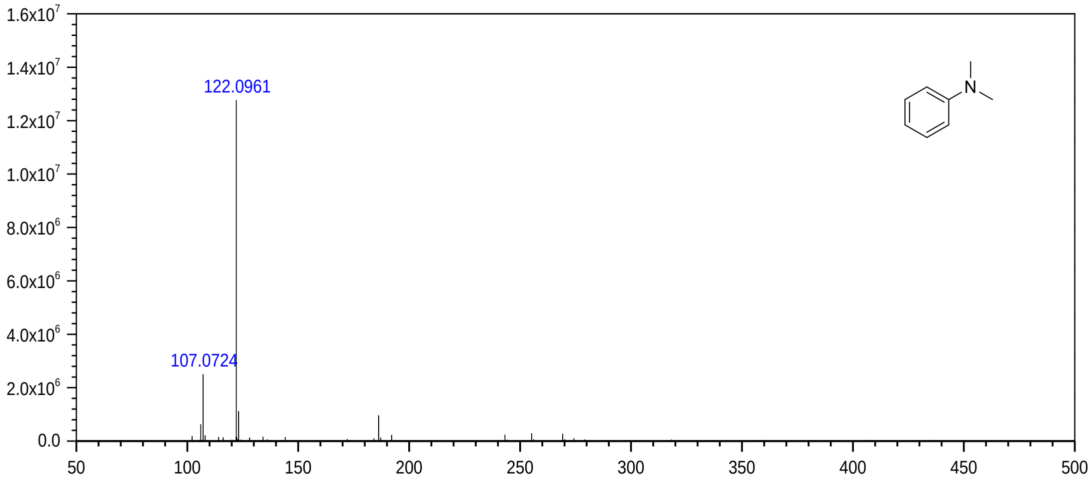

***N*-Methyldiphenylamine (13c)**

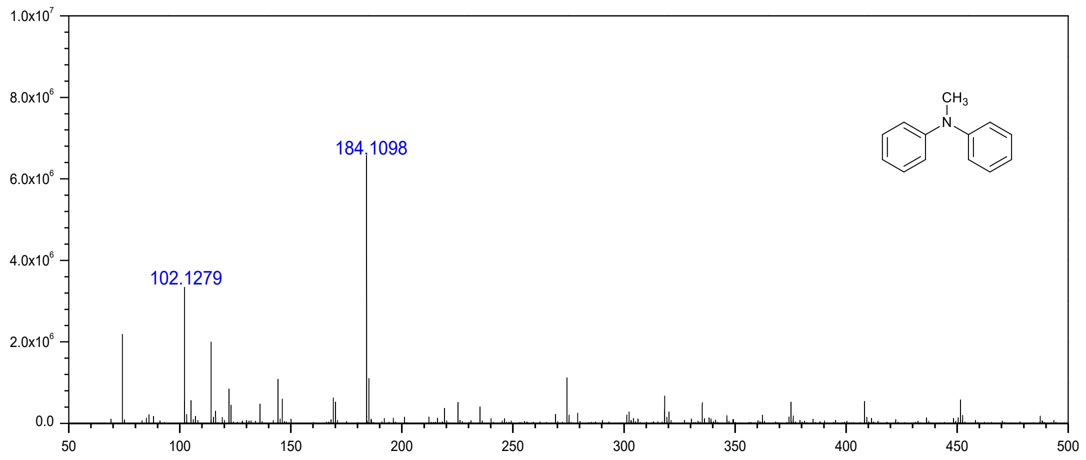

***N*¹,*N*⁴-Dimethyl-1,4-benzenediamine (14c)**

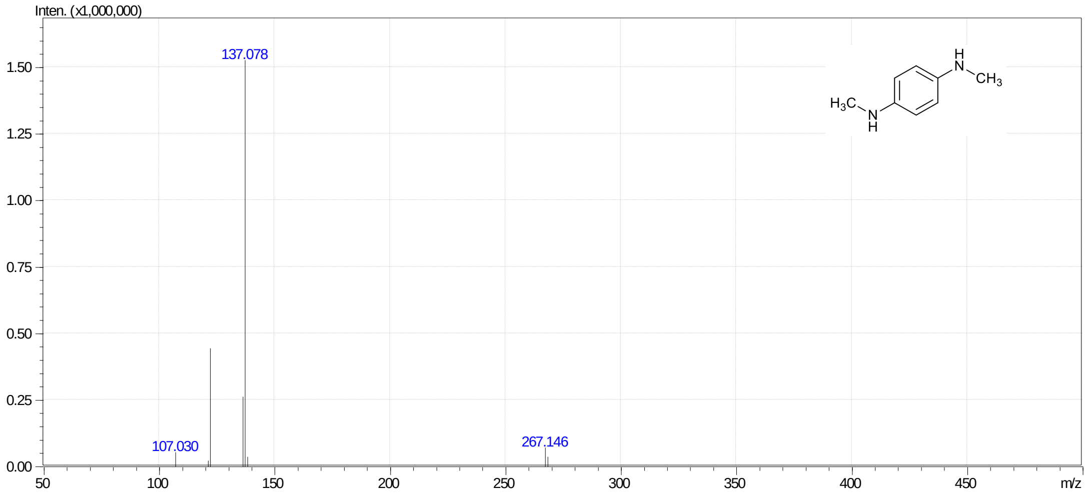

***N*-Methylcyclohexylamine (15c)**

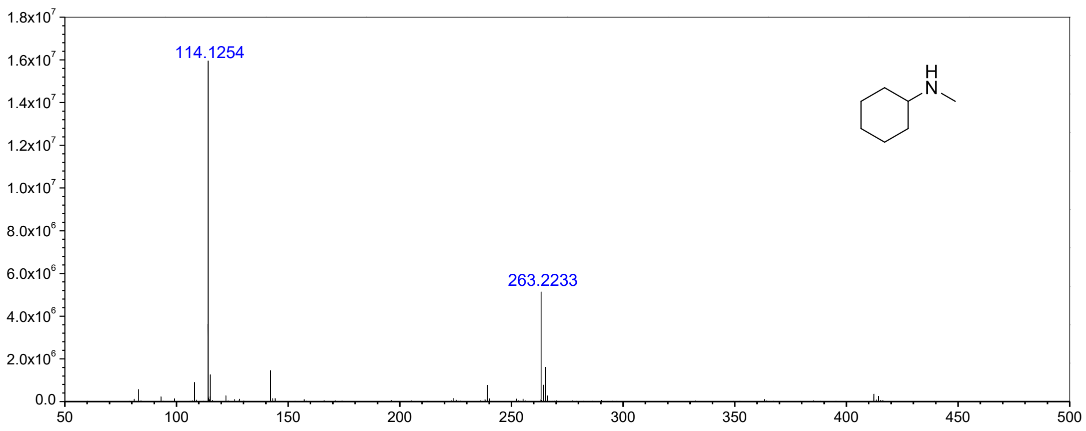

N-Methyl-4-chlorobenzenamine (16c)

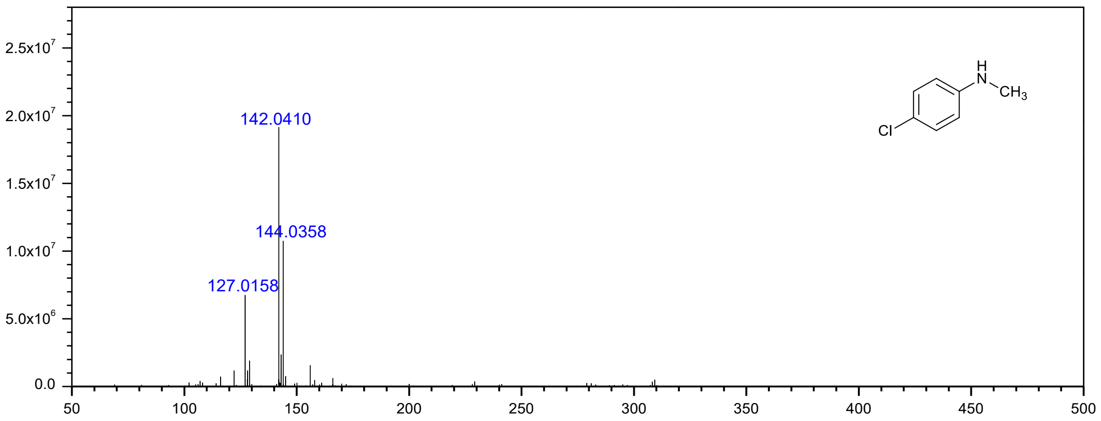

N-Methyl-4-(trifluoromethoxy)benzenamine (17c)

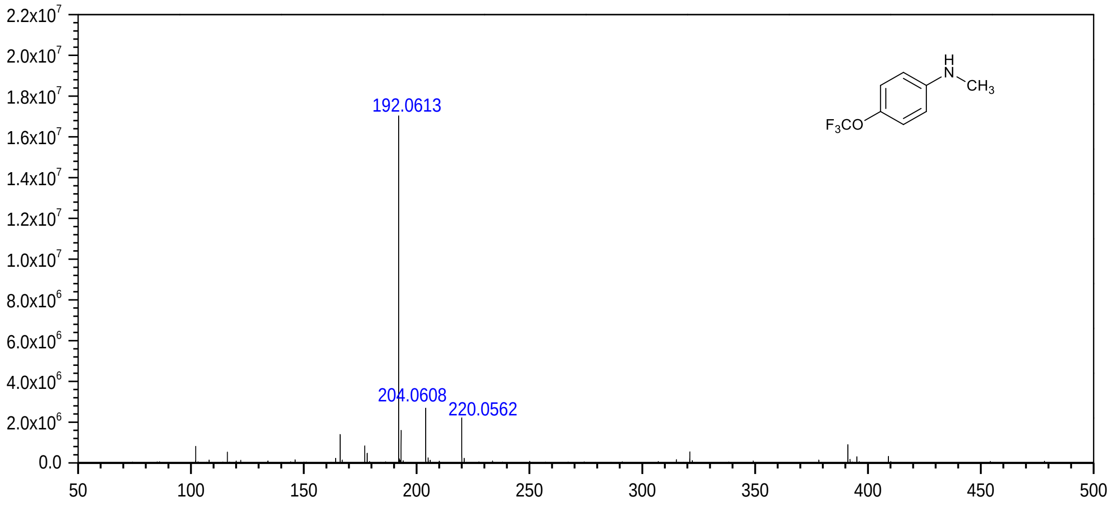

N-Methylnonylamine (18c)

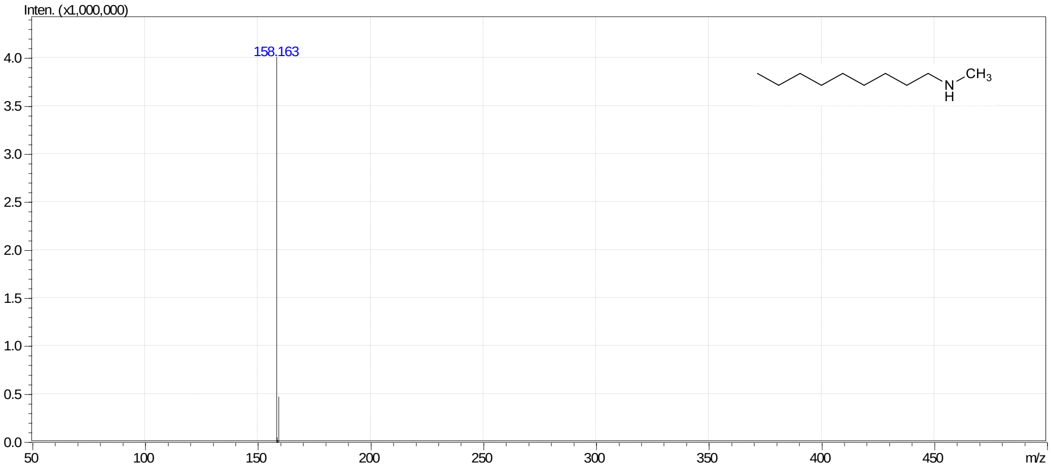

N-[5-(Methylamino)pentyl]acetamide (19c)

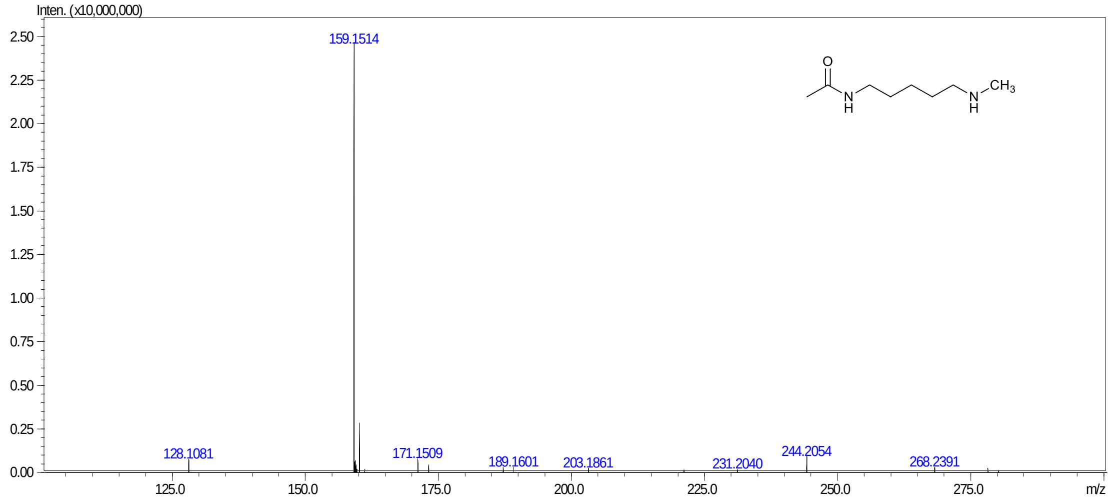

N-[4-(Methylamino)phenyl]acetamide (20c)

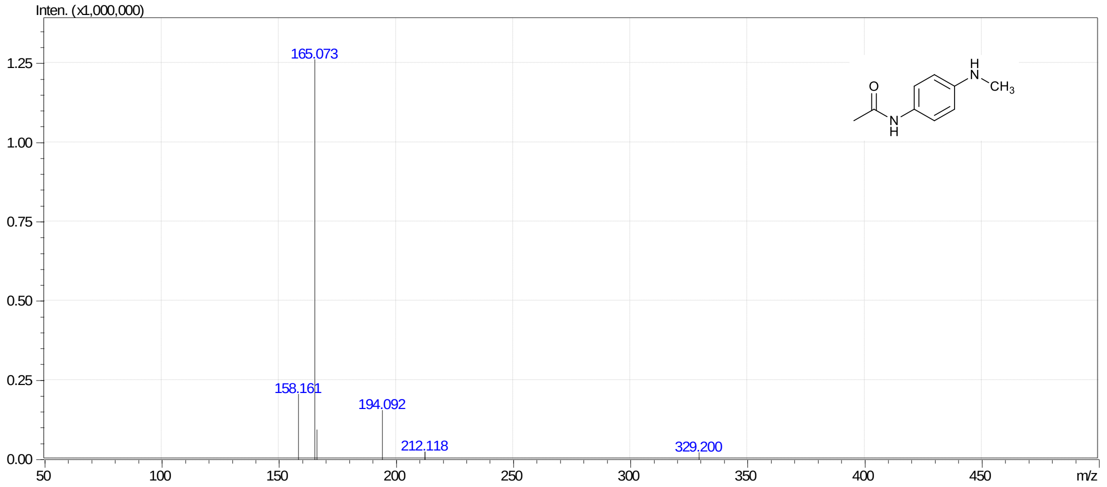

Supplement: File 1 — Experimental part. [file Beilstein_J_Org_Chem-18-1032-s001.pdf]
